# Ring Currents in Open-Shell Condensed Benzenoid Homologues of Some Extended Clar’s Goblets

**DOI:** 10.1021/acs.jpca.6c04818

**Published:** 2026-09-30

**Authors:** Timothy K. Dickens, Roger B. Mallion

**Affiliations:** Peterhouse, Cambridge CB2 1RD England, U.K.

## Abstract

Mishra et al. have recently synthesized a “π-extended Clar’s goblet”. This is a “concealed” non-Kekuléan condensed benzenoid hydrocarbon formed by (a) adding to Clar’s goblet some extra layers of benzenoid rings and (b) simultaneously changing the size of the moiety at the center of the structure from one hexagon to two hexagons—thereby changing that moiety from “benzenoid” to “naphthalenic”. This idea is generalized and the “topological” ring-currents in Clar’s goblet, and several of its homologues and oxidation states, are compared with those arising from these new structures. Quantitative calculations of topological ring-currents in these open-shell systems necessarily incorporate the technique of “Configurational State Averaging”. Their predictions are compared with those from pictorial current maps generated by more sophisticated *ab initio* and pseudo-π calculations. It is found that (a) adding or removing electrons from frontier nonbonding orbital shells makes no difference to the ring current; (b) the number of unpaired electrons in a given goblet depends on the number of layers in it, but not on its girth; (c) as the goblet becomes “fatter”, the current within the central moiety increases and the central rings become “less empty”; there is, therefore, a greater mixing of current between the upper and lower triangles within each goblet as their girths become larger; and (d) the Hückel–London “topological” calculations predict that the direction of the current around the periphery of any goblet will be counterclockwise (diamagnetic)—a conclusion that is invariably confirmed by calculations at the *ipso*-centric pseudo-π level.

## Graphical abstract

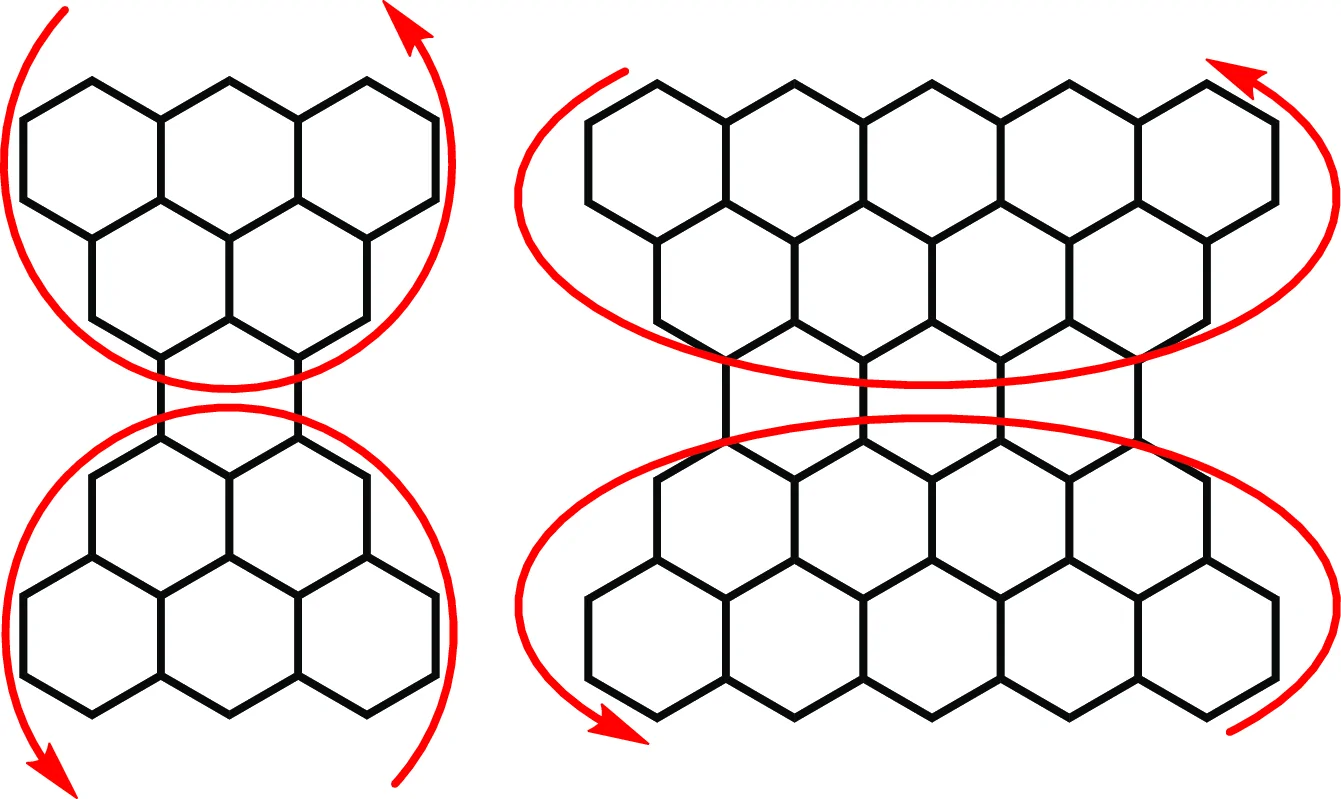

## Introduction

It is well-known that the Coulson–Rushbrooke “starring process” ^1–3^ may be applied to any condensed polybenzenoid hydrocarbon in order to divide its constituent carbon atoms into two classes: “starred” and “unstarred”. One of three outcomes may result in any particular case: (a) that the number of starred carbon atoms may equal the number of unstarred ones; (b) that there are more starred atoms than unstarred atoms; and (c) that there are more unstarred atoms than starred atoms. In cases (b) and (c), there is said to be a “sublattice imbalance.”^4^ When these cases (b) or (c) arise, no Kekulé structure can be devised for the conjugated system as a whole.^4^ This is illustrated, for example, by triangulene, depicted in Figure 1a of ref 4 as having a sublattice imbalance of 2. Because of this sublattice imbalance, triangulene has no Kekulé structures and it is, thereby, a radical (in this case it is a diradical).^4,5^ In such circumstances the conjugated system in question is described as being “obviously”^4^ non-Kekuléan. When case (a) arises, the species under study is most frequently a “normal” closed-shell benzenoid, hydrocarbon, which will possess a conventional Kekulé structure.^4^

Much more intriguing, however, are the so-called “concealed” non-Kekuléan polybenzenoid hydrocarbons.^4^ These species, despite having a sublattice imbalance of zero, cannot be depicted with a classical Kekulé structure and thus have an open-shell ground state. An example of this category of benzenoids (the diradical known as Clar’s goblet) is given in Figure 1a of Mishra et al.^4^ who have recently claimed^4^ that these concealed non-Kekuléan benzenoids “constitute an exceedingly small fraction of the total number of polybenzenoid hydrocarbons that can be constructed for a given number of benzenoid rings”. A classical example in this category of condensed benzenoids is the aforementioned Clar’s goblet which, as noted, is depicted in Figure 1a of ref 4, where it is shown to be a diradical. This structure first arose from speculations by Clar^5–7^ —including in personal correspondence with one of the present authors (RBM)^7^—more than 50 years ago. The species itself was recently synthesized on a Au(111) surface.^8^ In the course of the past decade, considerable advances have been made in the realization of iconic radical species such as triangulene and Clar’s goblet (albeit in solution or on metallic surfaces).^8–10^

**Figure 1:**
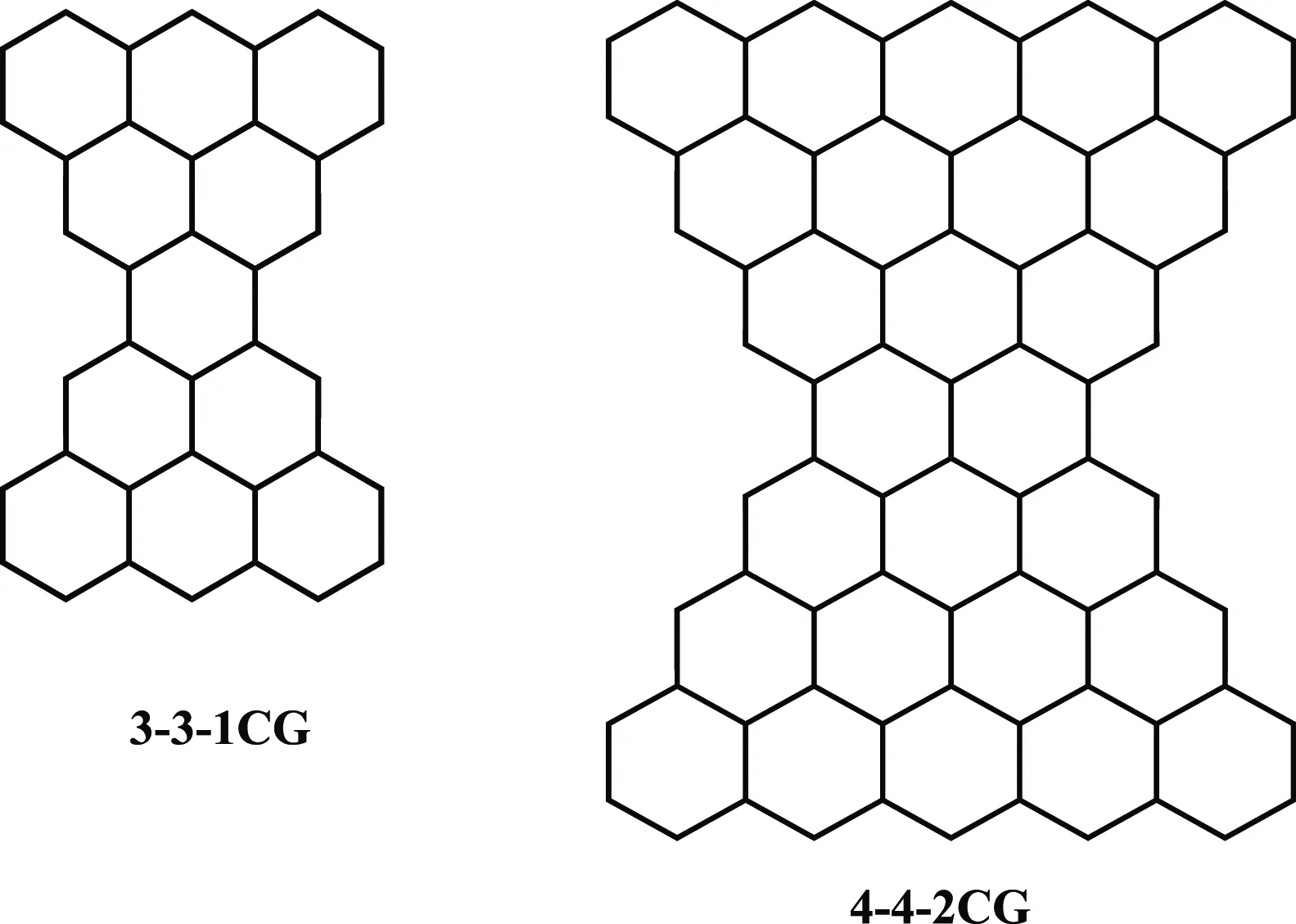
Molecular graphs,^14^ displaying carbon–carbon connectivity, of the diradical Clar’s goblet (left-hand side) and (right-hand side) the tetraradical called “π-extended Clar’s goblet”, introduced by Mishra et al.^4^ On the nomenclature adopted in the next section, these two benzenoid structures will be called **3–3–1CG** and **4–4–2CG**, respectively.

In the meantime, the present authors and our co-workers^11^ have extended the Hückel–London approach^12,13^ —which traditionally^14,15^ was designed to be applicable only to closed-shell systems—in order to enable it to calculate (“topological”^14^) π-electron ring-currents in *open*-*shell* conjugated systems such as triangulene and Clar’s goblet and related structures.^11,16^ This was done by invoking the technique that has become variously known as “Configurational State Averaging”, “Electron Smearing”, and “Fractional Occupation”.^16–18^ This procedure (described in §2.3 on p 10184 of ref 11) is said^11^ to go back to the work of Hartree,^18^ a century ago, but it has also been applied very recently.^11,16,17^ By adopting this clever device, we have been able to calculate the (“topological”)^14,15^ ring-currents in the previously inaccessible classic diradicals of triangulene (in ref 16) and (in ref 11) of Clar’s goblet.

Now, Mishra et al.^4^ have just introduced (and synthesized) what they call a “π-extended Clar’s goblet” by (a) adding to the paradigm structure extra layers of benzenoids and (b) simultaneously changing the size of the moiety at the center of the structure from one hexagon to two hexagons—thereby changing that moiety from “benzenoid” to “naphthalenic”. These changes are illustrated in Figure 1, and the demonstration that this structure is a tetraradical is to be found in Figure 1b of ref 4.

The investigations reported here will generalize the idea^4^ of a “π-extended Clar’s goblet” and will compare previous considerations^11,16^ of the topological ring-currents in Clar’s goblet itself—as well as several of its homologues and oxidation states—with those arising from these new structures. As a check on these quantitative calculations of *topological* ^14,15^ ring-currents, they will be compared with pictorial current-maps generated by more sophisticated *ab initio* ^19^ and pseudo-π^20^ calculations.

## Nomenclature and Ring Labeling

We first propose some nomenclature in order to try to standardize the discussion. We call Clar’s Goblet a **3–3–1CG**. That is, in both the upper and lower parts of the structure, it has three layers of benzene hexagons with a single benzene ring in the middle (those three layers *including* that middle moiety). Hence, the new structure with a “naphthalenic” moiety at its center that was recently synthesized and reported in ref 4 is **a 4–4–2CG** structure. See Figure 1 for illustrations of **3–3–1CG** (left-hand side) and **4–4–2CG** (right-hand side).

Throughout the rest of this paper, the ring-number schemes shown in Figures 2 and 3 will be used to denote symmetrically nonequivalent rings. Note that, as in Figure 3, conjugated CG structures that have ‘3′ as the third digit have a three-ringed “anthracenic” *motif* at or near the middle of the species. We shall call the number of benzenoid rings that are in that *motif* the *girth* of that structure. For example, in Figure 2: the girth of **5–5–1CG** is 1, the girth of **5–5–2CG** is 2, and the girth of **5–5–3CG** is 3.

**Figure 2:**
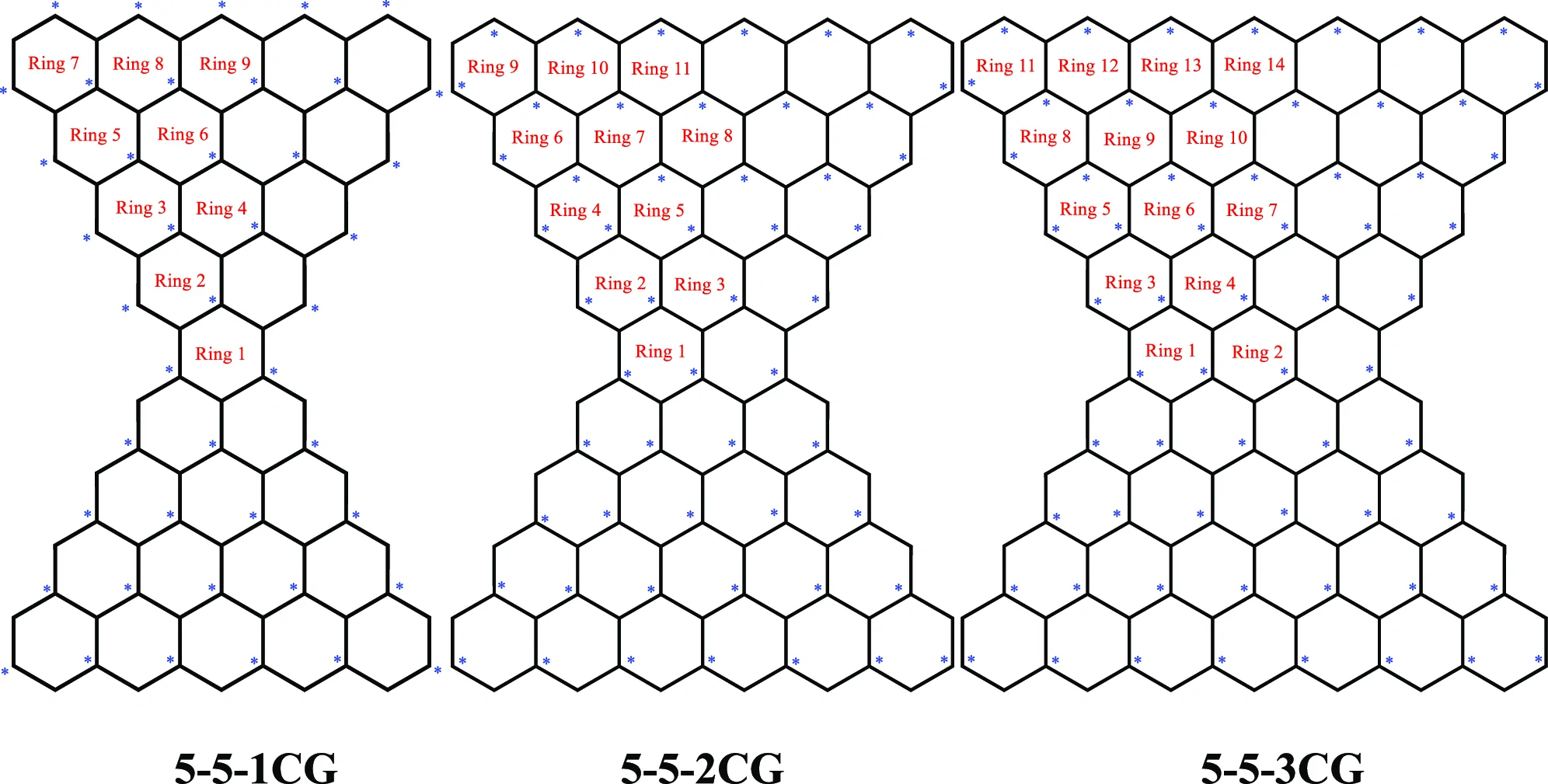
Structures **5–5–1CG**, **5–5–2CG**, and **5–5–3CG** and their ring labelings.

**Figure 3:**
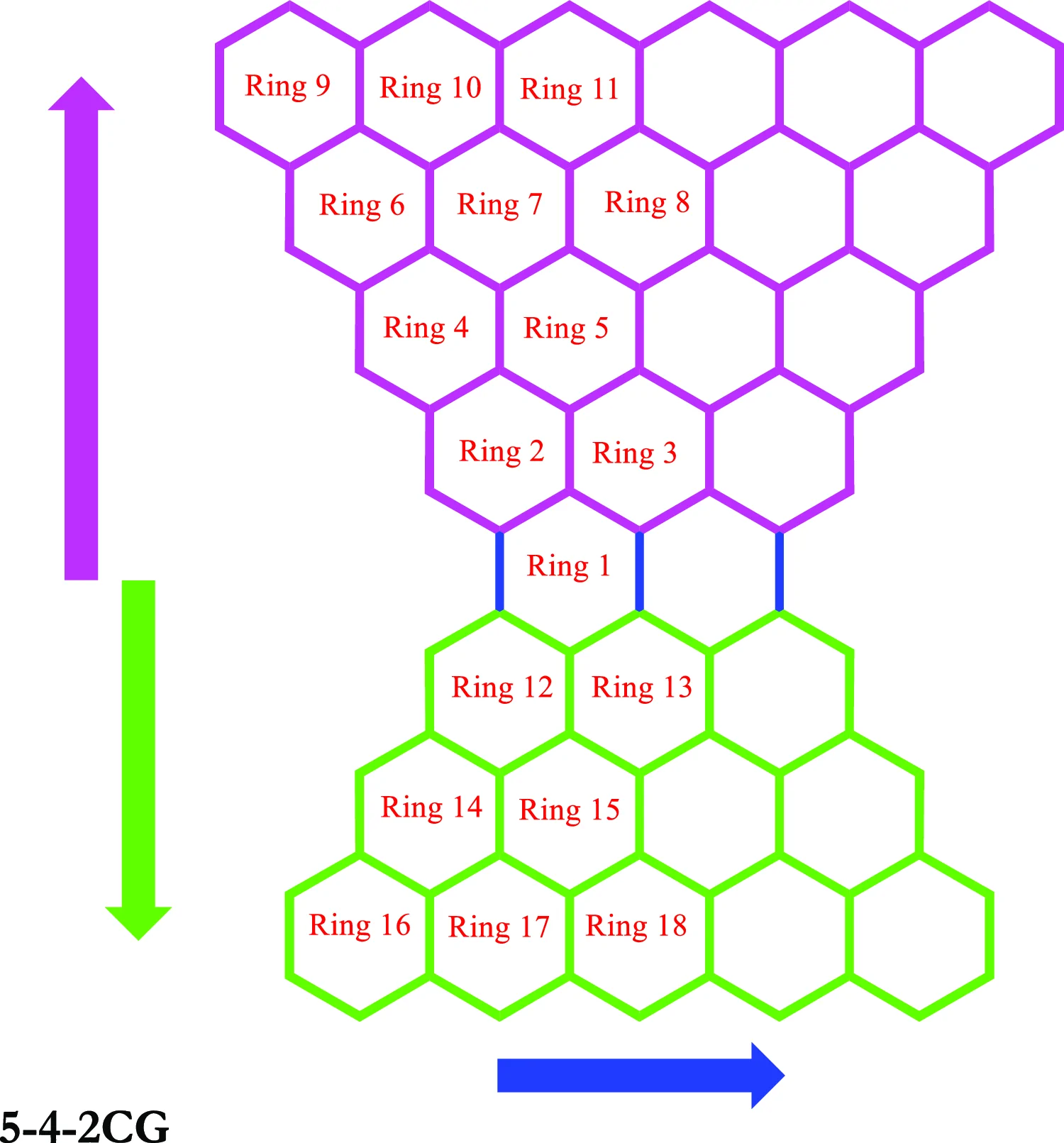
Structure **5–4–2CG** and its ring labelings. The purple arrow indicates the first layer set, the green arrow shows the second layer set, and the blue arrow indicates the width (“girth”) of the goblet.

Eleven structures are specifically examined here: (a) (in the body of the paper) **3–3–1CG [Clar’s goblet**], **4–4–1CG**, **5–5–1CG**, and **4–4–2CG** [the π-Extended Clar’s goblet of Mishra et al.^4^] and (in the Supporting Information) (b) **3–3–2CG**, **3–3–3CG**, **4–4–3CG**, **5–5–2CG**, **5–5–3CG**, and **5–4–2CG**, **5–3–2CG**. Data from the four goblets reported in the body of the paper and the seven to be found in the Supporting Information are cumulatively summarized in a table (Table 6) later in the paper *per se*.

## Methods of Calculation

The techniques adopted in this study are those described in detail in earlier studies,^11,16^ namely:

(a) The traditional Hückel–London^12,13^ “topological” ^14,15^ approach to ring currents, which gives rise to quantitative numerical values of ring-currents that strictly obey Kirchhoff’s Current Conservation Law.^21^ We report such calculated ring-currents expressed as a *ratio* to the ring-current intensity calculated, by the same method, for benzene; accordingly, ring currents expressed in this way are *dimensionless.*

In order to be able to treat open-shell systems by the various Hückel–London^12–15^ formalisms it is, however, mandatory to incorporate the device of “Configuration State Averaging”, referred to in the Introduction and explained in detail in the “Methods of Calculation” section of ref 16 and in §2.3 of ref 11.

One of the major tenets for a strict application of the topological ring-current approach is that the carbon-atom skeleton of the conjugated structure under consideration has to be (geometrically) planar.^14,15^ This was emphasized in our earlier studies of homologues of Clar’s goblet^11^ and triangulene.^11,16^ We, therefore, again stress that the molecular graphs of all the generalized Clar’s goblets considered here are essentially excisions from the infinite graphene plane^22^ (see Note 50 of ref 22); accordingly, they consist of arbitrary flat tessellations of regular hexagons of carbon atoms. Furthermore, neither do any of them have any attached peripheral hydrogen atoms that could cause steric interactions that might break the planarity. These flat structures of tessellated benzenoid rings are, therefore, ideal candidates for treatment by the classic Hückel–London^12–15^ approach.

(b) *Ab initio* ^19^ and pseudo-π formalisms^20^ that yield pictorial ring-current maps. Details are to be found in refs 19 and 20 .

## Discussion of Results

### Some Preliminary Studies

In order to recapitulate on previous results,^16^ topological ring-currents are shown in Table 1 for the classical (neutral) Clar’s goblet (**3–3–1CG**)—in the row colored in yellow—as well as in 12 of its oxidation states. (It should be noted that, in all the tables in this paper, positive quantities are printed in black and negative quantities are printed in red.) Also, throughout this paper, we are using the convention that a Counterclockwise flow of current is positive (diamagnetic) and a Clockwise (negative) flow of current is paramagnetic.

**Table 1:**
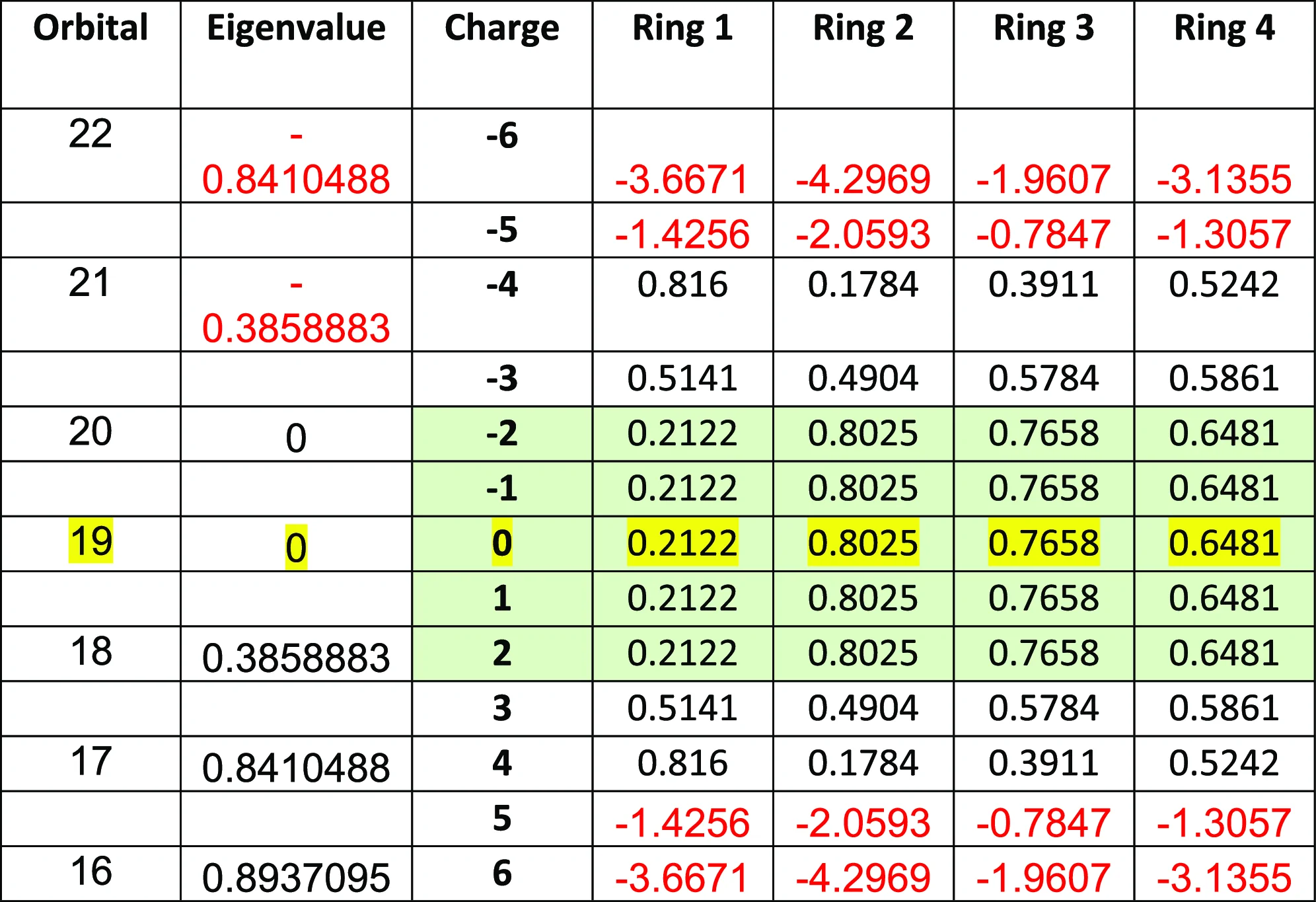
Topological Ring-Currents for the Classical (Neutral) Clar’s Goblet (3-3-1CG)—Indicated in the Row Colored Yellow—as well as in 12 of Its Oxidation States

In the neutral species, the LUMOs—the lowest unoccupied molecular orbitals—are degenerate and, as these are nonbonding orbitals (corresponding to zero eigenvalues of the **3–3–1CG** molecular graph), electrons in these orbitals do not contribute to the ring currents.^16,17^ As noted, adding electrons into these orbitals, or subtracting electrons from them, thus makes no difference to the calculated ring-currents; consequently, the remarkable observation may be documented that the ring currents calculated for the neutral species (illustrated in the row colored in yellow of Table 1) are the same as those encountered in the anion, the dianion, as well as the cation and the dication (all covering the green area of Table 1—cf. Table 2 of ref 16)). Because there are two degenerate orbitals and only two electrons in the frontier orbitals of the neutral species (the currents for which are shown in the yellow row of Table 1), neutral **3–3–1CG** is confirmed to be a diradical.

Table 2 similarly shows the calculated topological ring-currents for the neutral Goblet structure **4–4–1CG**, as well as those from −6 to +6 oxidation states. Here, there are four degenerate nonbonding orbitals and four electrons to be distributed among them in the neutral species. Once again, therefore, in the end, when the *Aufbau* Principle has been applied, and maximum spin multiplicity is assumed in accordance with Hund’s rule, the neutral species is predicted to be a tetraradical, having four unpaired electrons.^16^

**Table 2:**
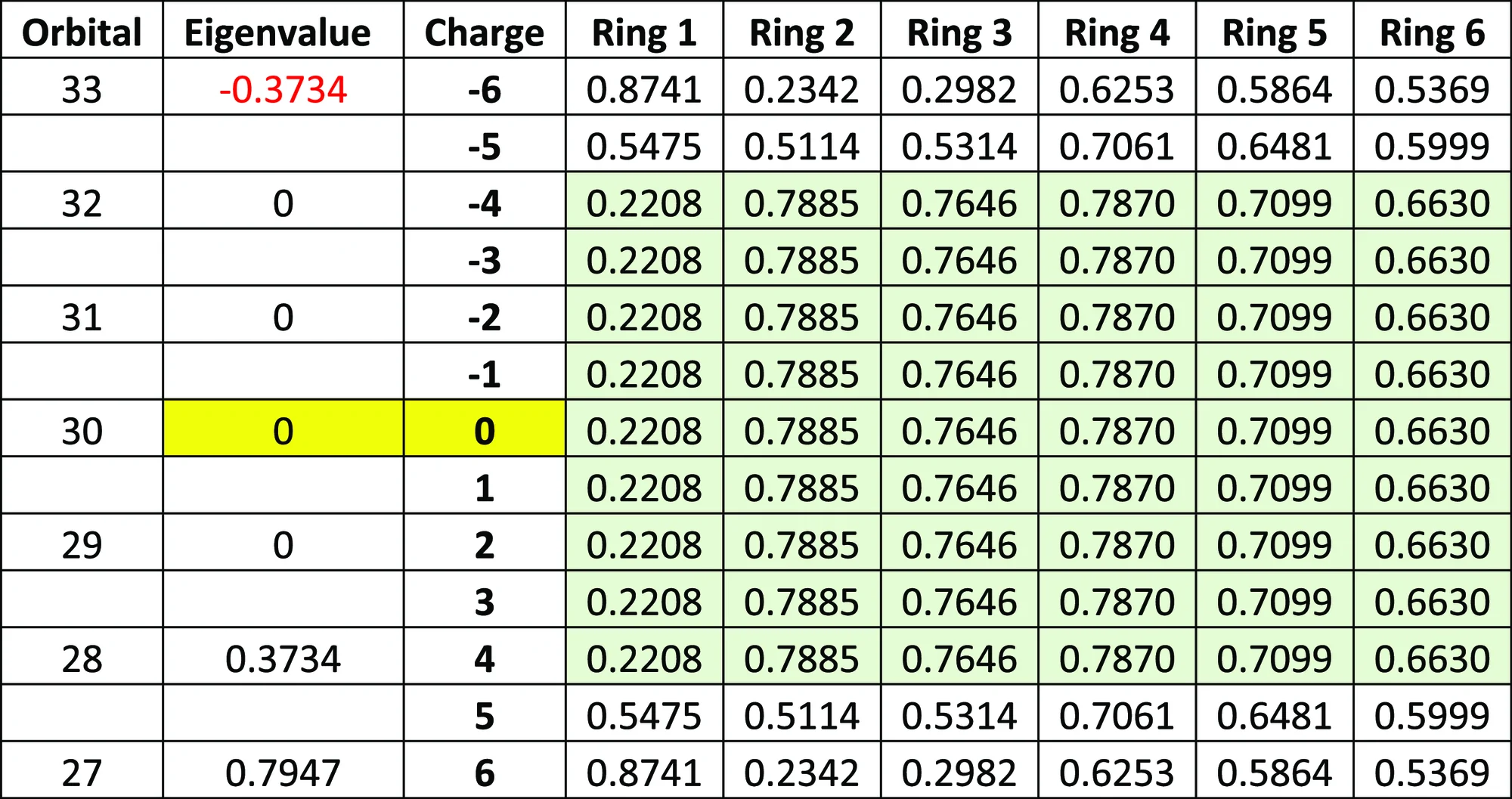
Topological Ring-Currents for the (Neutral) Goblet Structure 4-4-1CG—in the Row Colored in Yellow—as well as in 12 of Its Oxidation States

As expected, the ring-current pattern obtained when **5–5–1CG** is similarly treated is as displayed in Table 3. Six nonbonding orbitals are involved because of there being six zero eigenvalues in **5–5–1CG**’s molecular graph—that is, the molecular graph has a *nullity* of 6. The green and the yellow areas in Table 3 likewise illustrate the remarkable result that six anions (−1 to −6) and six cations (+1 to +6) all have precisely the same topological ring-currents, in all their rings, as does neutral **5–5–1CG**.

**Table 3:**
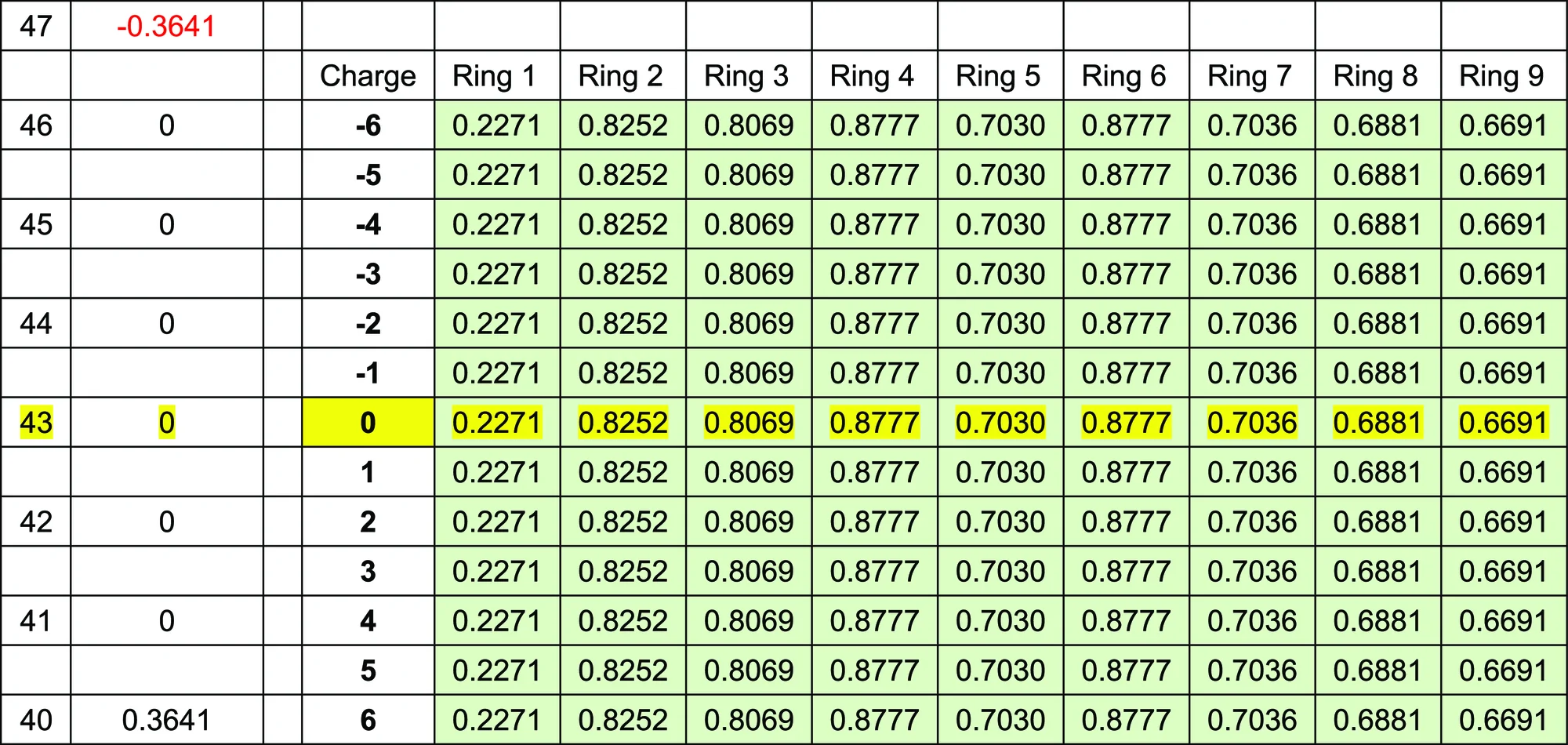
Topological Ring-Currents for the (Neutral) Goblet Structure 5-5-1CG—in the Row Colored in Yellow—as well as in 12 of Its Oxidation States

### A Complete Study on **4-4-2CG**

Having examined some preliminary examples, we now turn attention to the structure **4–4–2CG**: this is the species recently reported and synthesized by Mishra et al. in ref 4. The “2” in the structure’s code indicates that, unlike the structures of girth 1 examined so far, there is a naphthalenic *motif*, rather than a benzenoid one, in the middle of the structure. First, in Figure 4, we present the Hückel–London *bond* currents in neutral **4–4–2CG**. As is the case in general, these bond currents are entirely consistent, by virtue of Kirchhoff’s Laws^21^ of electrical networks, with the topological *ring*-currents that are displayed in the yellow row of Table 4. (It should be remarked that, for convenience of display in Figure 4, 4–4**-2CG** there is depicted rotated by 90° so that it is no longer in its “goblet” form but is now in what might be called its “bow-tie” form.)

**Figure 4:**
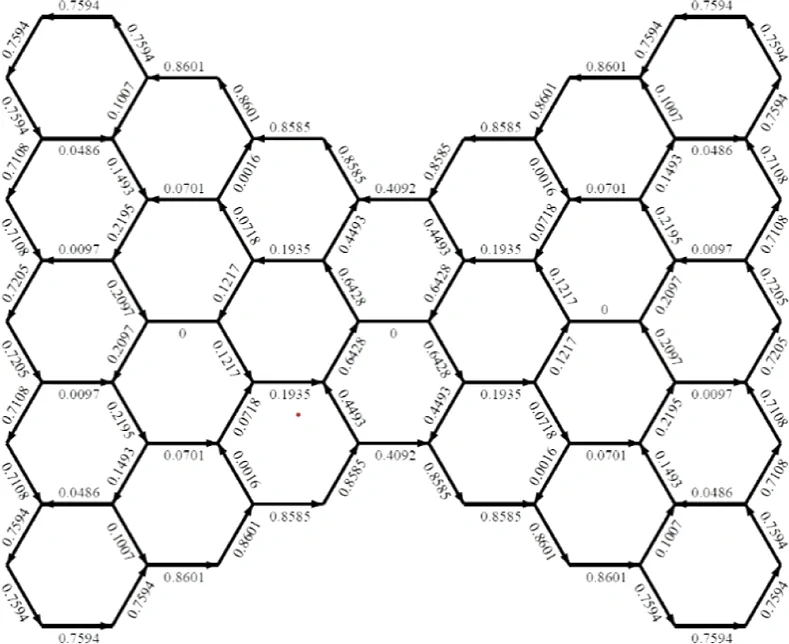
Hückel–London bond-current intensities^11–15^ in the π-Extended Clar’s Goblet **4–4–2CG** described by Mishra et al.^4^ These bond currents respect Kirchhoff’s Law of Conservation of Currents at junctions^21^ (in this context, the “junctions” are the carbon atoms).

**Table 4:**
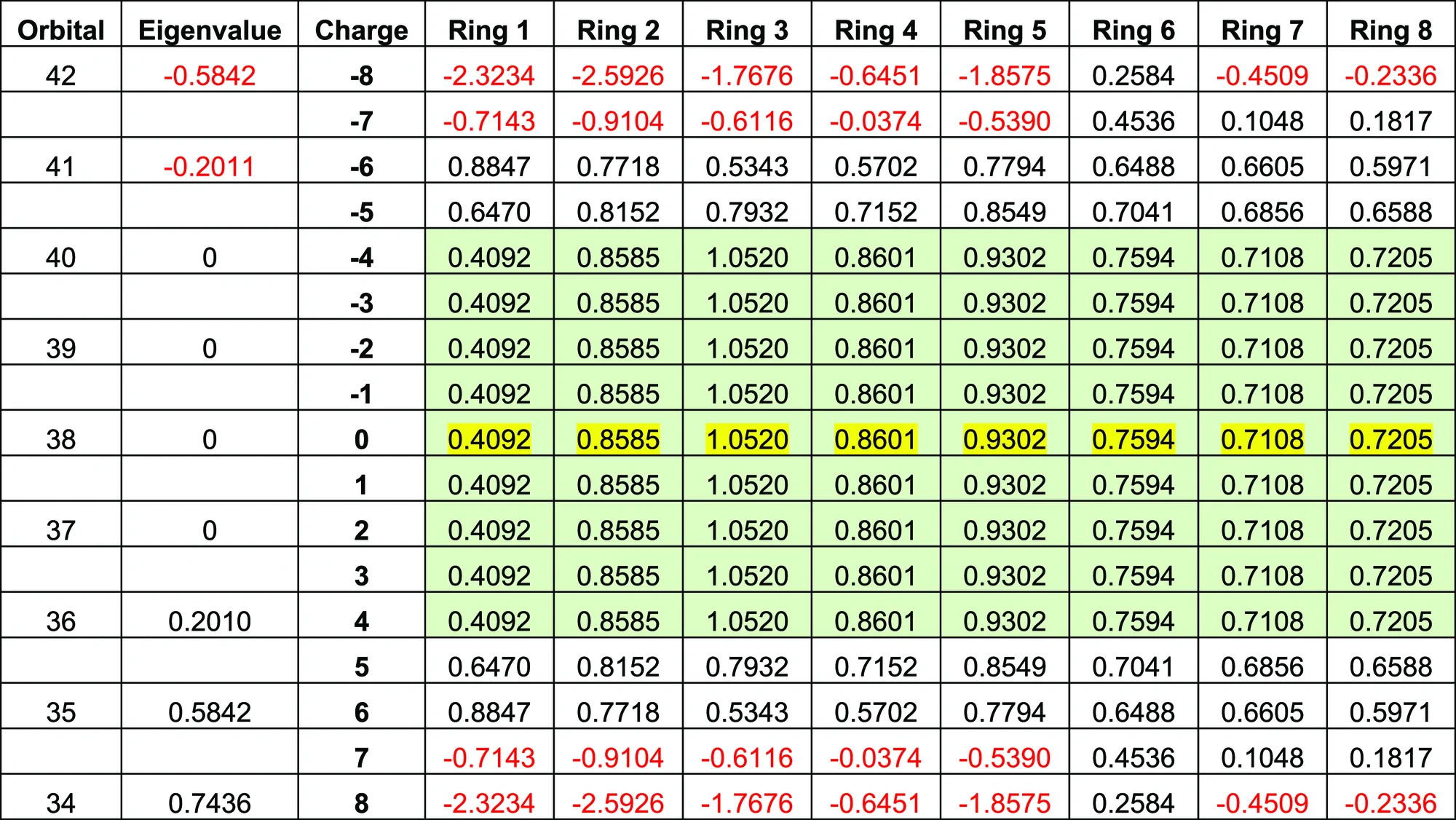
Topological Ring-Currents for the (Neutral) Goblet Structure 4-4-2CG—in the Row Colored in Yellow—as well as in Another 16 of Its Oxidation States

It is evident from Table 4 that adding electrons to, or removing electrons from, nonbonding frontier orbitals makes no difference to the computed ring-currents. This is seen on inspection of the yellow and green areas in Table 4. All of these extended Clar’s Goblets possess molecular graphs that are bipartite^1–3^ and, because of this, the contributions to the calculated ring-currents from each pair of orbitals above and below the HOMO—the highest occupied molecular orbital—of the neutral species are expected to be identical in size and opposite in sign, as per the Coulson–Rushbrooke theorem.^1–3^

It will also be noted that—as has long been observed in the central benzenoid ring in structures related to perylene^23–25^ —the central *motif* (which is a benzenoid ring in the case of perylene and a naphthalenic one in the case of **4–4–2CG**) bears a noticeably smaller ring-current, in the neutral species, than do the other rings. In other words, the naphthalene *motif* in the middle of **4–4–2CG** could be considered to be (relatively) “empty”.

Table 5 lists the difference in calculated ring-current for any given ring when changing from one oxidation state to the next. Generally, the differences come in pairs as there are two oxidation states associated with each molecular orbital. Notice how the differences are also paired and of opposite sign, again as per the Coulson–Rushbrooke Pairing theorem.^1–3^ This is indicated by the color of the background. However, it should be noted that eigenvalues of orbitals 37–40—see Table 4 for an illustration of these—do not change and are in fact zero in energy. Therefore, the calculated ring-current does not change from when the molecule is in the +4 oxidation state.

**Table 5:**
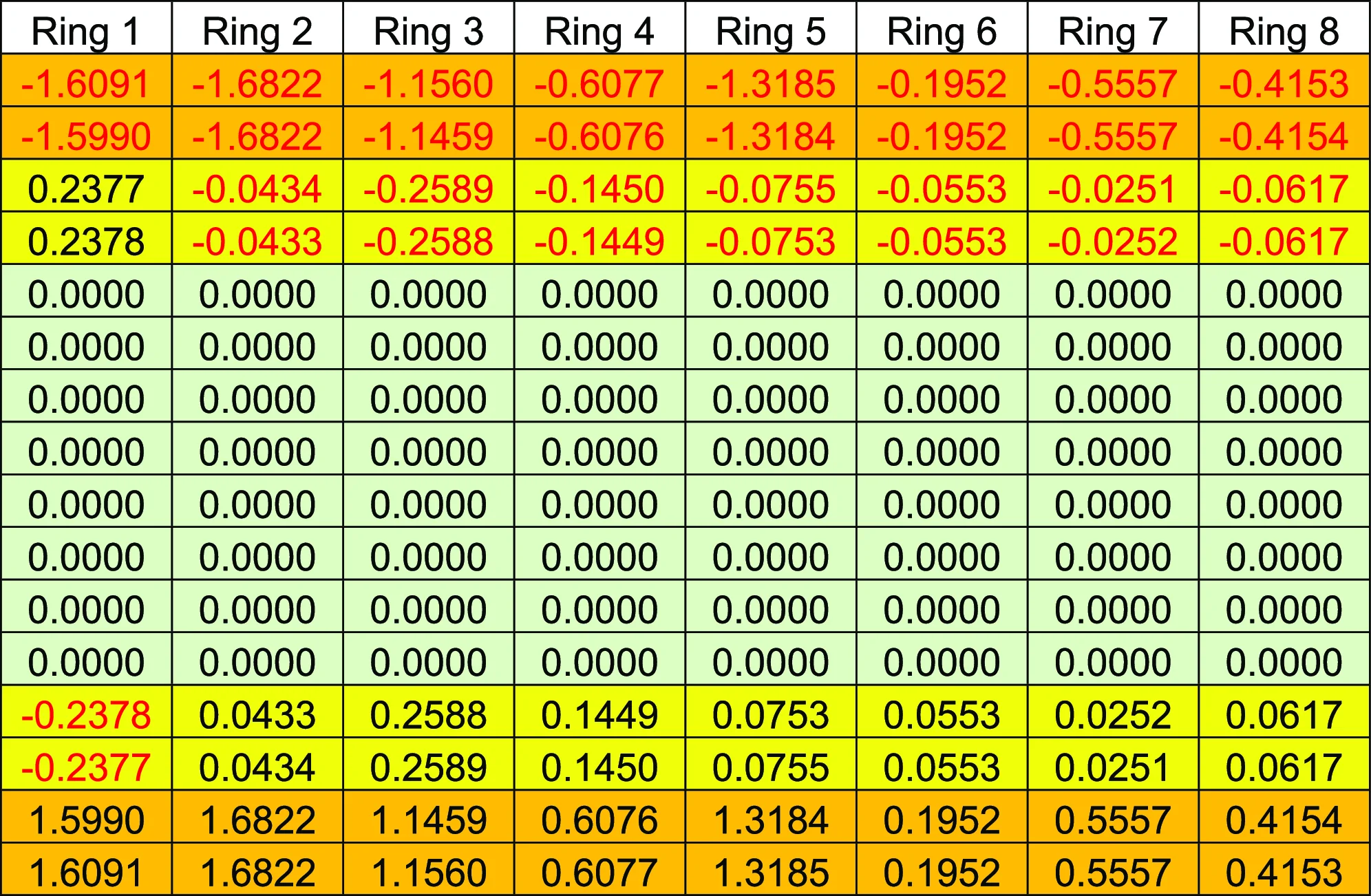
Difference for Any Given Ring Current between One Oxidation State of **4-4-2CG** and the Next

By means of the Hückel–London calculations,^11–15^ the following observations can be made as a result of having an extra set of benzene rings on the side of the structure:

Adding electrons to or removing electrons from the nonbonding frontier orbitals makes no difference to the computed ring currents.

This molecule—like all the others discussed in this paper—has a bipartite molecular-graph and, accordingly, the calculated ring-currents for the two systems with the charges +*q* and −*q* are identical within the Hückel–London model.^11–15^

There is the same pattern of ring currents as was observed in the original Clar’s Goblet,^11^ in that the central rings have a noticeably smaller ring-current than the other rings.^23–25^ In other words, a partially “empty” naphthalenic *motif* is evident in the middle of the structure **4–4–2CG**.

Figure 5 displays SYSMO *ipso*-centric pseudo-π calculations^19,20^ that were performed on the tetracation and tetraanion of **4–4–2CG**. This more restricted section of species arose because SYSMO may resolve only closed-shell calculations. The goal is to see if the observed ring-current qualitatively matches the Hückel–London results. The tetracation and tetraanion species were chosen as these represent a completely unfilled and filled frontier shell, respectively. Observations from Figure 5 are as follows:

1. The maps for the fully occupied and empty frontier orbitals look very similar.
2. The current flows counterclockwise (diatropic/diamagnetic) around the peripheral atoms with the central naphthalene moiety exhibiting little current.
3. The ring currents flow in two separate circuits: one around the top and the other around the bottom halves of the goblet.

**Figure 5:**
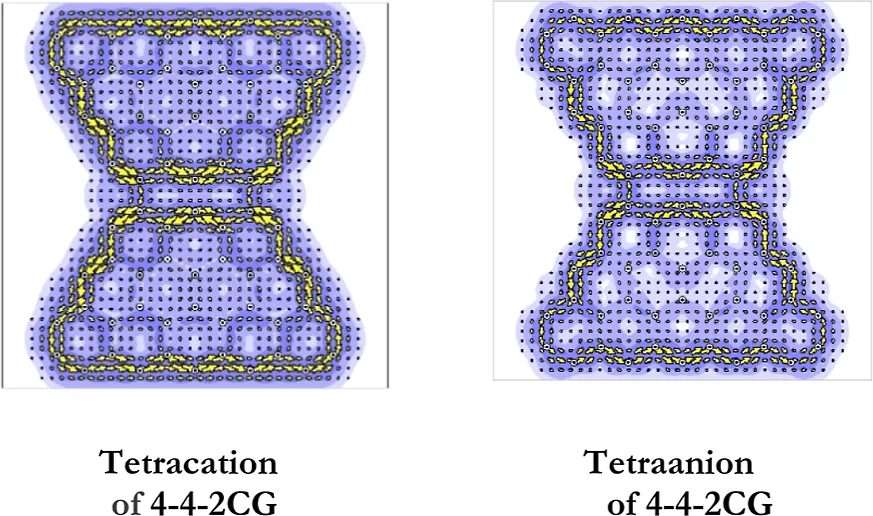
Current maps produced by the SYSMO program performing *ipso*-centric pseudo-π calculations^19,20^ on the tetracation and tetraanion of **4–4–2CG**. The yellow arrows indicate the direction of a given current flow and the density of them semiquantitatively denotes the intensity of such currents. On our conventions, currents flowing in the counterclockwise sense are diamagnetic, whereas clockwise ones are paramagnetic.

## Summary

A range of 11 differently sized, neutrally charged, goblets was examined via Hückel–London^12–15^ calculations. The results of these are given in the Main Paper and in the Supporting Information associated with it. Key features from these calculations are given in Table 6. What we are calling the “Bridge Current” in the right-hand column of Table 6 is the intensity of the current in the isolated bonds joining the top and bottom halves of the goblet.

**Table 6:** Summary of Key Features from Hückel–London^11–15^ Calculations on Eleven Neutral Goblet Species

goblet	# of orbitals	Frontier start	Frontier end	# of unpaired electrons in neutral	bridge current (Hückel–London)
**3–3–1**	38	19	20	2	0.2122
**3–3–2**	50	25	26	2	0.3878
**3–3–3**	62	31	32	2	0.4821
**4–4–1**	60	29	32	4	0.2208
**4–4–2**	76	37	40	4	0.4092
**4–4–3**	92	45	48	4	0.5112
**5–5–1**	86	41	46	6	0.2271
**5–5–2**	106	51	56	6	0.4299
**5–5–3**	126	61	66	6	0.5433
**5–3–2**	78	38	41	4	0.4084
**5–4–2**	91	44	48	5	0.4194

## Conclusions

1 As expected, adding electrons to, or removing electrons from, frontier nonbonding orbital shells makes no difference to the magnetically induced topological ring currents.^16,17,26^

2 The number of unpaired electrons in various goblets depends on the number of layers in a goblet but not on the girth of it.
3 When the number of layers is the same in the upper part of the goblet as it is in the lower part, the number of unpaired electrons can be predicted from that number of layers according to the following rule:

N=2L−4

where: *N* is the number of unpaired electrons and *L* is the unique number of layers in each of the upper and lower parts of the goblet (indicated by the Purple arrow and the Green arrow in Figure 2.) Note that, in our notation defined at the beginning of the paper, the layer associated with the central ring(s) is effectively counted twice. Thus, for a goblet with code *xy–z*CG—allowing for the case where *x* and *y* are *un*equal integers—the total number of layers (allowing for the narrowest layer to be counted twice) is *x* + *y*, and the number of nonbonding orbitals is *x* + *y*–4, implying the same number of unpaired electrons, according to Hund’s rule of maximum multiplicity.^27,28^

4 As the goblet becomes “fatter”—that is, as the central motif is changed from benzene to naphthalene to anthracene, etc.—the current within that central moiety increases and the central rings could, therefore, be said to be “less empty”. From the right-hand column of Table 6, it can be seen that (a) for structures of girth 1, the current in the central moiety is in the range of 0.20–0.23; (b) for girth 2, the bounds are 0.38–0.43; and (c) analogous currents in goblets with girth 3 are approximately between 0.48 and 0.55.
5 As with the Hückel–London calculations, computations at the *ipso*-centric pseudo-π level of approximation for fully closed or fully open frontier shells always yield a peripheral current that is counterclockwise, i.e., diamagnetic/diatropic.

## Supplementary Material



## Notes

Results of calculations of the various goblets discussed in the Main Paper but where the raw results were not reported; computations on other goblet species, not explicitly investigated in the Main Paper; pictorial illustrations of the 11 Kekulé structures studied, as well as their Frontier Orbitals; and Cartesian Coordinates for all the carbon atoms in the 11 goblet structures considered in this study (PDF)
