# Supplementary material for: Ring Currents in Open-Shell Condensed Benzenoid Homologues of Some Extended Clar’s Goblets
Source: J Phys Chem A. 2026 Sep 30;130(40):8065–71. doi: 10.1021/acs.jpca.6c04818 (PMC13656772; doi:10.1021/acs.jpca.6c04818)
Supplement: Supplementary file 1 [file jp6c04818_si_001.pdf]

Ring Currents in Open-Shell Condensed Benzenoid Homologues of some Extended Clar's Goblets

By

Timothy K. Dickens* and Roger B. Mallion

Peterhouse, Cambridge CB2 1RD, England, United Kingdom

Supplementary Information

This Supplementary Material documents (a) the results of calculations of the various goblets discussed in the Main Paper but where the raw results were not reported; (b) the computations on other goblet species, not explicitly considered in the Main Paper; (c) SCHEMES 1 and 2 pictorially illustrating the eleven Kekulé structure studied as well as their Frontier Orbitals; (d) the Cartesian |Coordinates for all the carbon atoms the structures considered in this study.

3-3-2CG

The diagram below depicts the bond-current map of neutral **3-3-2CG** calculated by means of the Hückel–London approach.[11–15]

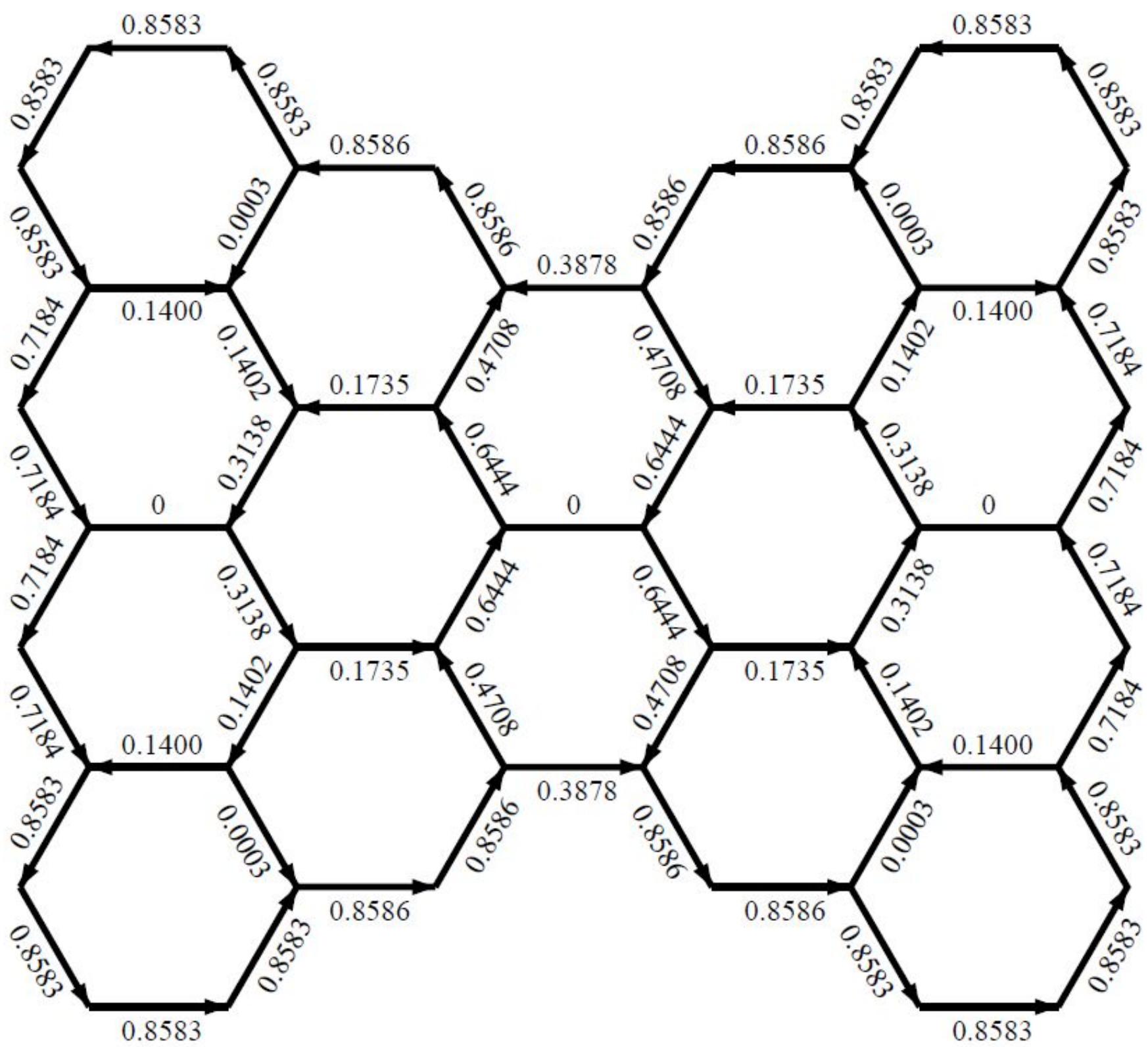

Figure S1.

Table S1. The table below displays ring-current intensities for various oxidation states of **3-3-2CG** and their associated eigenvalues.

Orbital	Eigenvalue	Charge	Ring 1	Ring 2	Ring 3	Ring 4	Ring 5
29	-0.8471	**-8**	-3.1208	-1.9563	-2.298	-0.9271	-2.385
		**-7**	-2.6764	-2.0848	-1.8772	-0.659	-1.4781
28	-0.6330	**-6**	-2.2319	-2.2132	-1.4564	-0.391	-0.5712
		**-5**	-0.7202	-0.7578	-0.4954	0.101	0.0083
27	-0.2092	**-4**	0.828	0.6975	0.4656	0.5929	0.5878
		**-3**	0.6079	0.7781	0.7489	0.7256	0.6531
26	0	**-2**	0.3878	0.8586	1.0322	0.8583	0.7184
		**-1**	0.3878	0.8586	1.0322	0.8583	0.7184
25	0	**0**	0.3878	0.8586	1.0322	0.8583	0.7184
		**1**	0.3878	0.8586	1.0322	0.8583	0.7184
24	0.2092	**2**	0.3878	0.8586	1.0322	0.8583	0.7184
		**3**	0.6079	0.7781	0.7489	0.7256	0.6531
23	0.6330	**4**	0.828	0.6975	0.4656	0.5929	0.5878
		**5**	-0.7202	-0.7578	-0.4954	0.101	0.0083
22	0.8471	**6**	-2.2319	-2.2132	-1.4564	-0.391	-0.5712
		**7**	-2.6764	-2.0848	-1.8772	-0.659	-1.4781
21	0.8505	**8**	-3.1208	-1.9563	-2.298	-0.9271	-2.385

Table S2. The table below shows the difference for any given ring current between one oxidation state of **3-3-2CG** and the next.

Ring 1	Ring 2	Ring 3	Ring 4	Ring 5
-0.4444	0.1285	-0.4208	-0.2681	-0.9069
-0.4445	0.1284	-0.4208	-0.2680	-0.9069
-1.5117	-1.4554	-0.9610	-0.4920	-0.5795
-1.5482	-1.4553	-0.9610	-0.4919	-0.5795
0.2201	-0.0806	-0.2833	-0.1327	-0.0653
0.2201	-0.0805	-0.2833	-0.1327	-0.0653
0.0000	0.0000	0.0000	0.0000	0.0000
0.0000	0.0000	0.0000	0.0000	0.0000
0.0000	0.0000	0.0000	0.0000	0.0000
0.0000	0.0000	0.0000	0.0000	0.0000
-0.2201	0.0805	0.2833	0.1327	0.0653
-0.2201	0.0806	0.2833	0.1327	0.0653
1.5482	1.4553	0.9610	0.4919	0.5795
1.5117	1.4554	0.9610	0.4920	0.5795
0.4445	-0.1284	0.4208	0.2680	0.9069
0.4444	-0.1285	0.4208	0.2681	0.9069

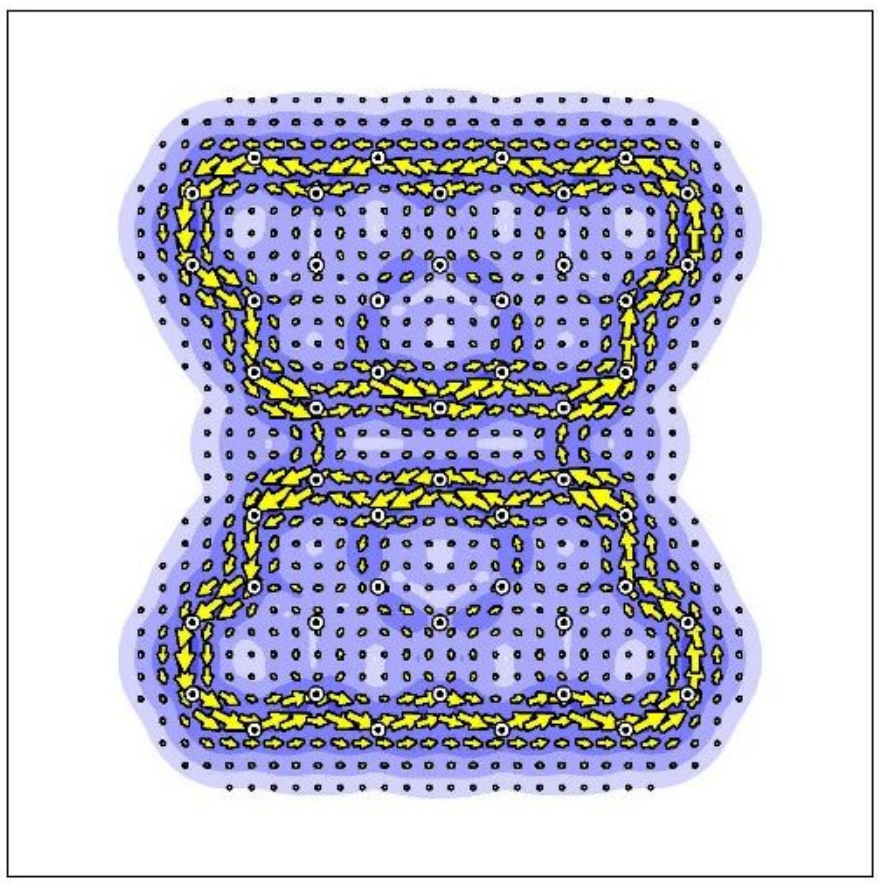

Dication of 3-3-2CG

Figure S2.

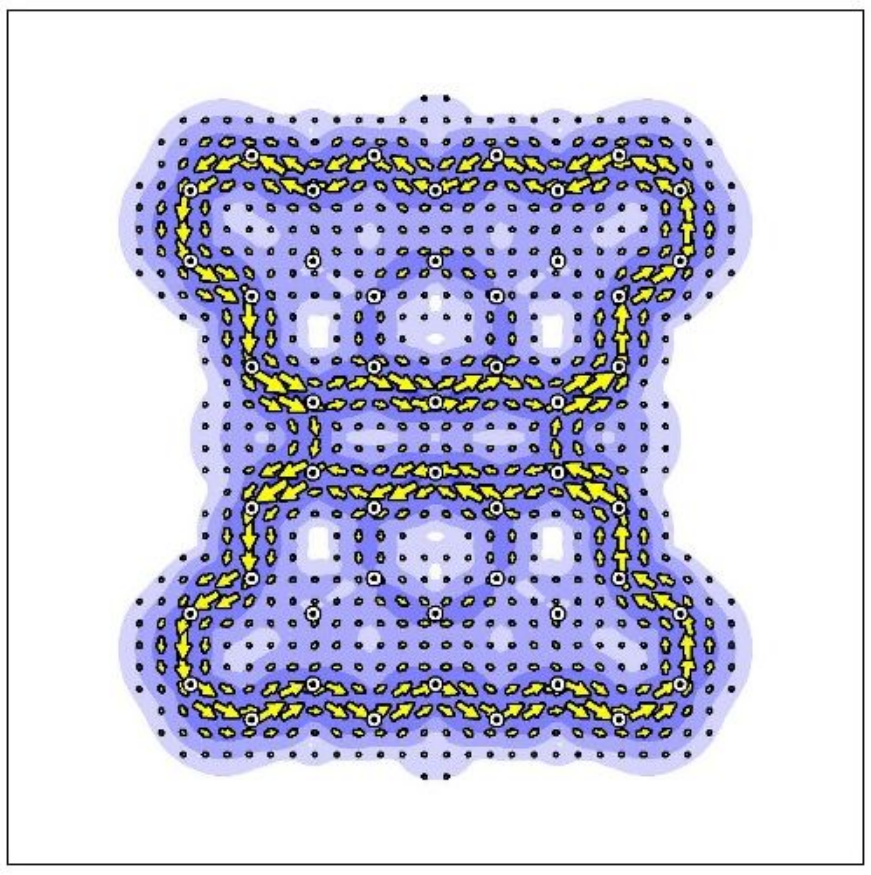

Dianion of 3-3-2CG

Figure S3.

SYSMO *ipso*-centric pseudo-π calculations were performed on the dication and dianion species. The goal was to see if the observed ring-current matches the Hückel–London[11–15] results. The tetracation dication and dianion tetraanion species were chosen as these represent a completely unfilled and filled frontier shell, respectively.

The diagrams above show SYSMO[19] *ipso*-centric pseudo-π[20] calculations performed on the tetracation and tetraanion of **3-3-2CG**.

Observations:

1. The maps for the fully occupied and empty frontier orbitals are very similar.
2. The current flows anticlockwise (diatropic/diamagnetic) around the peripheral atoms with the central naphthalene exhibiting little current.

3-3-3CG

The diagram below depicts the bond-current map of neutral **3-3-3CG** calculated by means of the Hückel–London approach.[11–15]

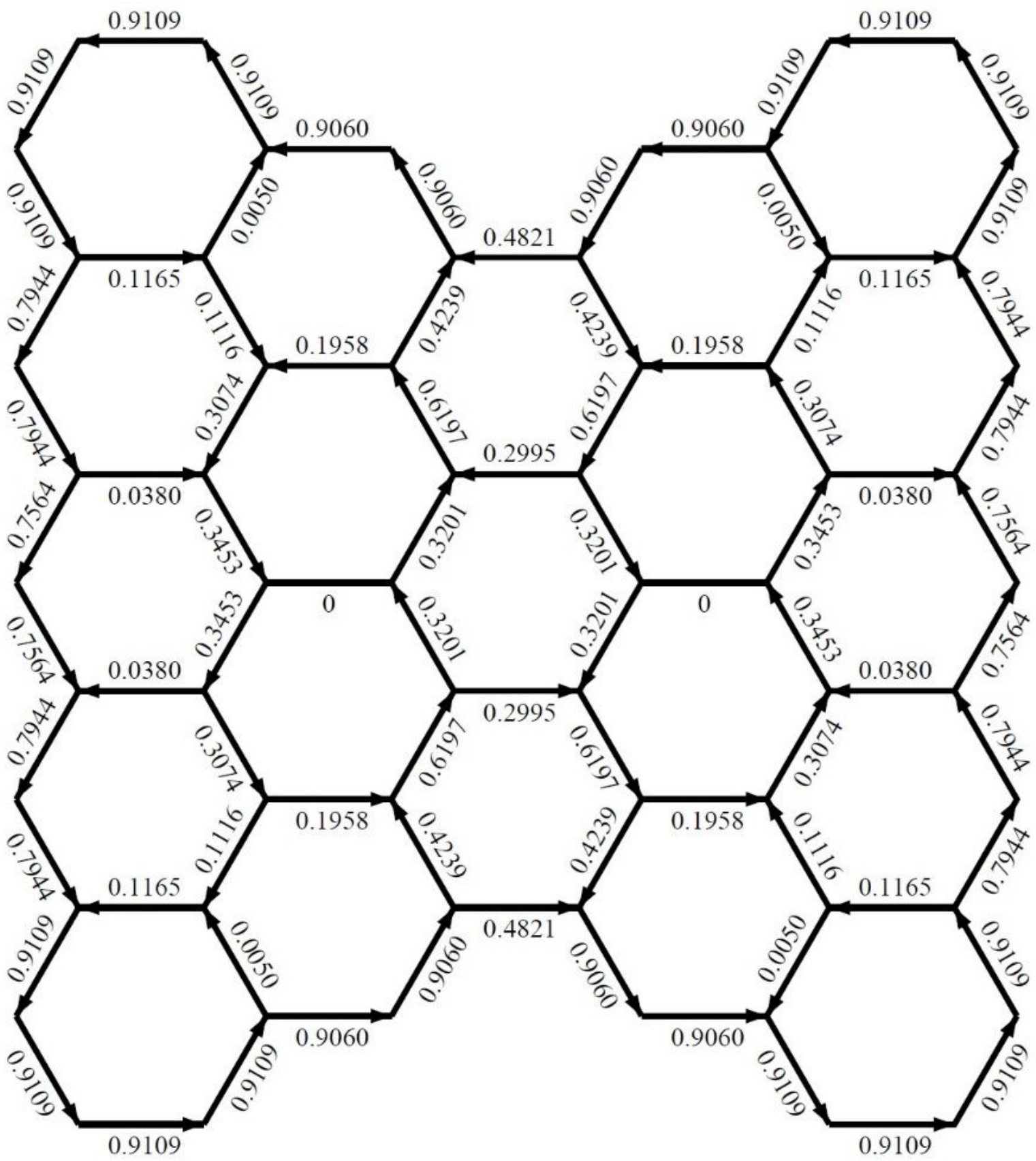

Figure S4.

Table S3. The table below displays ring-current intensities for various oxidation states of **3-3-3CG** and their associated eigenvalues.

Orbital	Eigenvalue	Charge	Ring 1	Ring 2	Ring 3	Ring 4	Ring 5	Ring 6	Ring 7
35	-0.7693	**-8**	1.6949	2.865	0.9454	-0.0623	-0.5326	-0.5225	-0.2348
		**-7**	0.8117	0.6398	0.1704	-0.2497	-0.2058	-0.1477	0.0653
34	-0.4487	**-6**	-0.0715	-1.5853	-0.6045	-0.4371	0.121	0.2271	-0.1041
		**-5**	0.3223	-0.1562	0.1535	0.1851	0.4296	0.4376	0.3097
33	-0.1146	**-4**	0.716	1.2729	0.9114	0.8072	0.7382	0.6481	0.7236
		**-3**	0.5991	1.0273	0.9087	0.9545	0.8246	0.7213	0.74
32	0.0000	**-2**	0.4821	0.7816	0.906	1.1018	0.9109	0.7944	0.7564
		**-1**	0.4821	0.7816	0.906	1.1018	0.9109	0.7944	0.7564
31	0.0000	**0**	0.4821	0.7816	0.906	1.1018	0.9109	0.7944	0.7564
		**1**	0.4821	0.7816	0.906	1.1018	0.9109	0.7944	0.7564
30	0.1146	**2**	0.4821	0.7816	0.906	1.1018	0.9109	0.7944	0.7564
		**3**	0.5991	1.0273	0.9087	0.9545	0.8246	0.7213	0.74
29	0.4487	**4**	0.716	1.2729	0.9114	0.8072	0.7382	0.6481	0.7236
		**5**	0.3223	-0.1562	0.1535	0.1851	0.4296	0.4376	0.3097
28	0.7693	**6**	-0.0715	-1.5853	-0.6045	-0.4371	0.121	0.2271	-0.1041
		**7**	0.8117	0.6398	0.1704	-0.2497	-0.2058	-0.1477	0.0653
27	0.7988	**8**	1.6949	2.865	0.9454	-0.0623	-0.5326	-0.5225	-0.2348

Table S4. The table below shows the difference for any given ring-current between one oxidation state of **3-3-3CG** and the next.

Ring 1	Ring 2	Ring 3	Ring 4	Ring 5	Ring 6	Ring 7
0.8832	2.2252	0.7750	0.1874	-0.3268	-0.3748	-0.3001
0.8832	2.2251	0.7749	0.1874	-0.3268	-0.3748	0.1694
-0.3938	-1.4291	-0.7580	-0.6222	-0.3086	-0.2105	-0.4138
-0.3937	-1.4291	-0.7579	-0.6221	-0.3086	-0.2105	-0.4139
0.1169	0.2456	0.0027	-0.1473	-0.0864	-0.0732	-0.0164
0.1170	0.2457	0.0027	-0.1473	-0.0863	-0.0731	-0.0164
0.0000	0.0000	0.0000	0.0000	0.0000	0.0000	0.0000
0.0000	0.0000	0.0000	0.0000	0.0000	0.0000	0.0000
0.0000	0.0000	0.0000	0.0000	0.0000	0.0000	0.0000
0.0000	0.0000	0.0000	0.0000	0.0000	0.0000	0.0000
-0.1170	-0.2457	-0.0027	0.1473	0.0863	0.0731	0.0164
-0.1169	-0.2456	-0.0027	0.1473	0.0864	0.0732	0.0164
0.3937	1.4291	0.7579	0.6221	0.3086	0.2105	0.4139
0.3938	1.4291	0.7580	0.6222	0.3086	0.2105	0.4138
-0.8832	-2.2251	-0.7749	-0.1874	0.3268	0.3748	-0.1694
-0.8832	-2.2252	-0.7750	-0.1874	0.3268	0.3748	0.3001

The diagrams below show SYSMO[19] *ipso*-centric pseudo-π[20] calculations performed on the tetracation and tetraanion of **3-3-3CG**.

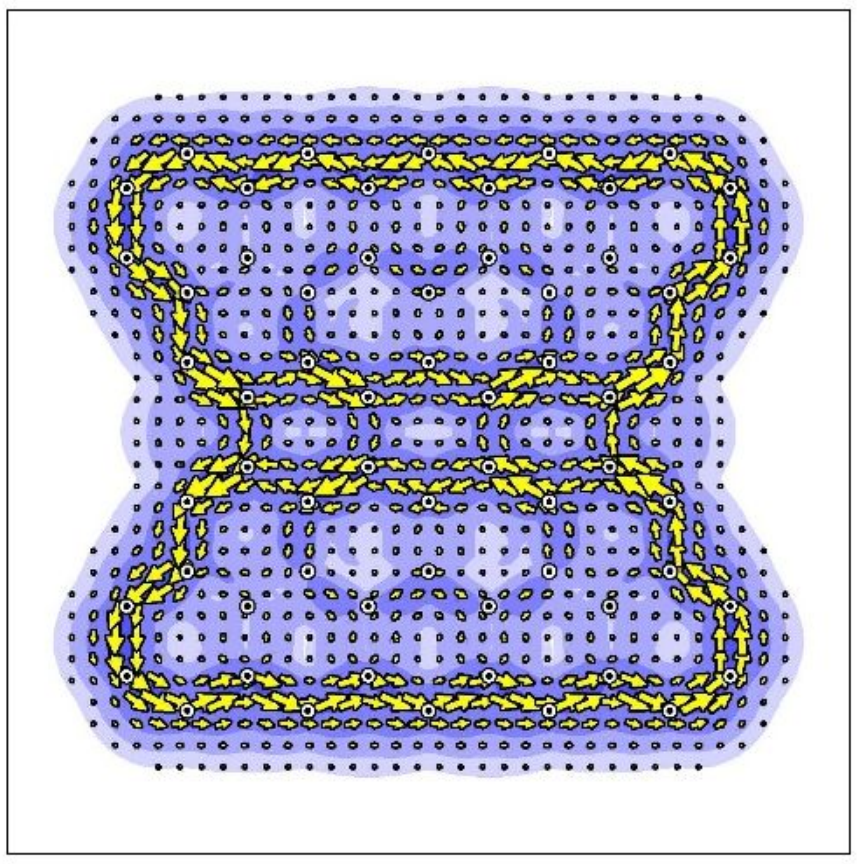

Dication of 3-3-3CG

Figure S5.

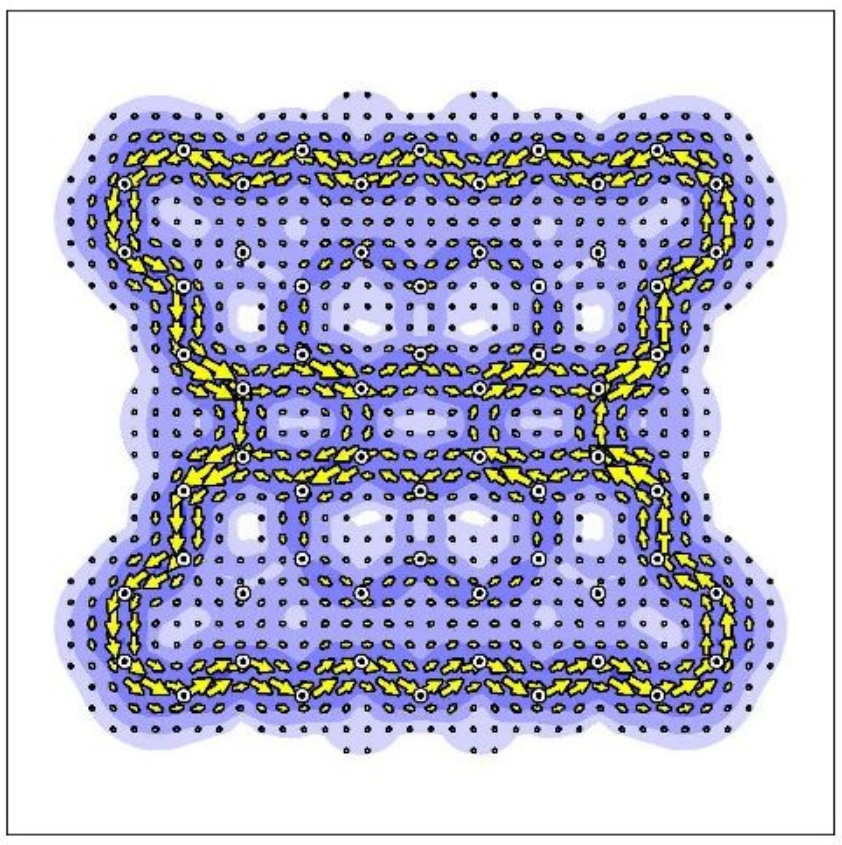

Dianion of 3-3-3CG

Figure S6.

4-4-3CG

The diagram below depicts the bond-current map of neutral **4-4-3CG** calculated by means of the Hückel–London approach.[11–15]

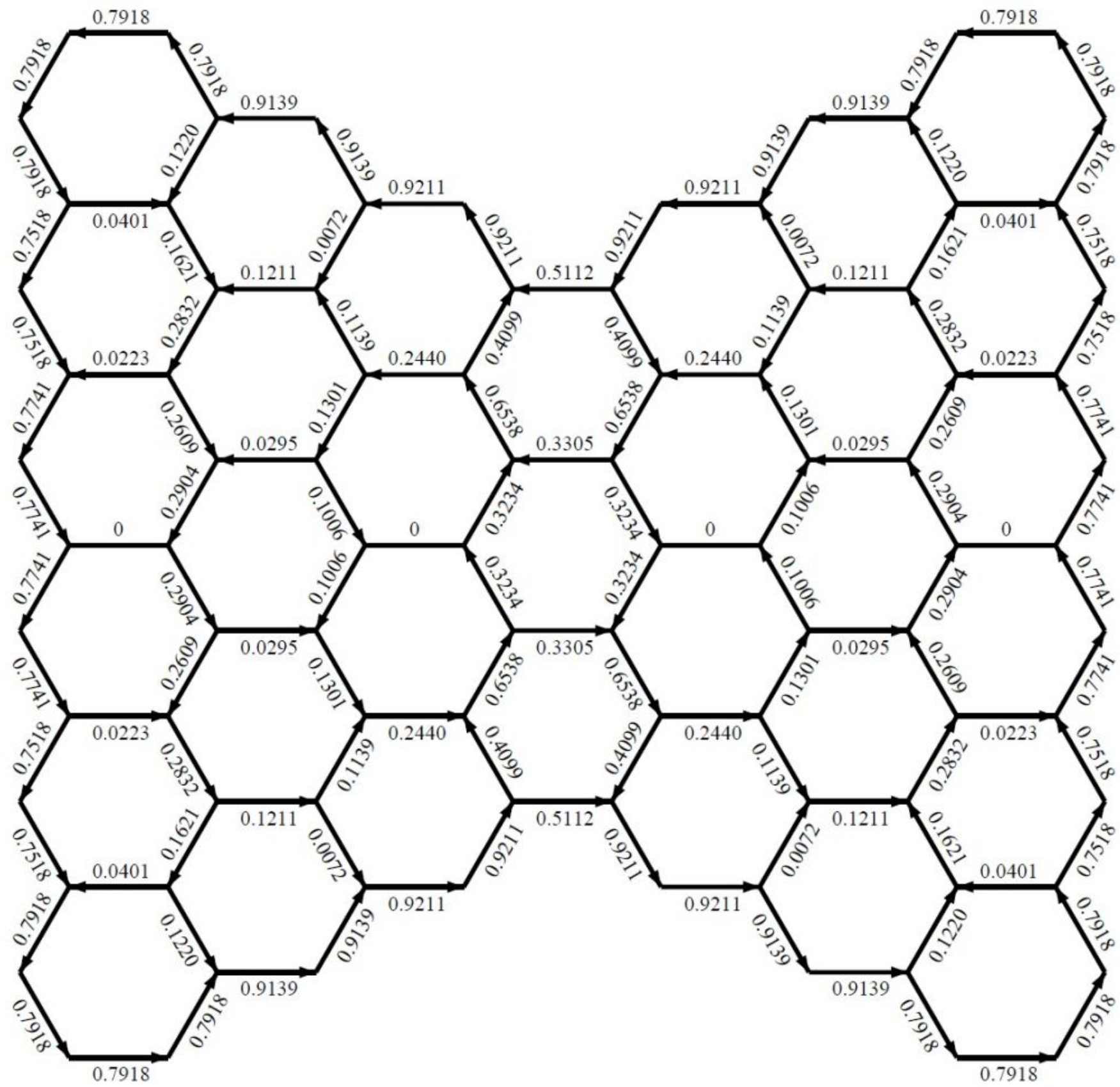

Figure S7.

Table S5. The table below displays ring-current intensities for various oxidation states of **4-4-3CG** and their associated eigenvalues.

Orbital	Eigenvalue	Charge	Ring 1	Ring 2	Ring 3	Ring 4	Ring 5	Ring 6	Ring 7	Ring 8	Ring 9	Ring 1
50	-0.40938	**-8**	0.0799	-1.4432	-0.6069	-0.4626	0.0041	0.1194	-0.5085	0.5273	0.2870	0.283
		**-7**	0.4187	-0.0329	0.1884	0.2403	0.3769	0.4926	0.2692	0.6124	0.5041	0.491
49	-0.10871	**-6**	0.7576	1.3775	0.9838	0.9233	0.7497	0.8658	1.0468	0.6976	0.7211	0.700
		**-5**	0.6344	1.1096	0.9524	1.0442	0.8318	0.9504	1.0556	0.7447	0.7364	0.737
48	0	**-4**	0.5112	0.8417	0.9211	1.1651	0.9139	1.0350	1.0645	0.7918	0.7518	0.774
		**-3**	0.5112	0.8417	0.9211	1.1651	0.9139	1.0350	1.0645	0.7918	0.7518	0.774
47	0	**-2**	0.5112	0.8417	0.9211	1.1651	0.9139	1.0350	1.0645	0.7918	0.7518	0.774
		**-1**	0.5112	0.8417	0.9211	1.1651	0.9139	1.0350	1.0645	0.7918	0.7518	0.774
46	0	**0**	0.5112	0.8417	0.9211	1.1651	0.9139	1.0350	1.0645	0.7918	0.7518	0.774
		**1**	0.5112	0.8417	0.9211	1.1651	0.9139	1.0350	1.0645	0.7918	0.7518	0.774
45	0	**2**	0.5112	0.8417	0.9211	1.1651	0.9139	1.0350	1.0645	0.7918	0.7518	0.774
		**3**	0.5112	0.8417	0.9211	1.1651	0.9139	1.0350	1.0645	0.7918	0.7518	0.774
44	0.108705	**4**	0.5112	0.8417	0.9211	1.1651	0.9139	1.0350	1.0645	0.7918	0.7518	0.774
		**5**	0.6344	1.1096	0.9524	1.0442	0.8318	0.9504	1.0556	0.7447	0.7364	0.737
43	0.409381	**6**	0.7576	1.3775	0.9838	0.9233	0.7497	0.8658	1.0468	0.6976	0.7211	0.700
		**7**	0.4187	-0.0329	0.1884	0.2303	0.3769	0.4926	0.2692	0.6124	0.5041	0.491
42	0.687308	**8**	0.0799	-1.4432	-0.6069	-0.4626	0.0041	0.1194	-0.5085	0.5273	0.2870	0.283

Table S6. The table below shows the difference for any given ring-current between one oxidation state of **4-4-3CG** and the next.

Ring 1	Ring 2	Ring 3	Ring 4	Ring 5	Ring 6	Ring 7	Ring 8	Ring 9	Ring 10
-0.3388	-1.4103	-0.7953	-0.7029	-0.3728	-0.3732	-0.7777	-0.0851	-0.2171	-0.2084
-0.3389	-1.4104	-0.7954	-0.6830	-0.3728	-0.3732	-0.7776	-0.0852	-0.2170	-0.2083
0.1232	0.2679	0.0314	-0.1209	-0.0821	-0.0846	-0.0088	-0.0471	-0.0153	-0.0370
0.1232	0.2679	0.0313	-0.1209	-0.0821	-0.0846	-0.0089	-0.0471	-0.0154	-0.0371
0.0000	0.0000	0.0000	0.0000	0.0000	0.0000	0.0000	0.0000	0.0000	0.0000
0.0000	0.0000	0.0000	0.0000	0.0000	0.0000	0.0000	0.0000	0.0000	0.0000
0.0000	0.0000	0.0000	0.0000	0.0000	0.0000	0.0000	0.0000	0.0000	0.0000
0.0000	0.0000	0.0000	0.0000	0.0000	0.0000	0.0000	0.0000	0.0000	0.0000
0.0000	0.0000	0.0000	0.0000	0.0000	0.0000	0.0000	0.0000	0.0000	0.0000
0.0000	0.0000	0.0000	0.0000	0.0000	0.0000	0.0000	0.0000	0.0000	0.0000
0.0000	0.0000	0.0000	0.0000	0.0000	0.0000	0.0000	0.0000	0.0000	0.0000
0.0000	0.0000	0.0000	0.0000	0.0000	0.0000	0.0000	0.0000	0.0000	0.0000
-0.1232	-0.2679	-0.0313	0.1209	0.0821	0.0846	0.0089	0.0471	0.0154	0.0371
-0.1232	-0.2679	-0.0314	0.1209	0.0821	0.0846	0.0088	0.0471	0.0153	0.0370
0.3389	1.4104	0.7954	0.6930	0.3728	0.3732	0.7776	0.0852	0.2170	0.2083
0.3388	1.4103	0.7953	0.6929	0.3728	0.3732	0.7777	0.0851	0.2171	0.2084

The diagrams below show SYSMO[19] *ipso*-centric pseudo-π[20] calculations performed on the tetracation and tetraanion of **4-4-3CG**.

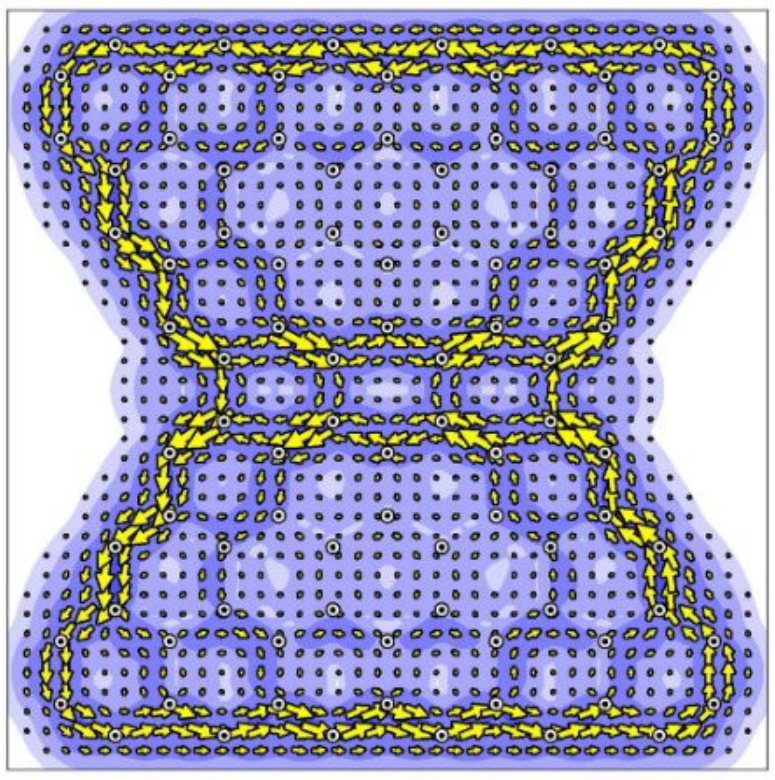

Tetracation

of 4-4-3CG

Figure S8.

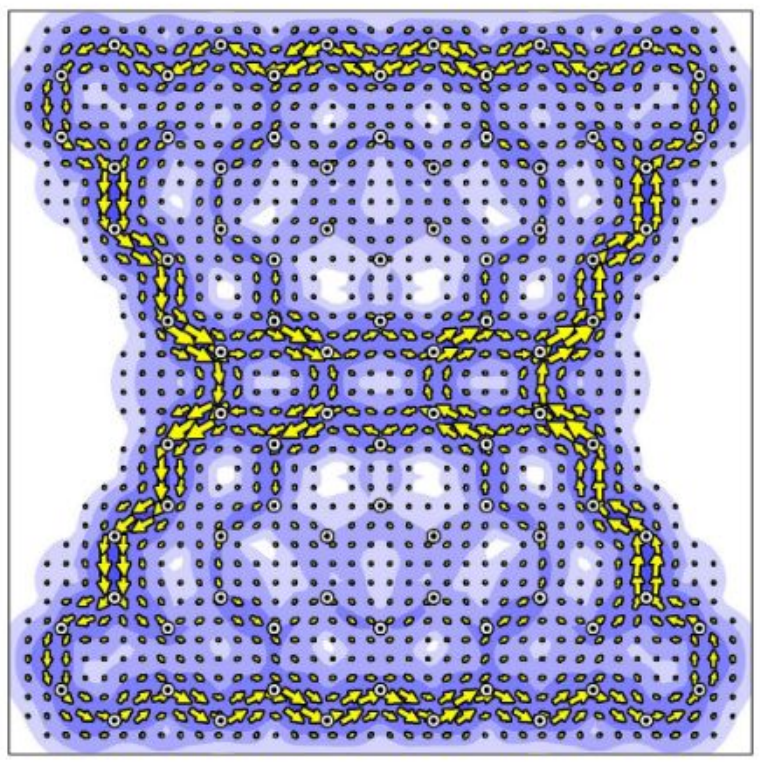

Tetraanion

of 4-4-3CG

Figure S9.

Notes

1. As with earlier goblets, diamagnetic ring-currents are predicted on the periphery of each goblet.
2. There is little current in the central anthracenic moiety.

5-5-2CG

The diagram below depicts the bond-current map of neutral **5-5-2CG** calculated by means of the Hückel–London approach.[11–15]

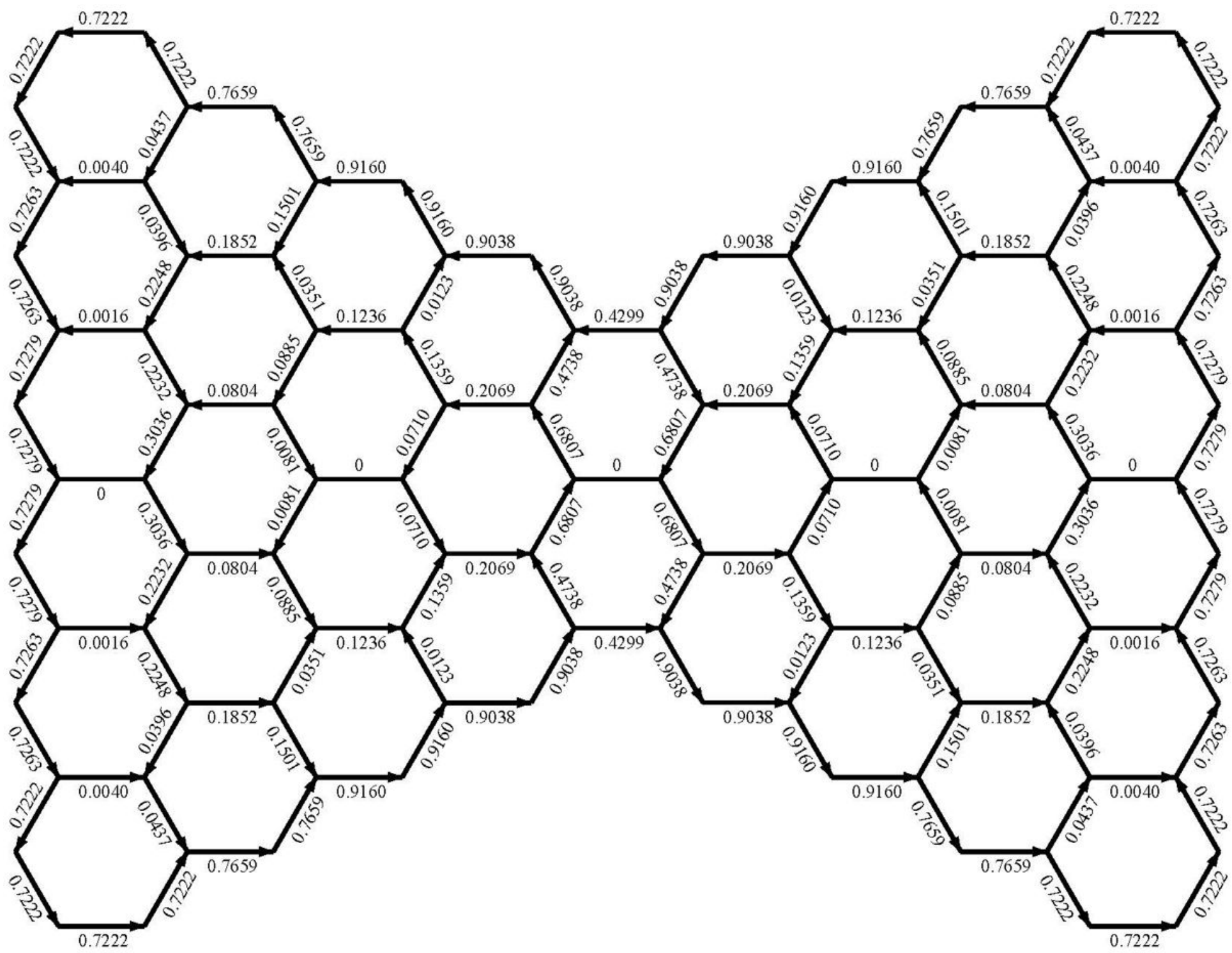

Figure S10.

Table S7. The table below displays ring-current intensities for various oxidation states of **5-5-2CG** and their associated eigenvalues.

Orbital	Eigenvalue	Charge	Ring 1	Ring 2	Ring 3	Ring 4	Ring 5	Ring 6
58	-0.5572	**-10**	-	-4.0849	-2.9724	-1.4694	-3.8942	-0.1315
		**-9**	-	-1.6252	-1.2001	-0.4386	-1.5130	0.2463
57	-0.1929	**-8**	0.9327	0.8345	0.5720	0.5923	0.8682	0.6242
		**-7**	0.6814	0.8691	0.8414	0.7541	0.9539	0.6950
56	0.0000	**-6**	0.4299	0.9038	1.1106	0.9160	1.0397	0.7659
		**-5**	0.4299	0.9038	1.1106	0.9160	1.0397	0.7659
55	0.0000	**-4**	0.4299	0.9038	1.1106	0.9160	1.0397	0.7659
		**-3**	0.4299	0.9038	1.1106	0.9160	1.0397	0.7659
54	0.0000	**-2**	0.4299	0.9038	1.1106	0.9160	1.0397	0.7659
		**-1**	0.4299	0.9038	1.1106	0.9160	1.0397	0.7659
53	0.0000	**0**	0.4299	0.9038	1.1106	0.9160	1.0397	0.7659
		**1**	0.4299	0.9038	1.1106	0.9160	1.0397	0.7659
52	0.0000	**2**	0.4299	0.9038	1.1106	0.9160	1.0397	0.7659
		**3**	0.4299	0.9038	1.1106	0.9160	1.0397	0.7659
51	0.0000	**4**	0.4299	0.9038	1.1106	0.9160	1.0397	0.7659
		**5**	0.4299	0.9038	1.1106	0.9160	1.0397	0.7659
50	0.1929	**6**	0.4299	0.9038	1.1106	0.9160	1.0397	0.7659
		**7**	0.6814	0.8691	0.8414	0.7541	0.9539	0.6950
49	0.5572	**8**	0.9327	0.8345	0.5720	0.5923	0.8682	0.6242
		**9**	-	-1.6252	-1.2001	-0.4386	-1.5130	0.2463
48	0.6549	**10**	-	-4.0849	-2.9724	-1.4694	-3.8942	-0.1315

Ring 7	Ring 8	Ring 9	Ring 10	Ring 11
-2.2849	-1.9199	0.3512	-0.1101	-0.5970
-0.7102	-0.5613	0.5233	0.2747	0.0397
0.8646	0.7975	0.6954	0.6595	0.6764
0.9078	0.9144	0.7088	0.6929	0.7021
0.9511	1.0315	0.7222	0.7263	0.7279
0.9511	1.0315	0.7222	0.7263	0.7279
0.9511	1.0315	0.7222	0.7263	0.7279
0.9511	1.0315	0.7222	0.7263	0.7279
0.9511	1.0315	0.7222	0.7263	0.7279
0.9511	1.0315	0.7222	0.7263	0.7279
0.9511	1.0315	0.7222	0.7263	0.7279
0.9511	1.0315	0.7222	0.7263	0.7279
0.9511	1.0315	0.7222	0.7263	0.7279
0.9511	1.0315	0.7222	0.7263	0.7279
0.9511	1.0315	0.7222	0.7263	0.7279
0.9511	1.0315	0.7222	0.7263	0.7279
0.9511	1.0315	0.7222	0.7263	0.7279
0.9078	0.9144	0.7088	0.6929	0.7021
0.8646	0.7975	0.6954	0.6595	0.6764
-0.7102	-0.5613	0.5233	0.2747	0.0397
-2.2849	-1.9199	0.3512	-0.1101	-0.5970

Table S8. The table below shows the difference for any given ring-current between one oxidation state of **5-5-2CG** and the next.

Ring 1	Ring 2	Ring 3	Ring 4	Ring 5	Ring 6	Ring 7	Ring 8	Ring 9	Ring 10	Ring 11
-2.2152	-2.4597	-1.7723	-1.0308	-2.3812	-0.3778	-1.5747	-1.3586	-0.1721	-0.3848	-0.6367
-2.2151	-2.4597	-1.7721	-1.0309	-2.3812	-0.3779	-1.5748	-1.3588	-0.1721	-0.3848	-0.6367
0.2513	-0.0346	-0.2694	-0.1618	-0.0857	-0.0708	-0.0432	-0.1169	-0.0134	-0.0334	-0.0257
0.2515	-0.0347	-0.2692	-0.1619	-0.0858	-0.0709	-0.0433	-0.1171	-0.0134	-0.0334	-0.0258
0.0000	0.0000	0.0000	0.0000	0.0000	0.0000	0.0000	0.0000	0.0000	0.0000	0.0000
0.0000	0.0000	0.0000	0.0000	0.0000	0.0000	0.0000	0.0000	0.0000	0.0000	0.0000
0.0000	0.0000	0.0000	0.0000	0.0000	0.0000	0.0000	0.0000	0.0000	0.0000	0.0000
0.0000	0.0000	0.0000	0.0000	0.0000	0.0000	0.0000	0.0000	0.0000	0.0000	0.0000
0.0000	0.0000	0.0000	0.0000	0.0000	0.0000	0.0000	0.0000	0.0000	0.0000	0.0000
0.0000	0.0000	0.0000	0.0000	0.0000	0.0000	0.0000	0.0000	0.0000	0.0000	0.0000
0.0000	0.0000	0.0000	0.0000	0.0000	0.0000	0.0000	0.0000	0.0000	0.0000	0.0000
0.0000	0.0000	0.0000	0.0000	0.0000	0.0000	0.0000	0.0000	0.0000	0.0000	0.0000
0.0000	0.0000	0.0000	0.0000	0.0000	0.0000	0.0000	0.0000	0.0000	0.0000	0.0000
0.0000	0.0000	0.0000	0.0000	0.0000	0.0000	0.0000	0.0000	0.0000	0.0000	0.0000
0.0000	0.0000	0.0000	0.0000	0.0000	0.0000	0.0000	0.0000	0.0000	0.0000	0.0000
0.0000	0.0000	0.0000	0.0000	0.0000	0.0000	0.0000	0.0000	0.0000	0.0000	0.0000
-0.2515	0.0347	0.2692	0.1619	0.0858	0.0709	0.0433	0.1171	0.0134	0.0334	0.0258
-0.2513	0.0346	0.2694	0.1618	0.0857	0.0708	0.0432	0.1169	0.0134	0.0334	0.0257
2.2151	2.4597	1.7721	1.0309	2.3812	0.3779	1.5748	1.3588	0.1721	0.3848	0.6367
2.2152	2.4597	1.7723	1.0308	2.3812	0.3778	1.5747	1.3586	0.1721	0.3848	0.6367

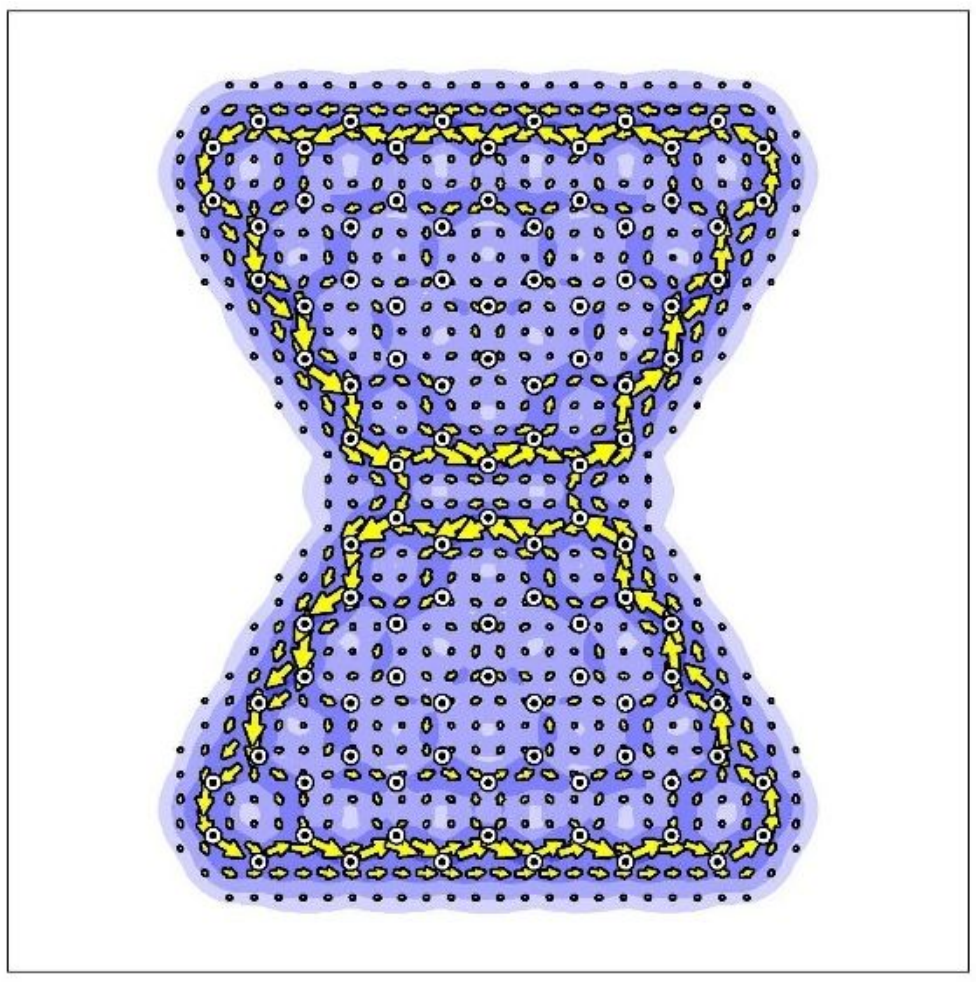

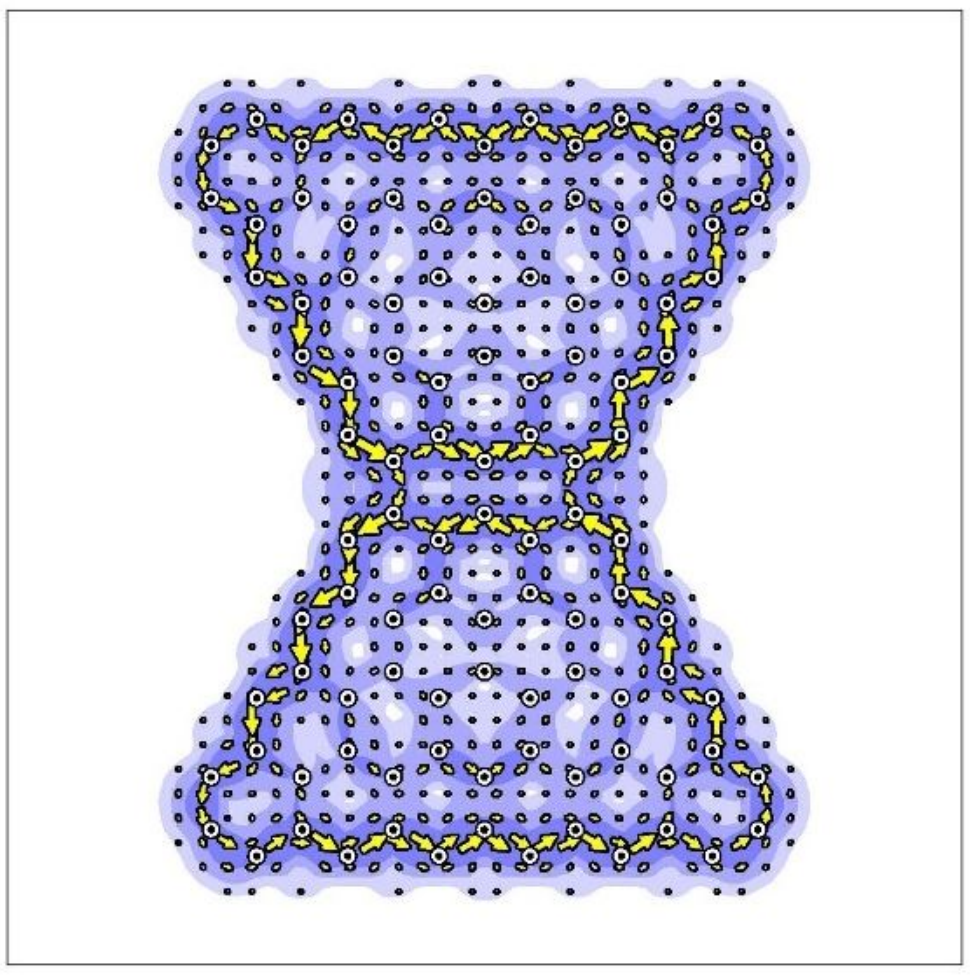

Hexacation

of 5-5-2CG

Figure S11.

Hexaanion

of 5-5-2CG

Figure S12.

The diagrams above show SYSMO[19] *ipso*-centric pseudo-π[20] calculations performed on the Hexacation and Hexaanion of **5-5-2CG**.

5-5-3CG

The diagram below depicts the bond-current map of neutral **5-5-3CG** calculated by means of the Hückel–London approach.[11–15]

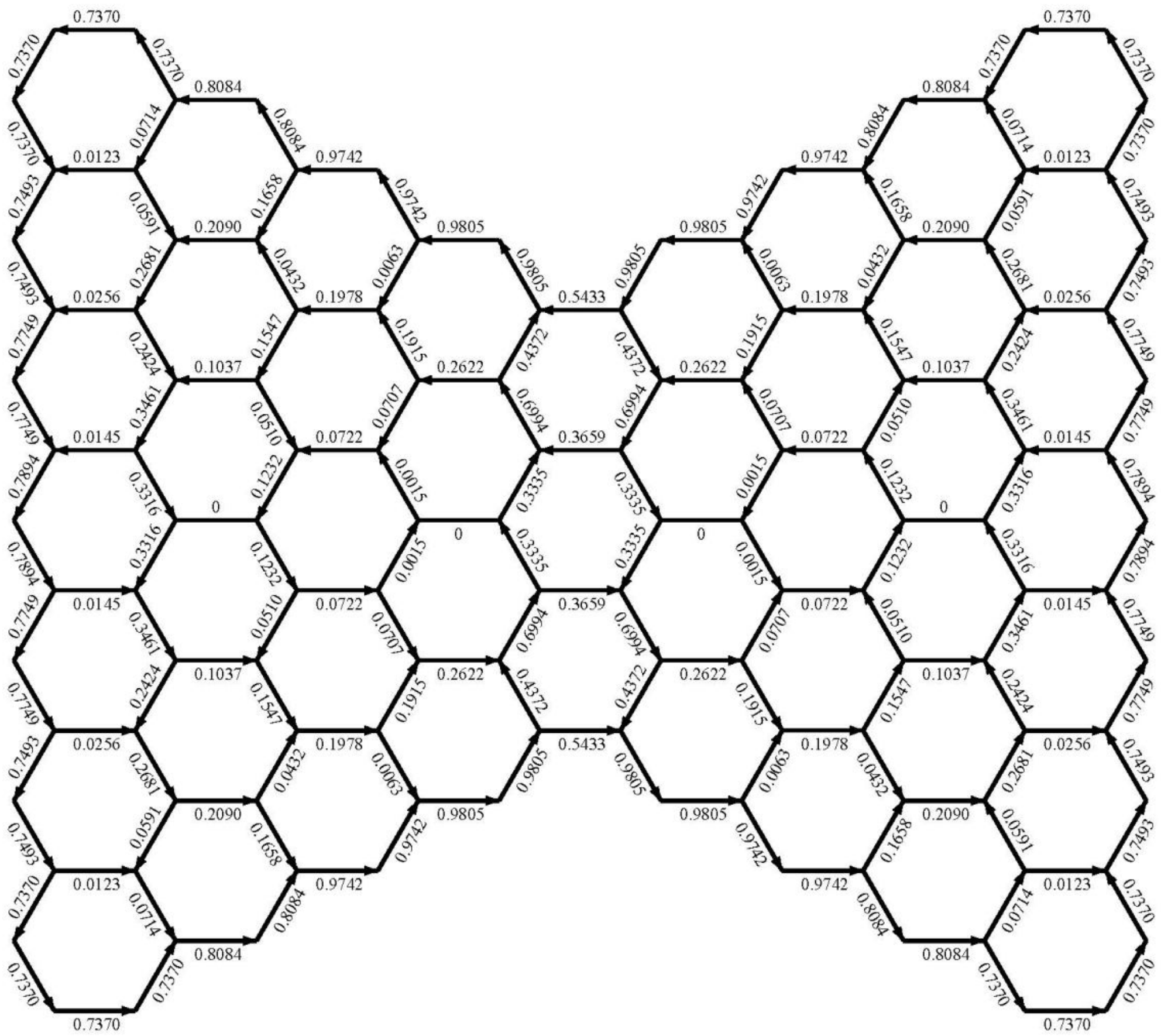

Figure S13.

Table S9. The table below displays ring-current intensities for various oxidation states of **5-5-3CG** and their associated eigenvalues.

Orbital	Eigenvalue	Charge	Ring 1	Ring 2	Ring 3	Ring 4	Ring 5	Ring 6	Ring 7
68	-0.3893	**-10**	0.0062	-1.7795	-0.8566	-0.7164	-0.1925	-0.1167	-1.0340
		**-9**	0.4027	-0.1519	0.1067	0.1482	0.3094	0.4345	0.1020
67	-0.1016	**-8**	0.7992	1.4756	1.0700	1.0128	0.8114	0.9859	1.2381
		**-7**	0.6713	1.1925	1.0253	1.1278	0.8928	1.0789	1.2412
66	0.0000	**-6**	0.5433	0.9092	0.9805	1.2427	0.9742	1.1721	1.2442
		**-5**	0.5433	0.9092	0.9805	1.2427	0.9742	1.1721	1.2442
65	0.0000	**-4**	0.5433	0.9092	0.9805	1.2427	0.9742	1.1721	1.2442
		**-3**	0.5433	0.9092	0.9805	1.2427	0.9742	1.1721	1.2442
64	0.0000	**-2**	0.5433	0.9092	0.9805	1.2427	0.9742	1.1721	1.2442
		**-1**	0.5433	0.9092	0.9805	1.2427	0.9742	1.1721	1.2442
63	0.0000	**0**	0.5433	0.9092	0.9805	1.2427	0.9742	1.1721	1.2442
		**1**	0.5433	0.9092	0.9805	1.2427	0.9742	1.1721	1.2442
62	0.0000	**2**	0.5433	0.9092	0.9805	1.2427	0.9742	1.1721	1.2442
		**3**	0.5433	0.9092	0.9805	1.2427	0.9742	1.1721	1.2442
61	0.0000	**4**	0.5433	0.9092	0.9805	1.2427	0.9742	1.1721	1.2442
		**5**	0.5433	0.9092	0.9805	1.2427	0.9742	1.1721	1.2442
60	0.1016	**6**	0.5433	0.9092	0.9805	1.2427	0.9742	1.1721	1.2442
		**7**	0.6713	1.1925	1.0253	1.1278	0.8928	1.0789	1.2412
59	0.5572	**8**	0.7992	1.4756	1.0700	1.0128	0.8114	0.9859	1.2381
		**9**	0.4027	-0.1519	0.1067	0.1482	0.3094	0.4345	0.1020
58	0.6549	**10**	0.0062	-1.7795	-0.8566	-0.7164	-0.1925	-0.1167	-1.0340

Ring 8	Ring 9	Ring 10	Ring 11	Ring 12	Ring 13	Ring 14
0.4374	0.0368	0.0381	0.6273	0.4448	0.4346	0.2545
0.5640	0.5069	0.5174	0.6684	0.5782	0.5780	0.5117
0.6906	0.9771	0.9969	0.7096	0.7116	0.7215	0.7690
0.7495	0.9972	1.0589	0.7233	0.7305	0.7482	0.7792
0.8084	1.0174	1.1211	0.7370	0.7493	0.7749	0.7894
0.8084	1.0174	1.1211	0.7370	0.7493	0.7749	0.7894
0.8084	1.0174	1.1211	0.7370	0.7493	0.7749	0.7894
0.8084	1.0174	1.1211	0.7370	0.7493	0.7749	0.7894
0.8084	1.0174	1.1211	0.7370	0.7493	0.7749	0.7894
0.8084	1.0174	1.1211	0.7370	0.7493	0.7749	0.7894
0.8084	1.0174	1.1211	0.7370	0.7493	0.7749	0.7894
0.8084	1.0174	1.1211	0.7370	0.7493	0.7749	0.7894
0.8084	1.0174	1.1211	0.7370	0.7493	0.7749	0.7894
0.8084	1.0174	1.1211	0.7370	0.7493	0.7749	0.7894
0.8084	1.0174	1.1211	0.7370	0.7493	0.7749	0.7894
0.8084	1.0174	1.1211	0.7370	0.7493	0.7749	0.7894
0.8084	1.0174	1.1211	0.7370	0.7493	0.7749	0.7894
0.7495	0.9972	1.0589	0.7233	0.7305	0.7482	0.7792

0.6906	0.9771	0.9969	0.7096	0.7116	0.7215	0.7690
0.5640	0.5069	0.5174	0.6684	0.5782	0.5780	0.5117
0.4374	0.0368	0.0381	0.6273	0.4448	0.4346	0.2545

Table S10. The table below shows the difference for any given ring-current between one oxidation state of **5-5-3CG** and the next.

Ring 1	Ring 2	Ring 3	Ring 4	Ring 5	Ring 6	Ring 7
-0.3965	-1.6276	-0.9633	-0.8646	-0.5019	-0.5512	-1.1360
-0.3965	-1.6275	-0.9633	-0.8646	-0.5020	-0.5514	-1.1361
0.1279	0.2831	0.0447	-0.1150	-0.0814	-0.0930	-0.0031
0.1280	0.2833	0.0448	-0.1149	-0.0814	-0.0932	-0.0030
0.0000	0.0000	0.0000	0.0000	0.0000	0.0000	0.0000
0.0000	0.0000	0.0000	0.0000	0.0000	0.0000	0.0000
0.0000	0.0000	0.0000	0.0000	0.0000	0.0000	0.0000
0.0000	0.0000	0.0000	0.0000	0.0000	0.0000	0.0000
0.0000	0.0000	0.0000	0.0000	0.0000	0.0000	0.0000
0.0000	0.0000	0.0000	0.0000	0.0000	0.0000	0.0000
0.0000	0.0000	0.0000	0.0000	0.0000	0.0000	0.0000
0.0000	0.0000	0.0000	0.0000	0.0000	0.0000	0.0000
0.0000	0.0000	0.0000	0.0000	0.0000	0.0000	0.0000
0.0000	0.0000	0.0000	0.0000	0.0000	0.0000	0.0000
0.0000	0.0000	0.0000	0.0000	0.0000	0.0000	0.0000
0.0000	0.0000	0.0000	0.0000	0.0000	0.0000	0.0000
-0.1280	-0.2833	-0.0448	0.1149	0.0814	0.0932	0.0030
-0.1279	-0.2831	-0.0447	0.1150	0.0814	0.0930	0.0031
0.3965	1.6275	0.9633	0.8646	0.5020	0.5514	1.1361
0.3965	1.6276	0.9633	0.8646	0.5019	0.5512	1.1360

Ring 8	Ring 9	Ring 10	Ring 11	Ring 12	Ring 13	Ring 14
-0.1266	-0.4701	-0.4793	-0.0411	-0.1334	-0.1434	-0.2572
-0.1266	-0.4702	-0.4795	-0.0412	-0.1334	-0.1435	-0.2573
-0.0589	-0.0201	-0.0620	-0.0137	-0.0189	-0.0267	-0.0102
-0.0589	-0.0202	-0.0622	-0.0137	-0.0188	-0.0267	-0.0102
0.0000	0.0000	0.0000	0.0000	0.0000	0.0000	0.0000
0.0000	0.0000	0.0000	0.0000	0.0000	0.0000	0.0000
0.0000	0.0000	0.0000	0.0000	0.0000	0.0000	0.0000
0.0000	0.0000	0.0000	0.0000	0.0000	0.0000	0.0000
0.0000	0.0000	0.0000	0.0000	0.0000	0.0000	0.0000
0.0000	0.0000	0.0000	0.0000	0.0000	0.0000	0.0000
0.0000	0.0000	0.0000	0.0000	0.0000	0.0000	0.0000
0.0000	0.0000	0.0000	0.0000	0.0000	0.0000	0.0000
0.0000	0.0000	0.0000	0.0000	0.0000	0.0000	0.0000
0.0000	0.0000	0.0000	0.0000	0.0000	0.0000	0.0000
0.0000	0.0000	0.0000	0.0000	0.0000	0.0000	0.0000
0.0000	0.0000	0.0000	0.0000	0.0000	0.0000	0.0000
0.0589	0.0202	0.0622	0.0137	0.0188	0.0267	0.0102

0.0589	0.0201	0.0620	0.0137	0.0189	0.0267	0.0102
0.1266	0.4702	0.4795	0.0412	0.1334	0.1435	0.2573
0.1266	0.4701	0.4793	0.0411	0.1334	0.1434	0.2572

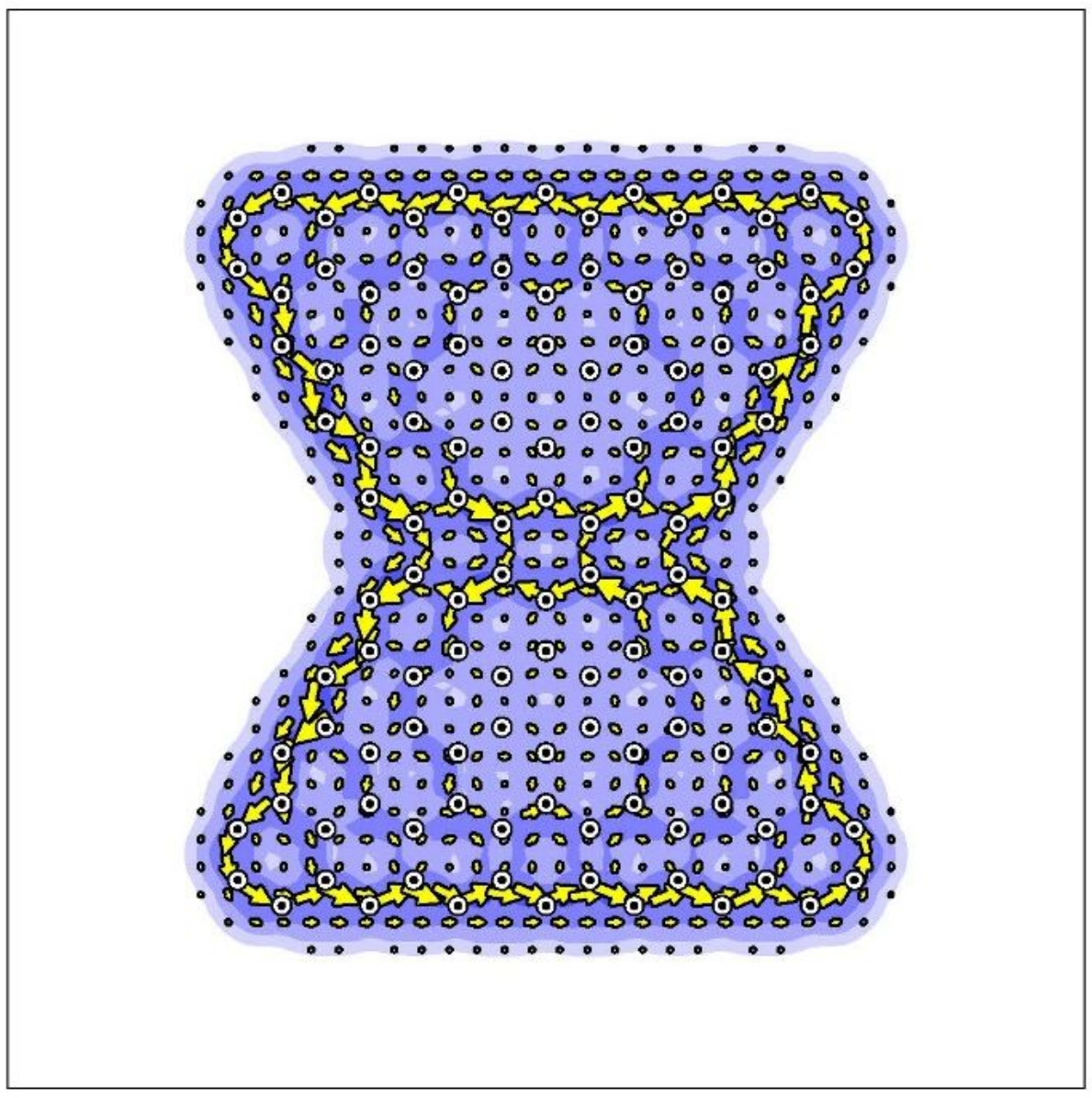

Hexacation of 5-5-3CG

Figure S14.

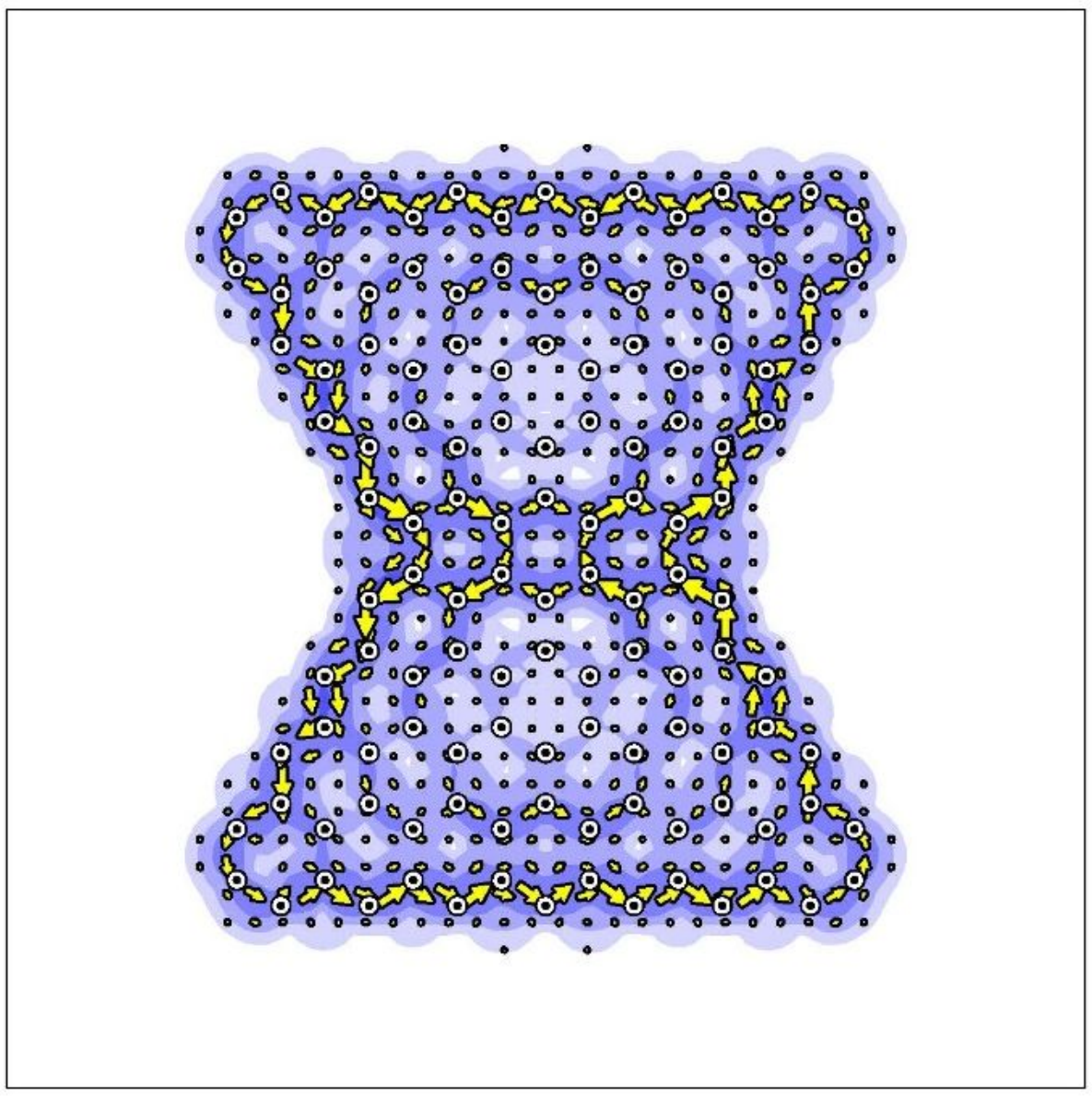

Hexaanion of 5-5-3CG

Figure S15.

The diagrams above show SYSMO[19] *ipso*-centric pseudo-π[20] calculations performed on the Hexacation and Hexaanion of **5-5-3CG**.

5-4-2CG

The diagram below depicts the bond-current map of neutral **5-4-2CG** calculated by means of the Hückel–London approach.[11–15]

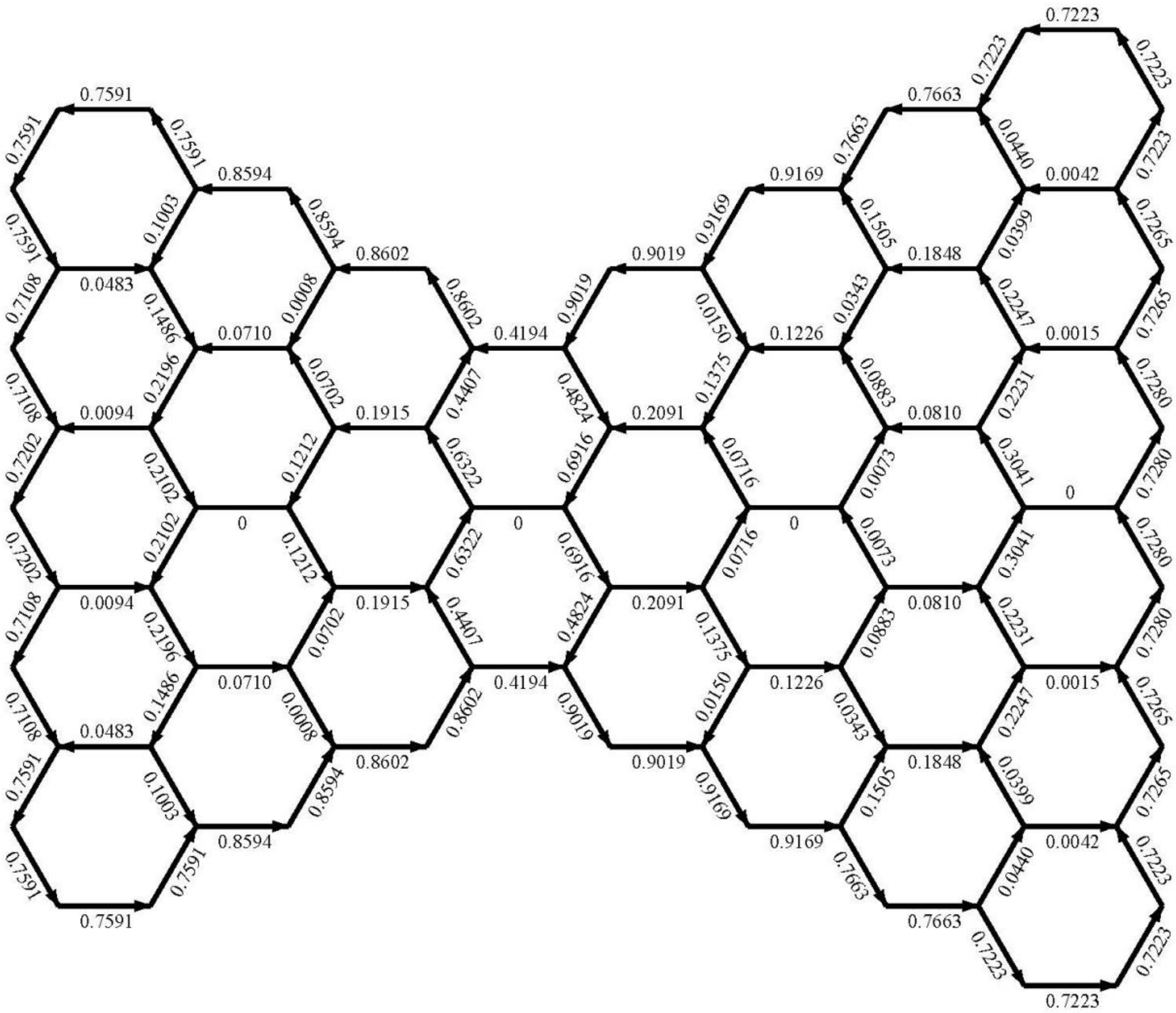

Figure S16.

Table S11. The table below displays ring-current intensities for various oxidation states of **5-4-2CG** and their associated eigenvalues.

Orbital	Eigenvalue	Charge	Ring 1	Ring 2	Ring 3	Ring 4	Ring 5	Ring 6	Ring 7	Ring 8
		**-10**	-0.5050	-1.8135	- 1.9753	-2.0489	- 3.0073	-1.0086	- 2.7477	-3.6688
50	-0.5689	**-9**	-2.9433	-3.9754	- 2.7972	-1.5044	- 4.2236	-0.2260	- 2.6690	-2.2105
		**-8**	-1.0172	-1.5787	- 1.1272	-0.4635	- 1.6840	0.1961	- 0.9051	-0.7121
49	-0.1969	**-7**	0.9088	0.8179	0.5426	0.5775	0.8556	0.6183	0.8588	0.7862
		**-6**	0.6641	0.8599	0.8268	0.7471	0.9475	0.6923	2.3251	0.9091
48	0.0000	**-5**	0.4194	0.9019	1.1110	0.9169	1.0394	0.7663	0.9503	0.9507
		**-4**	0.4194	0.9019	1.1110	0.9169	1.0394	0.7663	0.9503	0.9507
47	0.0000	**-3**	0.4194	0.9019	1.1110	0.9169	1.0394	0.7663	0.9503	0.9507
		**-2**	0.4194	0.9019	1.1110	0.9169	1.0394	0.7663	0.9503	0.9507
46	0.0000	**-1**	0.4194	0.9019	1.1110	0.9169	1.0394	0.7663	0.9503	0.9507
		**0**	0.4194	0.9019	1.1110	0.9169	1.0394	0.7663	0.9503	0.9507
45	0.0000	**1**	0.4194	0.9019	1.1110	0.9169	1.0394	0.7663	0.9503	0.9507
		**2**	0.4194	0.9019	1.1110	0.9169	1.0394	0.7663	0.9503	0.9507

44	0.0000	**3**	0.4194	0.9019	1.1110	0.9169	1.0394	0.7663	0.9503	0.9507
		**4**	0.4194	0.9019	1.1110	0.9169	1.0394	0.7663	0.9503	0.9507
43	0.1969	**5**	0.4194	0.9019	1.1110	0.9169	1.0394	0.7663	0.9503	0.9507
		**6**	0.6641	0.8599	0.8268	0.7471	0.9475	0.6923	0.9049	0.9091
42	0.5689	**7**	0.9088	0.8179	0.5426	0.5775	0.8556	0.6183	0.8588	0.7862
		**8**	-1.0172	-1.5787	-1.1272	-0.4635	-1.6840	0.1961	-0.9051	-0.7121
41	0.5572	**9**	-2.9433	-3.9754	-2.7972	-1.5044	-4.2236	-0.2260	-2.6690	-2.2105
		**10**	-0.5050	-1.8135	-1.9753	-2.0489	-3.0073	-1.0086	-2.7477	-3.6688

Ring 9	Ring 10	Ring 11	Ring 12	Ring 13	Ring 14	Ring 15	Ring 16	Ring 17	Ring 18
-0.1444	-1.1207	-1.1335	0.3820	0.0718	0.0678	0.2487	0.4918	0.3611	0.4062
0.2765	-0.2330	-0.0795	-2.7329	-1.9556	-0.6420	-1.7257	0.2949	-0.3356	-0.1757
0.4854	0.2167	-0.0605	-0.9726	-0.6969	-0.0296	-0.4680	0.4740	0.1640	0.2125
0.6942	0.6565	0.6736	0.7877	0.5617	0.5828	0.7897	0.6532	0.6636	0.6026
0.7083	0.6915	0.7008	0.8239	0.8066	0.7211	0.8600	0.7061	0.6872	0.6614
0.7223	0.7265	0.7280	0.8602	1.0516	0.8594	0.9304	0.7591	0.7108	0.7202
0.7223	0.7265	0.7280	0.8602	1.0516	0.8594	0.9304	0.7591	0.7108	0.7202
0.7223	0.7265	0.7280	0.8602	1.0516	0.8594	0.9304	0.7591	0.7108	0.7202
0.7223	0.7265	0.7280	0.8602	1.0516	0.8594	0.9304	0.7591	0.7108	0.7202
0.7223	0.7265	0.7280	0.8602	1.0516	0.8594	0.9304	0.7591	0.7108	0.7202
0.7223	0.7265	0.7280	0.8602	1.0516	0.8594	0.9304	0.7591	0.7108	0.7202
0.7223	0.7265	0.7280	0.8602	1.0516	0.8594	0.9304	0.7591	0.7108	0.7202
0.7223	0.7265	0.7280	0.8602	1.0516	0.8594	0.9304	0.7591	0.7108	0.7202
0.7223	0.7265	0.7280	0.8602	1.0516	0.8594	0.9304	0.7591	0.7108	0.7202
0.7223	0.7265	0.7280	0.8602	1.0516	0.8594	0.9304	0.7591	0.7108	0.7202
0.7223	0.7265	0.7280	0.8602	1.0516	0.8594	0.9304	0.7591	0.7108	0.7202
0.7083	0.6915	0.7008	0.8239	0.8066	0.7211	0.8600	0.7061	0.6872	0.6614
0.6942	0.6565	0.6736	0.7877	0.5617	0.5828	0.7897	0.6532	0.6636	0.6026
0.4854	0.2167	-0.0605	-0.9726	-0.6969	-0.0296	-0.4680	0.4740	0.1640	0.2125
0.2765	-0.2330	-0.0795	-2.7329	-1.9556	-0.6420	-1.7257	0.2949	-0.3356	-0.1757
-0.1444	-1.1207	-1.1335	-0.3820	0.0718	0.0678	0.2487	0.4918	0.3611	0.4062

Table S12. The table below displays ring-current intensities for various oxidation states of **5-4-2CG** and their associated eigenvalues.

Ring 1	Ring 2	Ring 3	Ring 4	Ring 5	Ring 6	Ring 7	Ring 8	Ring 9
2.4383	2.1619	0.8219	-0.5445	1.2163	-0.7826	-0.0787	-1.4583	-0.4209
-1.9261	-2.3967	-1.6700	-1.0409	-2.5396	-0.4221	-1.7639	-1.4984	-0.2089
-1.9260	-2.3966	-1.6698	-1.0410	-2.5396	-0.4222	-1.7639	-1.4983	-0.2088
0.2447	-0.0420	-0.2842	-0.1696	-0.0919	-0.0740	-1.4663	-0.1229	-0.0141

0.2447	-0.0420	-0.2842	-0.1698	-0.0919	-0.0740	1.3748	-0.0416	-0.0140
0.0000	0.0000	0.0000	0.0000	0.0000	0.0000	0.0000	0.0000	0.0000
0.0000	0.0000	0.0000	0.0000	0.0000	0.0000	0.0000	0.0000	0.0000
0.0000	0.0000	0.0000	0.0000	0.0000	0.0000	0.0000	0.0000	0.0000
0.0000	0.0000	0.0000	0.0000	0.0000	0.0000	0.0000	0.0000	0.0000
0.0000	0.0000	0.0000	0.0000	0.0000	0.0000	0.0000	0.0000	0.0000
0.0000	0.0000	0.0000	0.0000	0.0000	0.0000	0.0000	0.0000	0.0000
0.0000	0.0000	0.0000	0.0000	0.0000	0.0000	0.0000	0.0000	0.0000
0.0000	0.0000	0.0000	0.0000	0.0000	0.0000	0.0000	0.0000	0.0000
0.0000	0.0000	0.0000	0.0000	0.0000	0.0000	0.0000	0.0000	0.0000
0.0000	0.0000	0.0000	0.0000	0.0000	0.0000	0.0000	0.0000	0.0000
-0.2447	0.0420	0.2842	0.1698	0.0919	0.0740	0.0454	0.0416	0.0140
-0.2447	0.0420	0.2842	0.1696	0.0919	0.0740	0.0461	0.1229	0.0141
1.9260	2.3966	1.6698	1.0410	2.5396	0.4222	1.7639	1.4983	0.2088
1.9261	2.3967	1.6700	1.0409	2.5396	0.4221	1.7639	1.4984	0.2089
-2.4383	-2.1619	-0.8219	0.5445	-1.2163	0.7826	0.0787	1.4583	0.4209

Ring 10	Ring 11	Ring 12	Ring 13	Ring 14	Ring 15	Ring 16	Ring 17	Ring 18
-0.8877	-1.0540	3.1149	2.0274	0.7098	1.9744	0.1969	0.6967	0.5819
-0.4497	-0.0190	-1.7603	-1.2587	-0.6124	-1.2577	-0.1791	-0.4996	-0.3882
-0.4398	-0.7341	-1.7603	-1.2586	-0.6124	-1.2577	-0.1792	-0.4996	-0.3901
-0.0350	-0.0272	-0.0362	-0.2449	-0.1383	-0.0703	-0.0529	-0.0236	-0.0588
-0.0350	-0.0272	-0.0363	-0.2450	-0.1383	-0.0704	-0.0530	-0.0236	-0.0588
0.0000	0.0000	0.0000	0.0000	0.0000	0.0000	0.0000	0.0000	0.0000
0.0000	0.0000	0.0000	0.0000	0.0000	0.0000	0.0000	0.0000	0.0000
0.0000	0.0000	0.0000	0.0000	0.0000	0.0000	0.0000	0.0000	0.0000
0.0000	0.0000	0.0000	0.0000	0.0000	0.0000	0.0000	0.0000	0.0000
0.0000	0.0000	0.0000	0.0000	0.0000	0.0000	0.0000	0.0000	0.0000
0.0000	0.0000	0.0000	0.0000	0.0000	0.0000	0.0000	0.0000	0.0000
0.0000	0.0000	0.0000	0.0000	0.0000	0.0000	0.0000	0.0000	0.0000
0.0000	0.0000	0.0000	0.0000	0.0000	0.0000	0.0000	0.0000	0.0000
0.0000	0.0000	0.0000	0.0000	0.0000	0.0000	0.0000	0.0000	0.0000
0.0000	0.0000	0.0000	0.0000	0.0000	0.0000	0.0000	0.0000	0.0000
0.0350	0.0272	0.0363	0.2450	0.1383	0.0704	0.0530	0.0236	0.0588
0.0350	0.0272	0.0362	0.2449	0.1383	0.0703	0.0529	0.0236	0.0588
0.4398	0.7341	1.7603	1.2586	0.6124	1.2577	0.1792	0.4996	0.3901
0.4497	0.0190	1.7603	1.2587	0.6124	1.2577	0.1791	0.4996	0.3882
0.8877	1.0540	-2.3509	-2.0274	-0.7098	-1.9744	-0.1969	-0.6967	-0.5819

The diagrams below show SYSMO[19] *ipso*-centric pseudo-π[20] calculations performed on the pentacation and pentaanion of **5-4-2CG**.

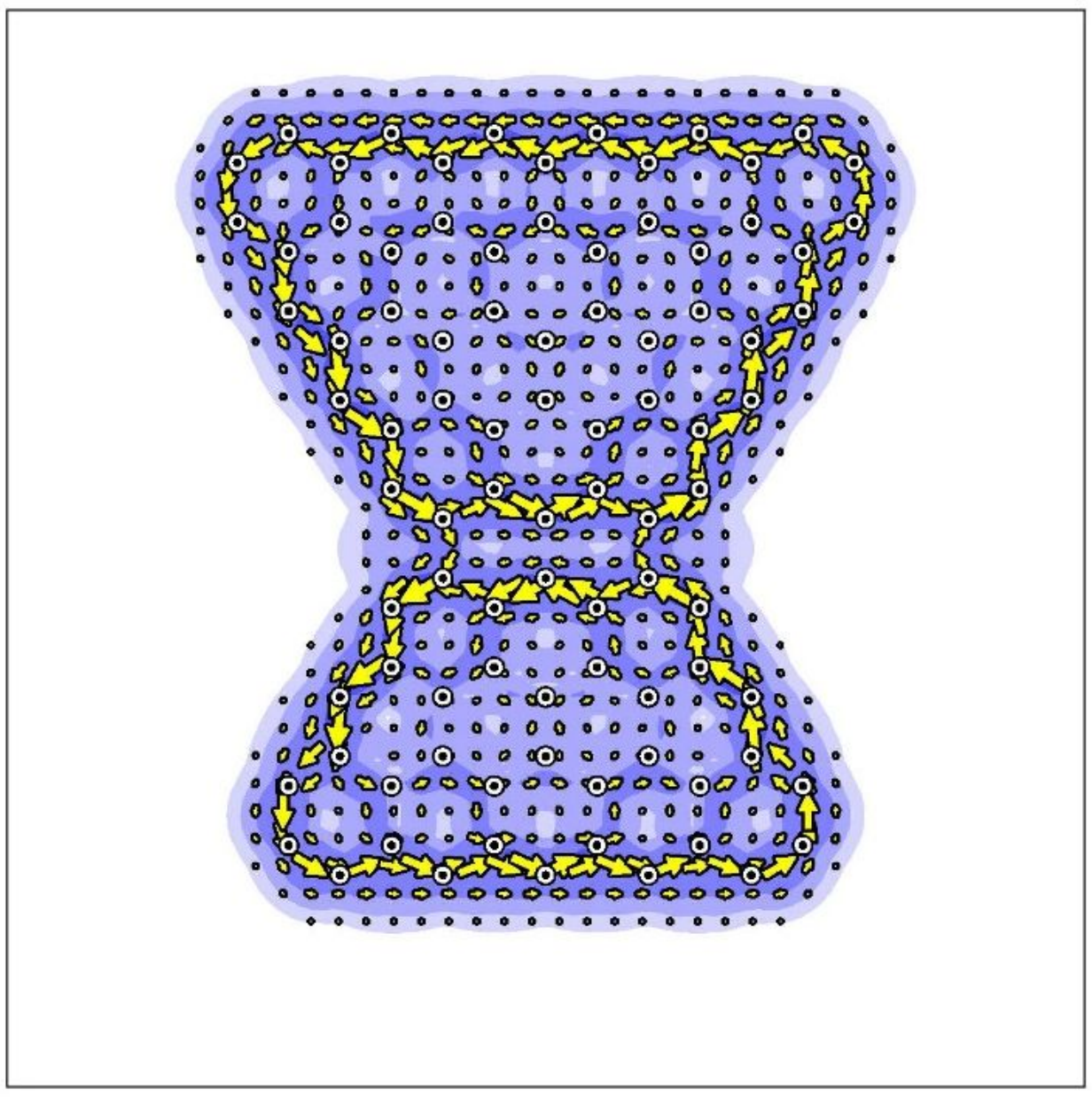

Pentacation of 5-4-2CG

Figure S17.

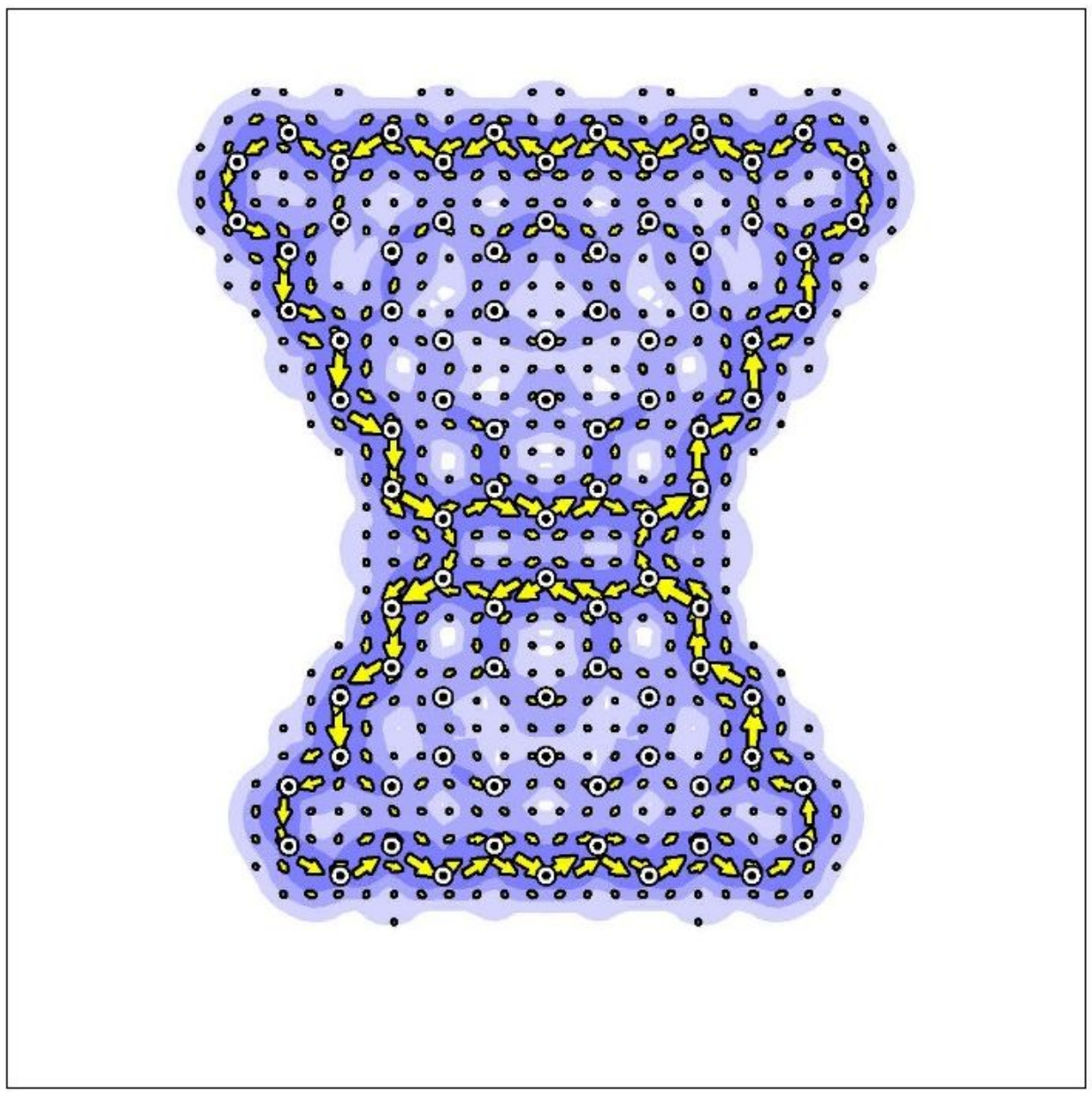

Pentaanion of 5-4-2CG

Figure S18.

The diagram below depicts the bond-current map of neutral **5-3-2CG** calculated by means of the Hückel–London approach.[11–15]

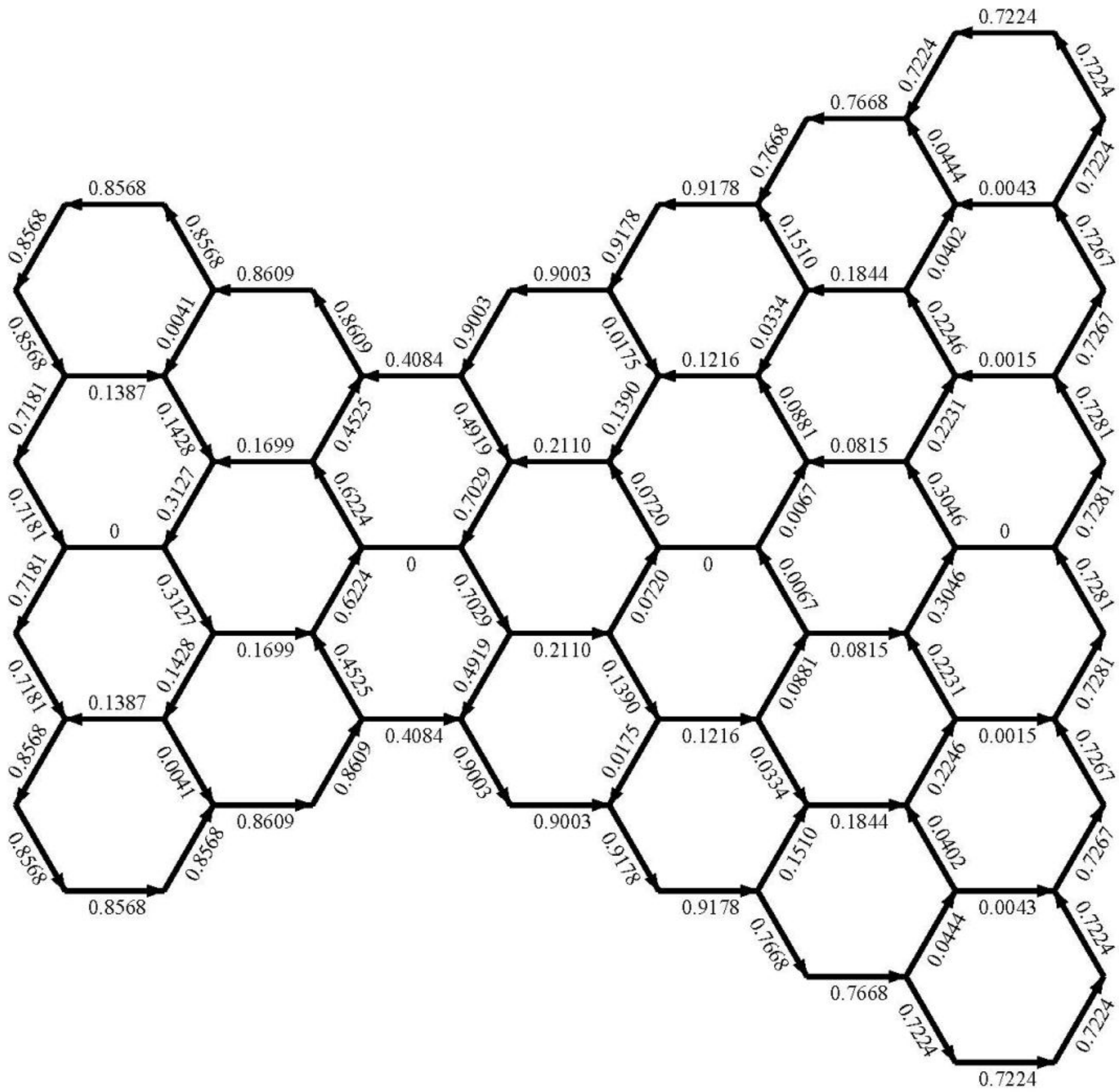

Figure S19.

Table S13. The table below displays ring-current intensities for various oxidation states of **5-3-2CG** and their associated eigenvalues.

Orbital	Eigenvalue	Charge	Ring 1	Ring 2	Ring 3	Ring 4	Ring 5	Ring 6	Ring 7
43	-0.5841	**-8**	-3.0228	-4.4349	-3.1970	-1.8215	-5.3107	-0.4743	-3.5854
		**-7**	-1.0706	-1.8171	-1.3417	-0.6293	-2.2338	0.0692	-1.3662
42	-0.2008	**-6**	0.8816	0.8007	0.5137	0.5629	0.8430	0.6126	0.8530
		**-5**	0.6450	0.8506	0.8125	0.7403	0.9413	0.6897	0.9021
41	0.0000	**-4**	0.4084	0.9003	1.1113	0.9178	1.0393	0.7668	0.9512
		**-3**	0.4084	0.9003	1.1113	0.9178	1.0393	0.7668	0.9512
40	0.0000	**-2**	0.4084	0.9003	1.1113	0.9178	1.0393	0.7668	0.9512
		**-1**	0.4084	0.9003	1.1113	0.9178	1.0393	0.7668	0.9512
39	0.0000	**0**	0.4084	0.9003	1.1113	0.9178	1.0393	0.7668	0.9512
		**1**	0.4084	0.9003	1.1113	0.9178	1.0393	0.7668	0.9512
38	0.0000	**2**	0.4084	0.9003	1.1113	0.9178	1.0393	0.7668	0.9512
		**3**	0.4084	0.9003	1.1113	0.9178	1.0393	0.7668	0.9512
37	0.2007	**4**	0.4084	0.9003	1.1113	0.9178	1.0393	0.7668	0.9512
		**5**	0.6450	0.8506	0.8125	0.7403	0.9413	0.6897	0.9021
36	0.5841	**6**	0.8816	0.8007	0.5137	0.5629	0.8430	0.6126	0.8530
		**7**	-1.0706	-1.8171	-1.3417	-0.6293	-2.2338	0.0692	-1.3662
35	0.6924	**8**	-3.0228	-4.4349	-3.1970	-1.8215	-5.3107	-0.4743	-3.5854

Ring 8	Ring 9	Ring 10	Ring 11	Ring 12	Ring 13	Ring 14	Ring 15
-3.0404	0.1212	-0.4876	-1.2448	-2.0568	-1.4271	-0.2412	-0.3310
-1.1325	0.4072	0.0829	-0.2869	-0.6624	-0.4537	0.1868	0.1354
0.7754	0.6931	0.6534	0.6709	0.7321	0.5197	0.6147	0.6019
0.9040	0.7077	0.6901	0.6995	0.7965	0.7752	0.7358	0.6600
1.0327	0.7224	0.7267	0.7281	0.8609	1.0308	0.8568	0.7181
1.0327	0.7224	0.7267	0.7281	0.8609	1.0308	0.8568	0.7181
1.0327	0.7224	0.7267	0.7281	0.8609	1.0308	0.8568	0.7181
1.0327	0.7224	0.7267	0.7281	0.8609	1.0308	0.8568	0.7181
1.0327	0.7224	0.7267	0.7281	0.8609	1.0308	0.8568	0.7181
1.0327	0.7224	0.7267	0.7281	0.8609	1.0308	0.8568	0.7181
1.0327	0.7224	0.7267	0.7281	0.8609	1.0308	0.8568	0.7181
1.0327	0.7224	0.7267	0.7281	0.8609	1.0308	0.8568	0.7181
1.0327	0.7224	0.7267	0.7281	0.8609	1.0308	0.8568	0.7181
0.9040	0.7077	0.6901	0.6995	0.7965	0.7752	0.7358	0.6600
0.7754	0.6931	0.6534	0.6709	0.7321	0.5197	0.6147	0.6019
-1.1325	0.4072	0.0829	-0.2869	-0.6624	-0.4537	0.1868	0.1354
-3.0404	0.1212	-0.4876	-1.2448	-2.0568	-1.4271	-0.2412	-0.3310

Table S14. The table below shows the difference for any given ring-current between one oxidation state of **5-3-2CG** and the next.

Ring 1	Ring 2	Ring 3	Ring 4	Ring 5	Ring 6	Ring 7
-1.9522	-2.6178	-1.8553	-1.1922	-3.0769	-0.5435	-2.2192
-1.9522	-2.6178	-1.8554	-1.1922	-3.0768	-0.5434	-2.2192
0.2366	-0.0499	-0.2988	-0.1774	-0.0983	-0.0771	-0.0491
0.2366	-0.0497	-0.2988	-0.1775	-0.0980	-0.0771	-0.0491
0.0000	0.0000	0.0000	0.0000	0.0000	0.0000	0.0000
0.0000	0.0000	0.0000	0.0000	0.0000	0.0000	0.0000
0.0000	0.0000	0.0000	0.0000	0.0000	0.0000	0.0000
0.0000	0.0000	0.0000	0.0000	0.0000	0.0000	0.0000
0.0000	0.0000	0.0000	0.0000	0.0000	0.0000	0.0000
0.0000	0.0000	0.0000	0.0000	0.0000	0.0000	0.0000
0.0000	0.0000	0.0000	0.0000	0.0000	0.0000	0.0000
0.0000	0.0000	0.0000	0.0000	0.0000	0.0000	0.0000
-0.2366	0.0497	0.2988	0.1775	0.0980	0.0771	0.0491
-0.2366	0.0499	0.2988	0.1774	0.0983	0.0771	0.0491
1.9522	2.6178	1.8554	1.1922	3.0768	0.5434	2.2192
1.9522	2.6178	1.8553	1.1922	3.0769	0.5435	2.2192

Ring 8	Ring 9	Ring 10	Ring 11	Ring 12	Ring 13	Ring 14	Ring 15
-1.9079	-0.2860	-0.5705	-0.9579	-1.3944	-0.9734	-0.4280	-0.4664
-1.9079	-0.2859	-0.5705	-0.9578	-1.3945	-0.9734	-0.4279	-0.4665
-0.1286	-0.0146	-0.0367	-0.0286	-0.0644	-0.2555	-0.1211	-0.0581
-0.1287	-0.0147	-0.0366	-0.0286	-0.0644	-0.2556	-0.1210	-0.0581
0.0000	0.0000	0.0000	0.0000	0.0000	0.0000	0.0000	0.0000
0.0000	0.0000	0.0000	0.0000	0.0000	0.0000	0.0000	0.0000
0.0000	0.0000	0.0000	0.0000	0.0000	0.0000	0.0000	0.0000
0.0000	0.0000	0.0000	0.0000	0.0000	0.0000	0.0000	0.0000
0.0000	0.0000	0.0000	0.0000	0.0000	0.0000	0.0000	0.0000
0.0000	0.0000	0.0000	0.0000	0.0000	0.0000	0.0000	0.0000
0.0000	0.0000	0.0000	0.0000	0.0000	0.0000	0.0000	0.0000
0.0000	0.0000	0.0000	0.0000	0.0000	0.0000	0.0000	0.0000
0.1287	0.0147	0.0366	0.0286	0.0644	0.2556	0.1210	0.0581
0.1286	0.0146	0.0367	0.0286	0.0644	0.2555	0.1211	0.0581
1.9079	0.2859	0.5705	0.9578	1.3945	0.9734	0.4279	0.4665
1.9079	0.2860	0.5705	0.9579	1.3944	0.9734	0.4280	0.4664

The diagrams below show SYSMO[19] *ipso*-centric pseudo-π[20] calculations performed on the tetracation and tetraanion of **5-3-2CG**.

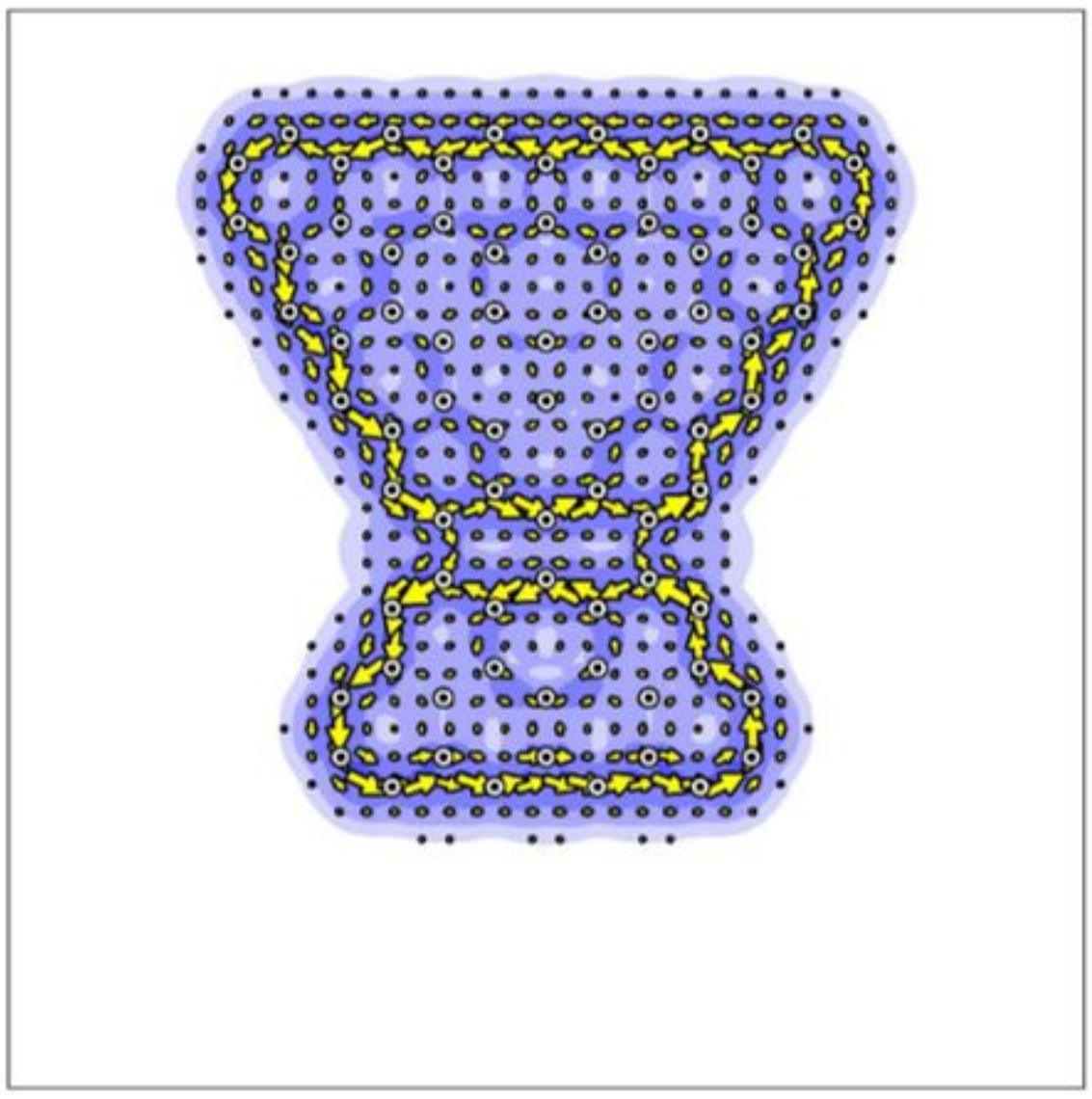

Tetracation of 5-3-2CG

Figure S20.

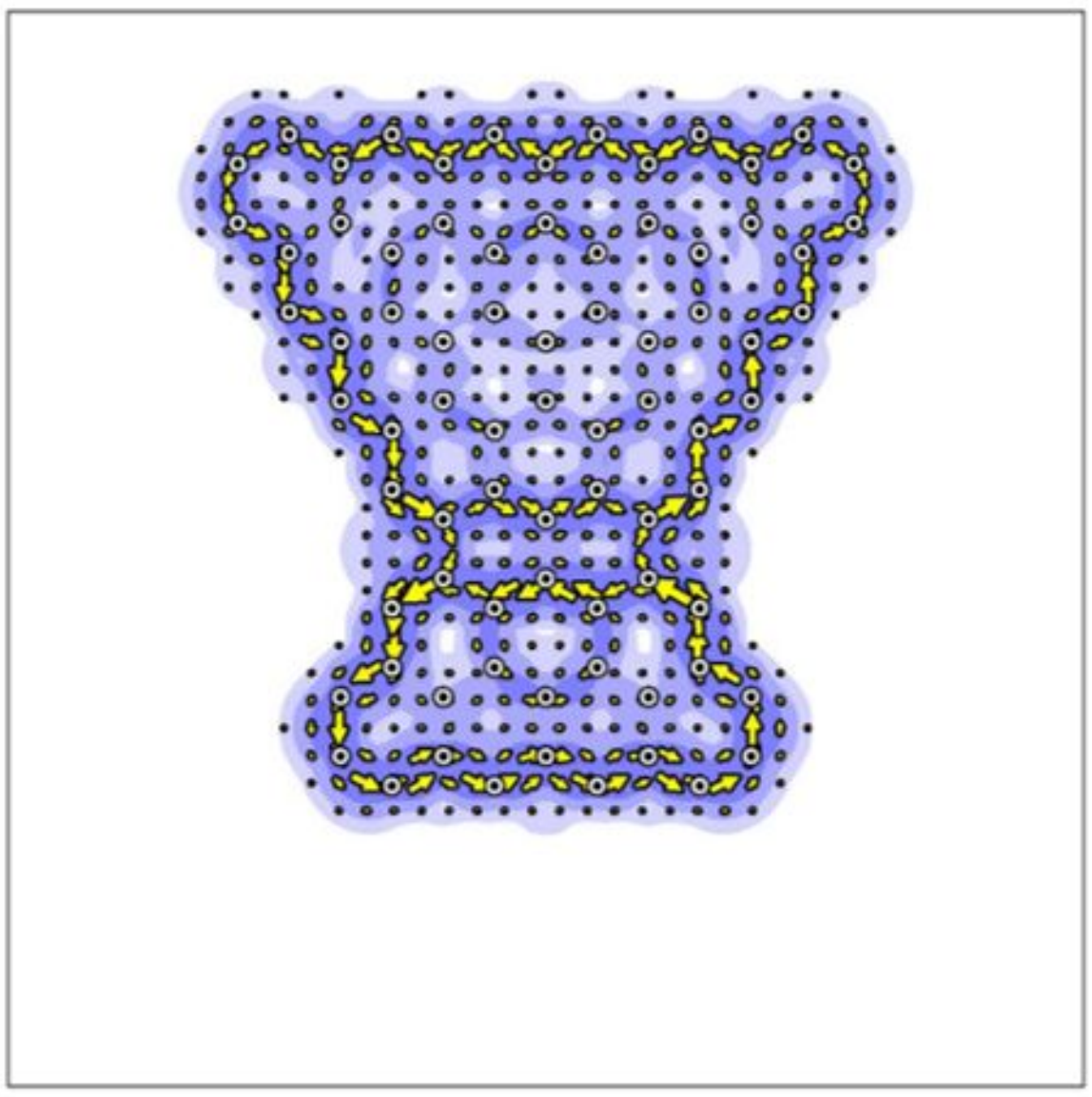

Tetraanion of 5-3-2CG

Figure S21.

Kekulé structures

It is possible to construct Kekulé structures with the requested number of unpaired electrons and with no double bonds vertically located across the central ring motif, as identified in Table 6 of the main text, as shown below in SCHEME S1:

SCHEME S1.

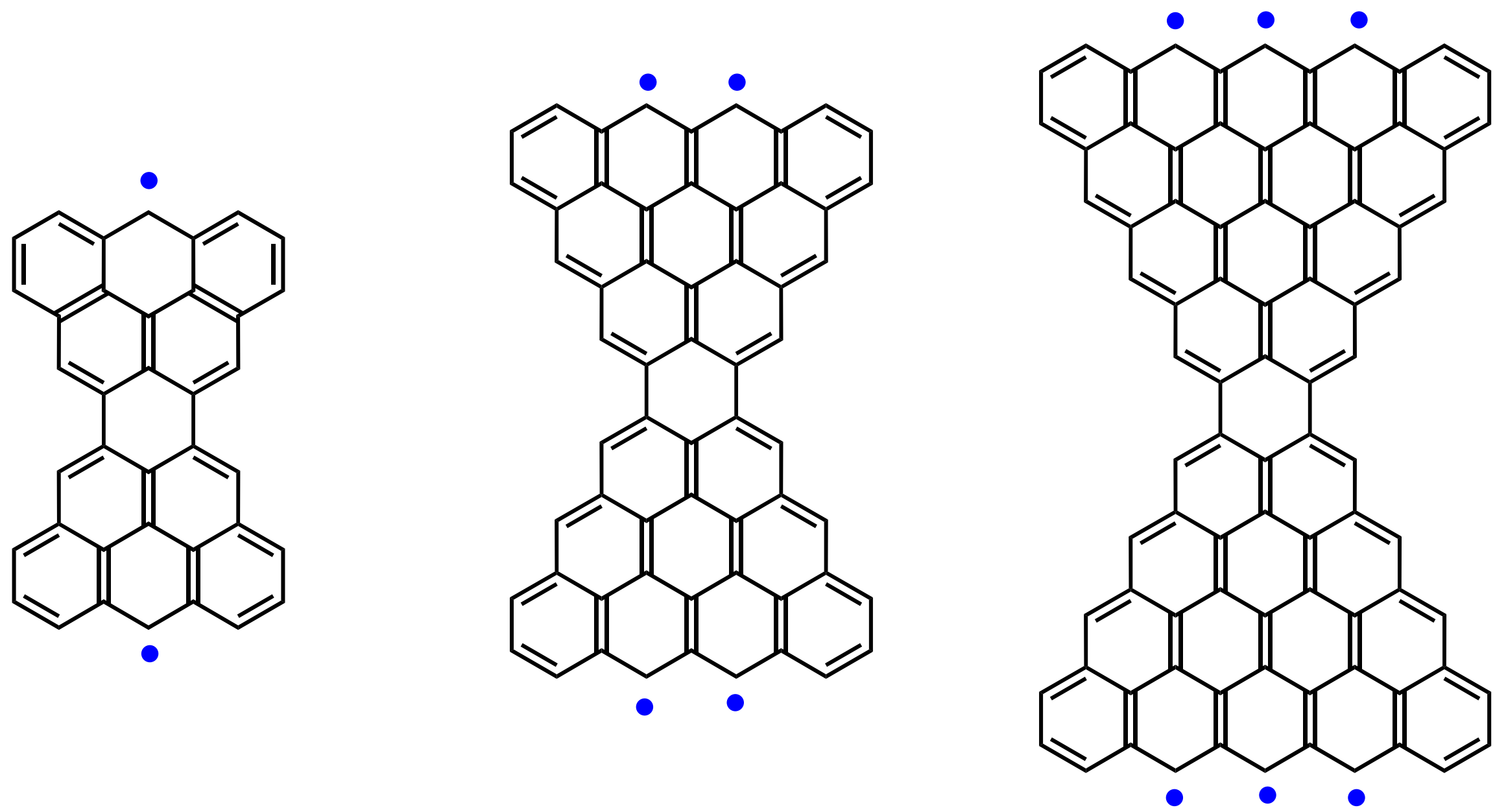

3-3-1CG 4-4-1CG 5-5-1CG

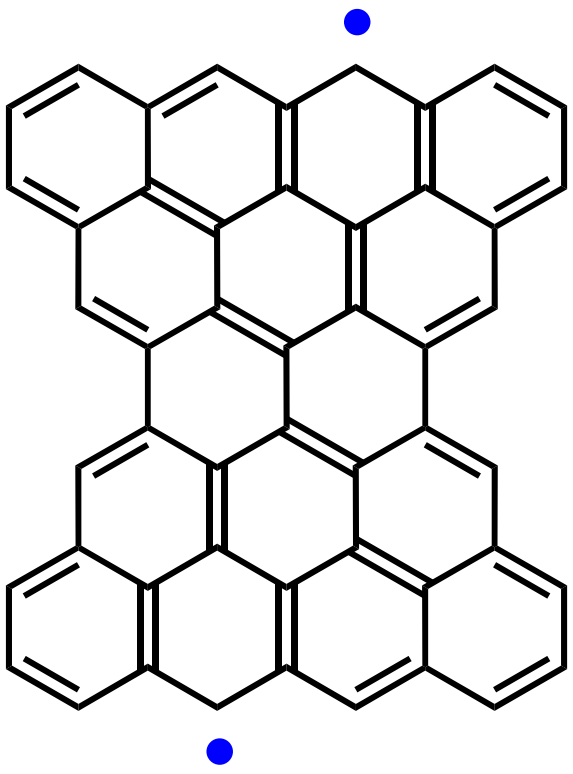

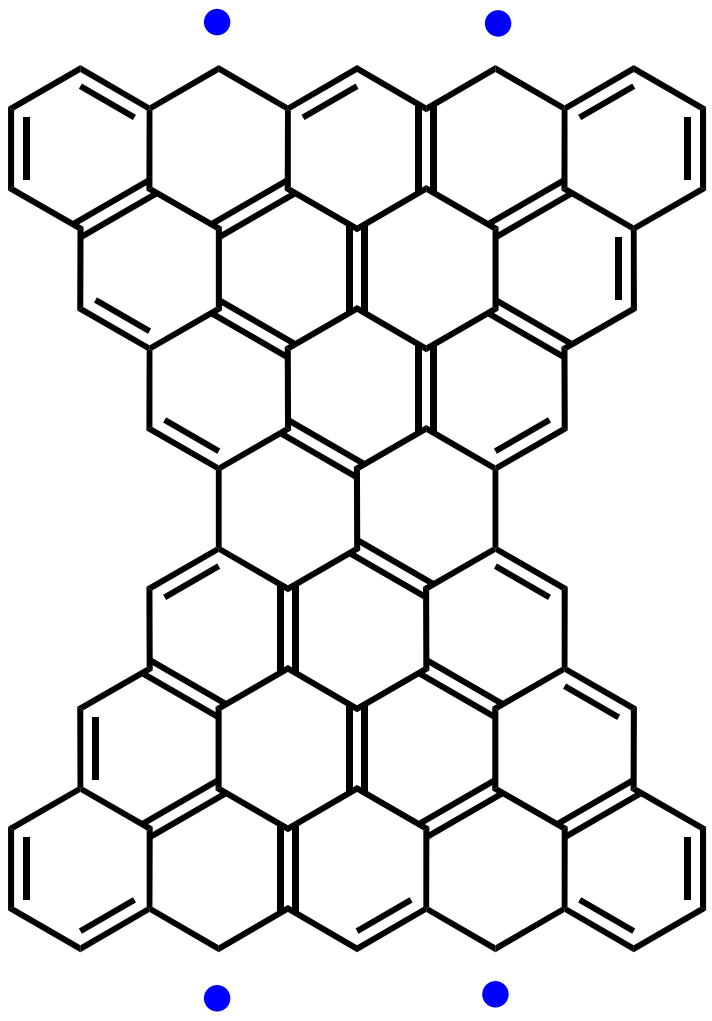

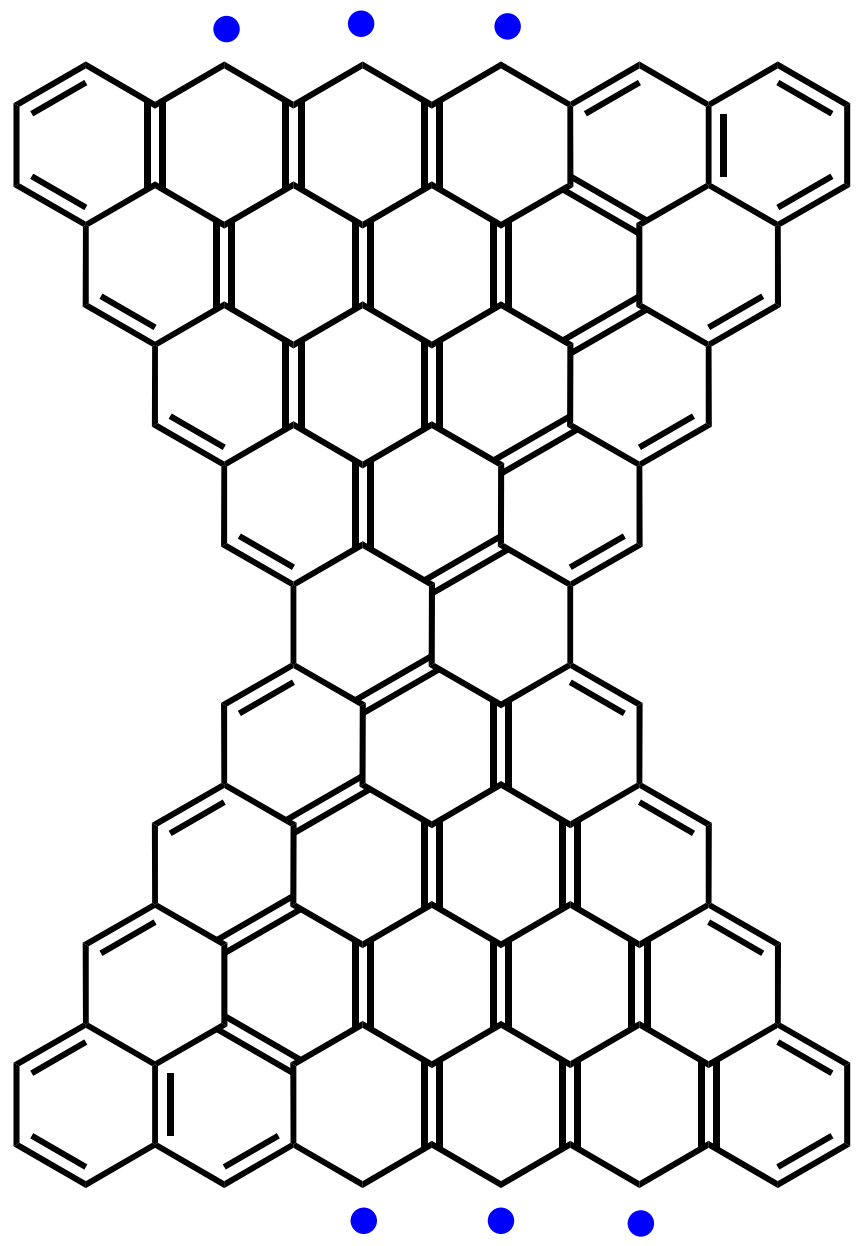

3-3-2CG **4-4-2CG** **5-5-2CG**

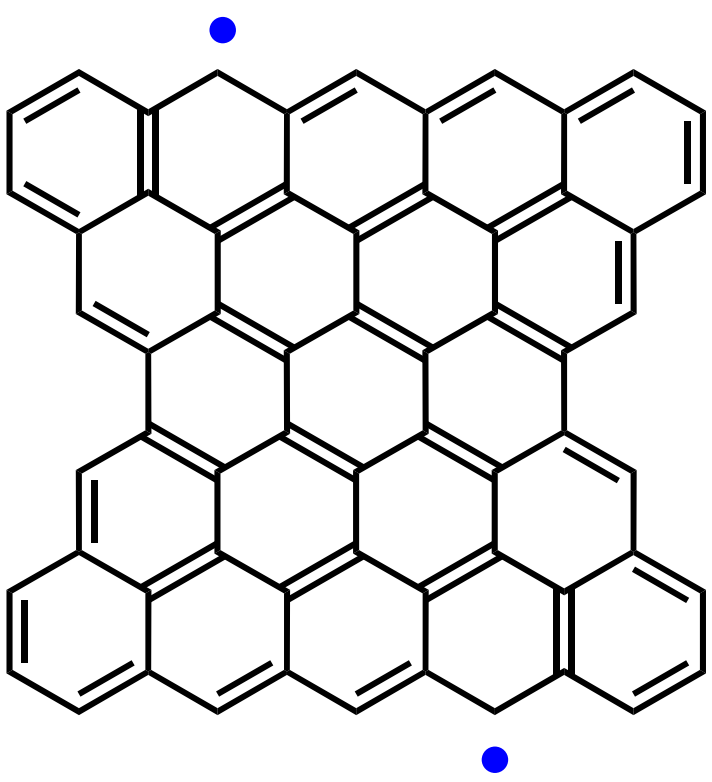

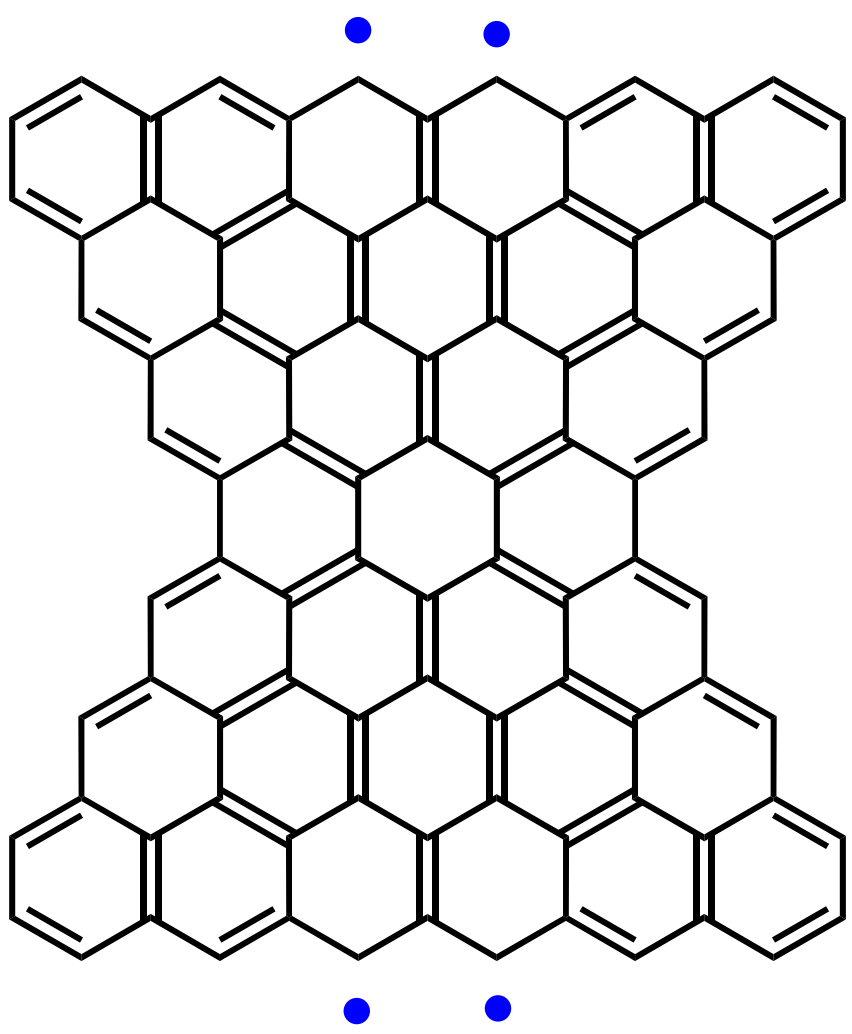

3-3-3CG

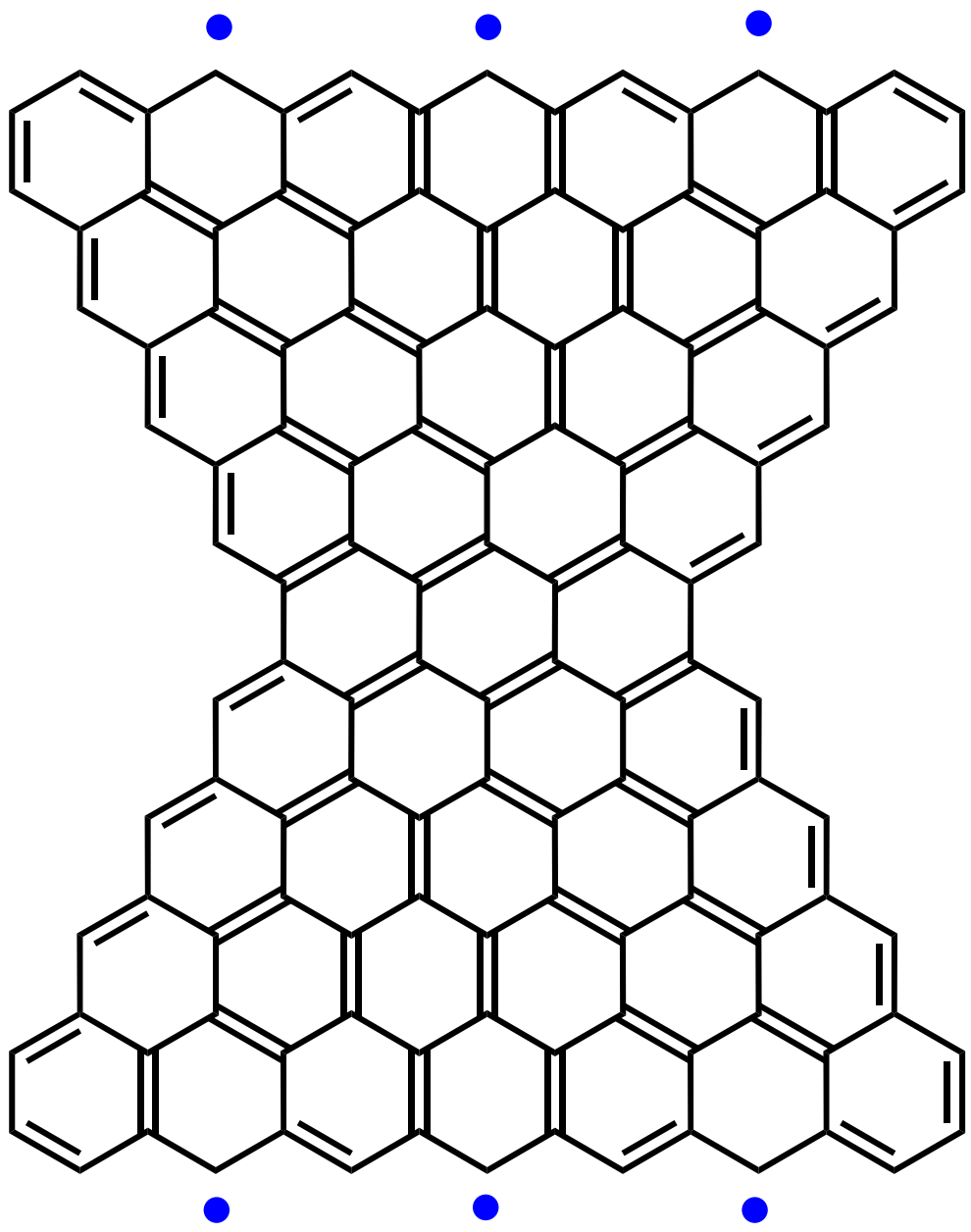

4-4-3CG

5-5-3CG

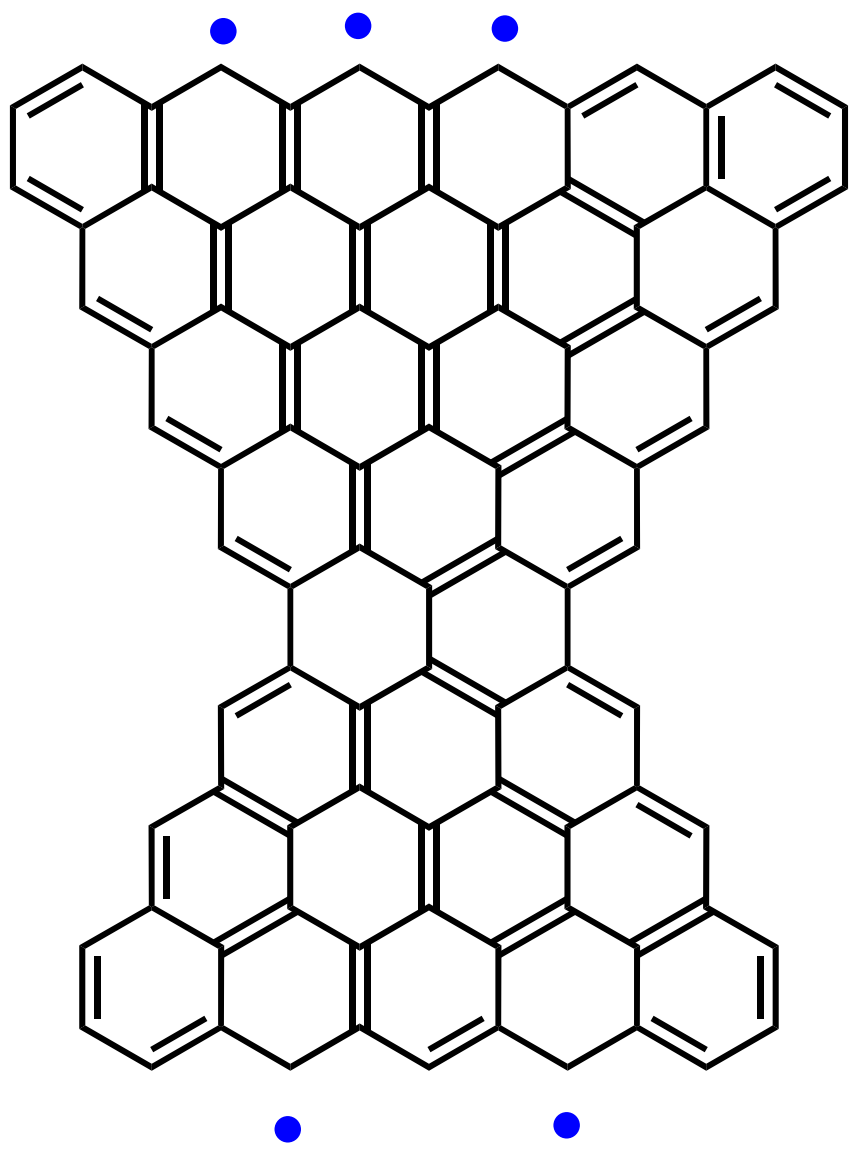

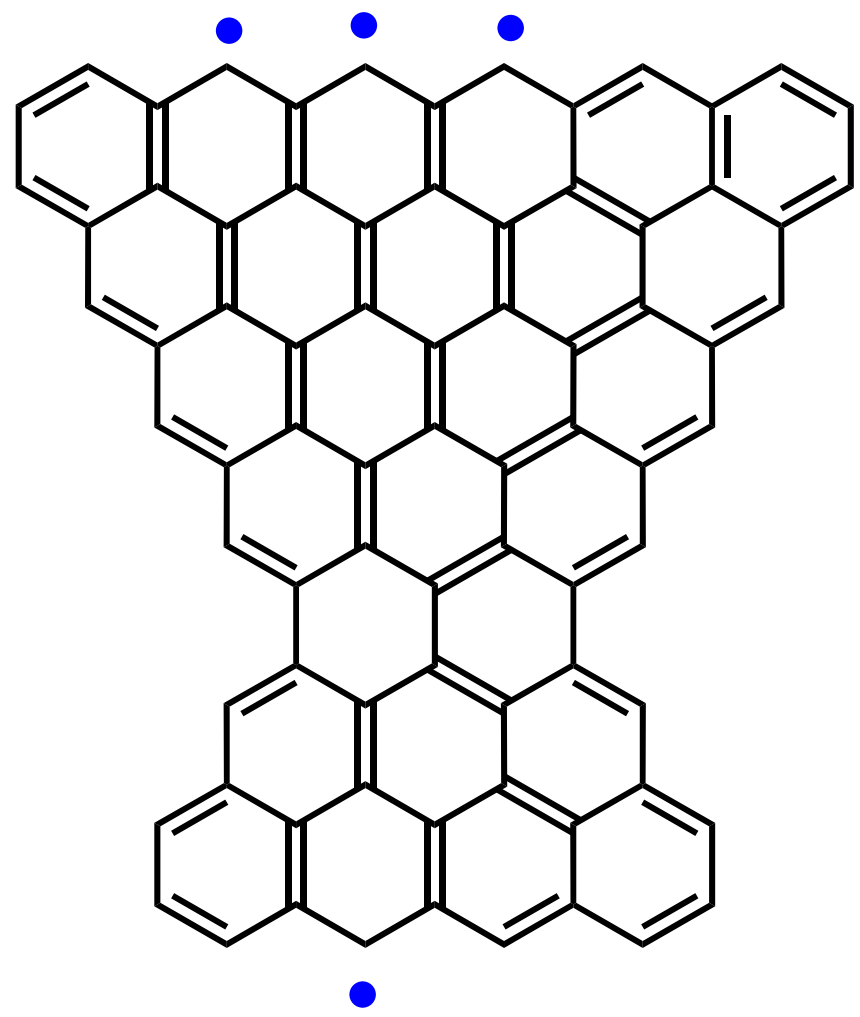

5-4-2CG

5-3-2CG

Frontier Orbitals

This section contains diagrams of the frontier molecular orbitals displayed from their atomic orbital components. The frontier orbitals of the neutral species are displayed for the following oxidation states:

- LUMO +1 – Orange
- LUMO(S) – Yellow
- Shared HOMO/LUMO – Green (5-4-2CG only)
- HOMO(S) Blue
- HOMO -1 - Purple

In all cases, the HOMO and LUMO orbitals are degenerate with an associated eigenvalue of zero, thus forming non-bonding orbitals. As the HOMO and LUMO orbitals are degenerate there are in fact an infinite number of possible solutions. However the eigenvectors associated with each carbon atom are either of opposite sign to their neighbor, or the neighboring atom makes zero contribution to the molecular orbital in question. In all the diagrams below, the size of the orbital contribution is proportional to the size of the spheres displayed. Orbital contributions of opposite sign are displayed in white.

SCHEME S2

3-3-1CG

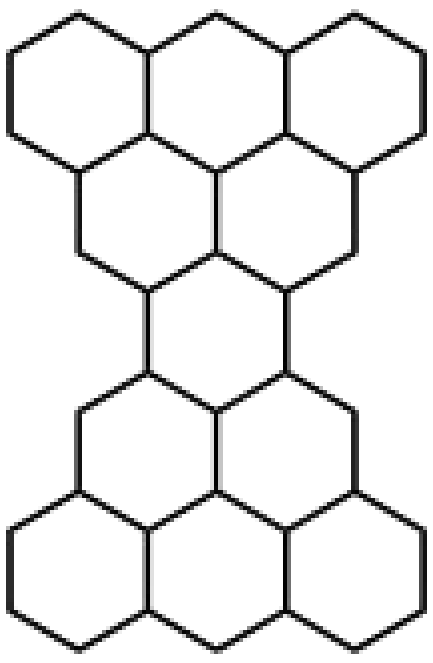

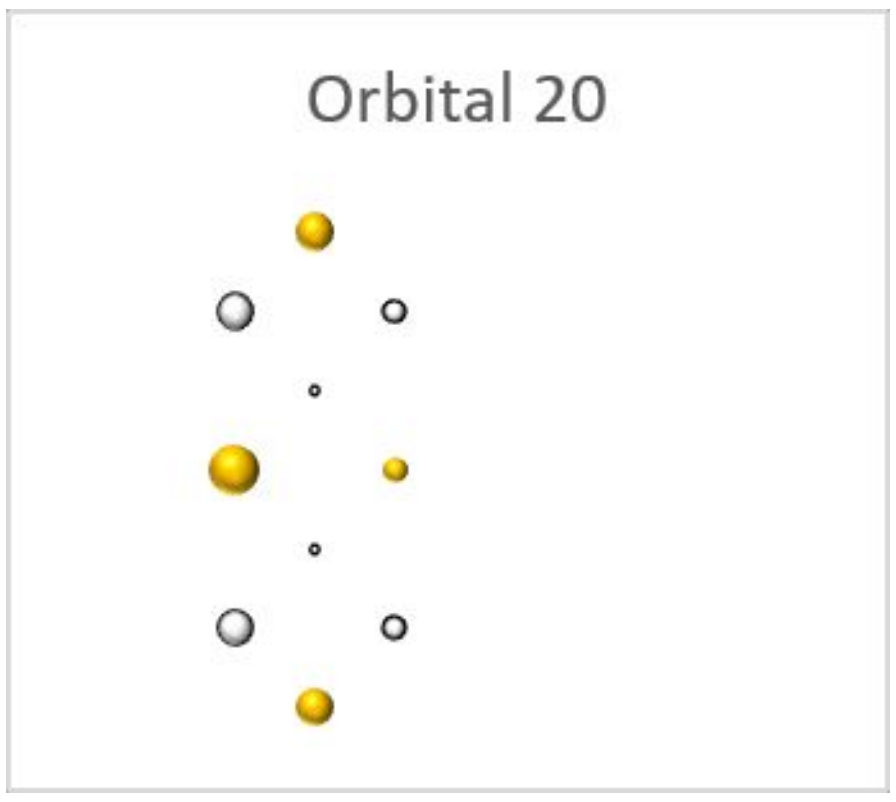
Orbital 20

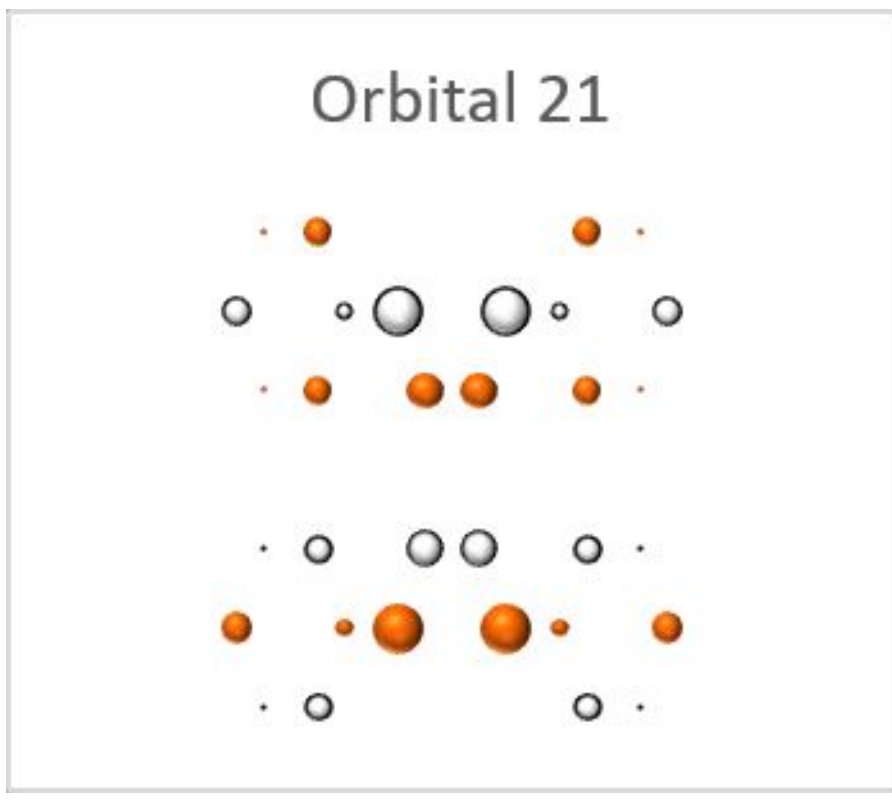
Orbital 21

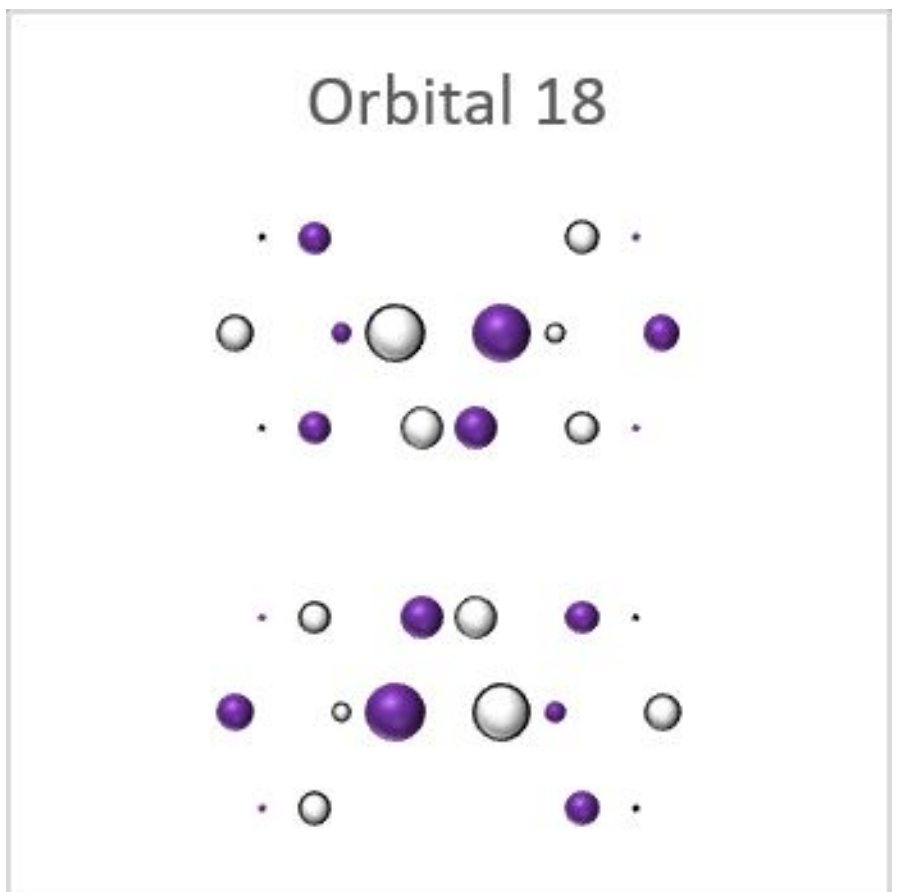
Orbital 18

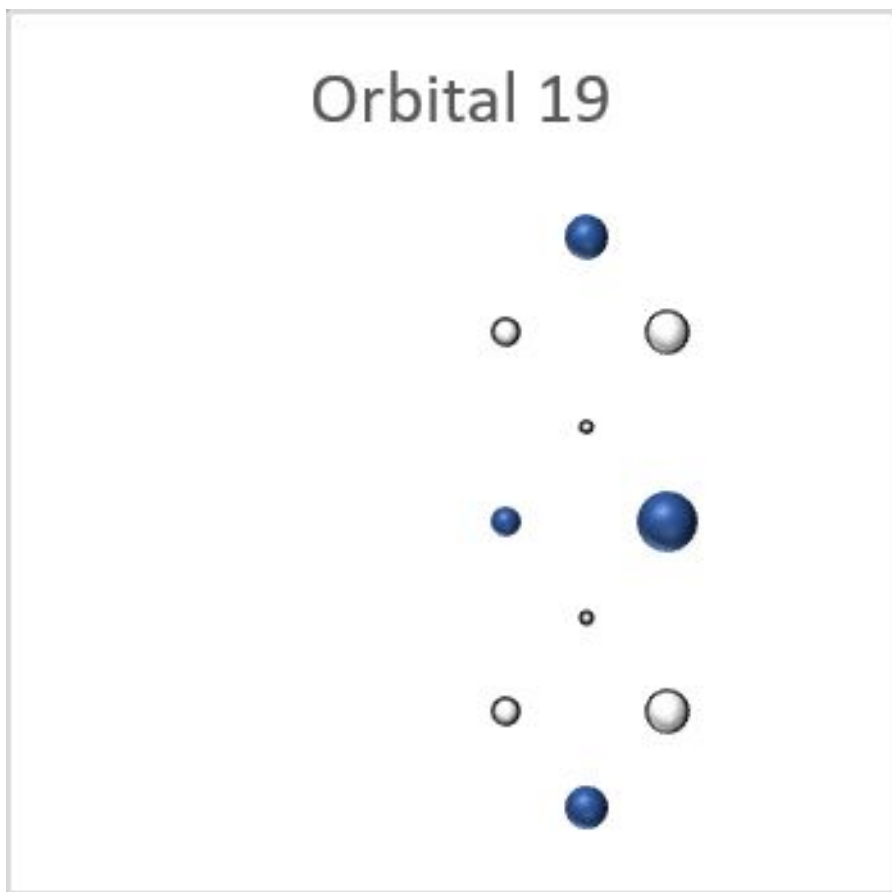
Orbital 19

4-4-1CG

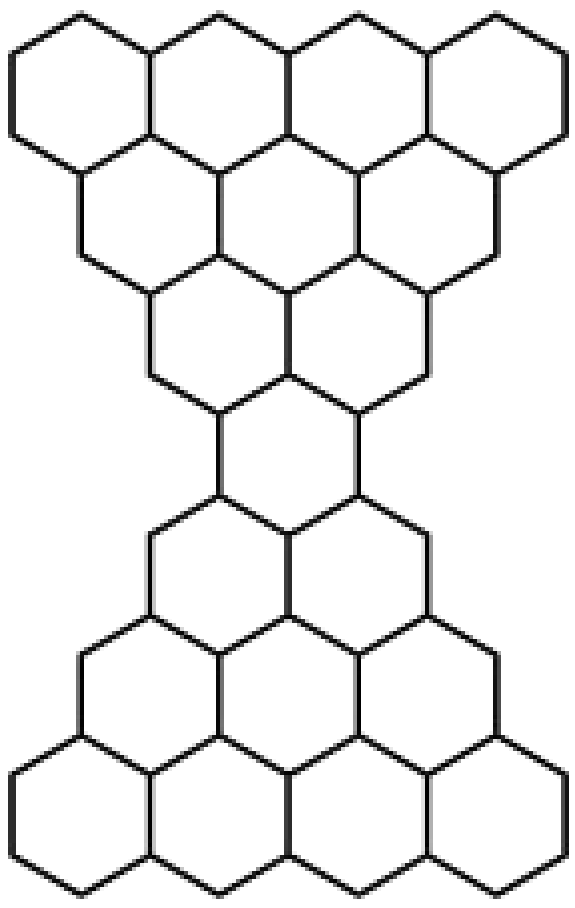

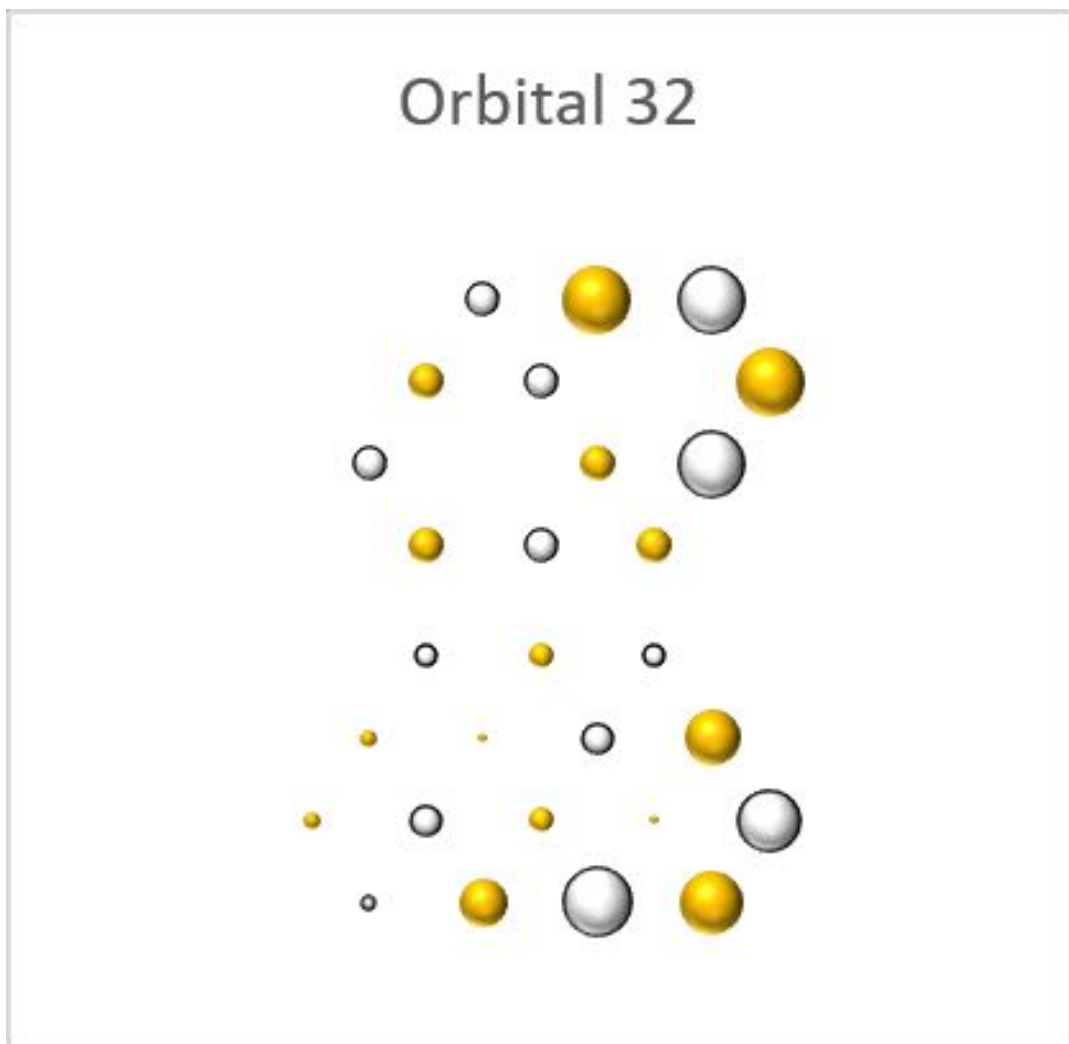
Orbital 32

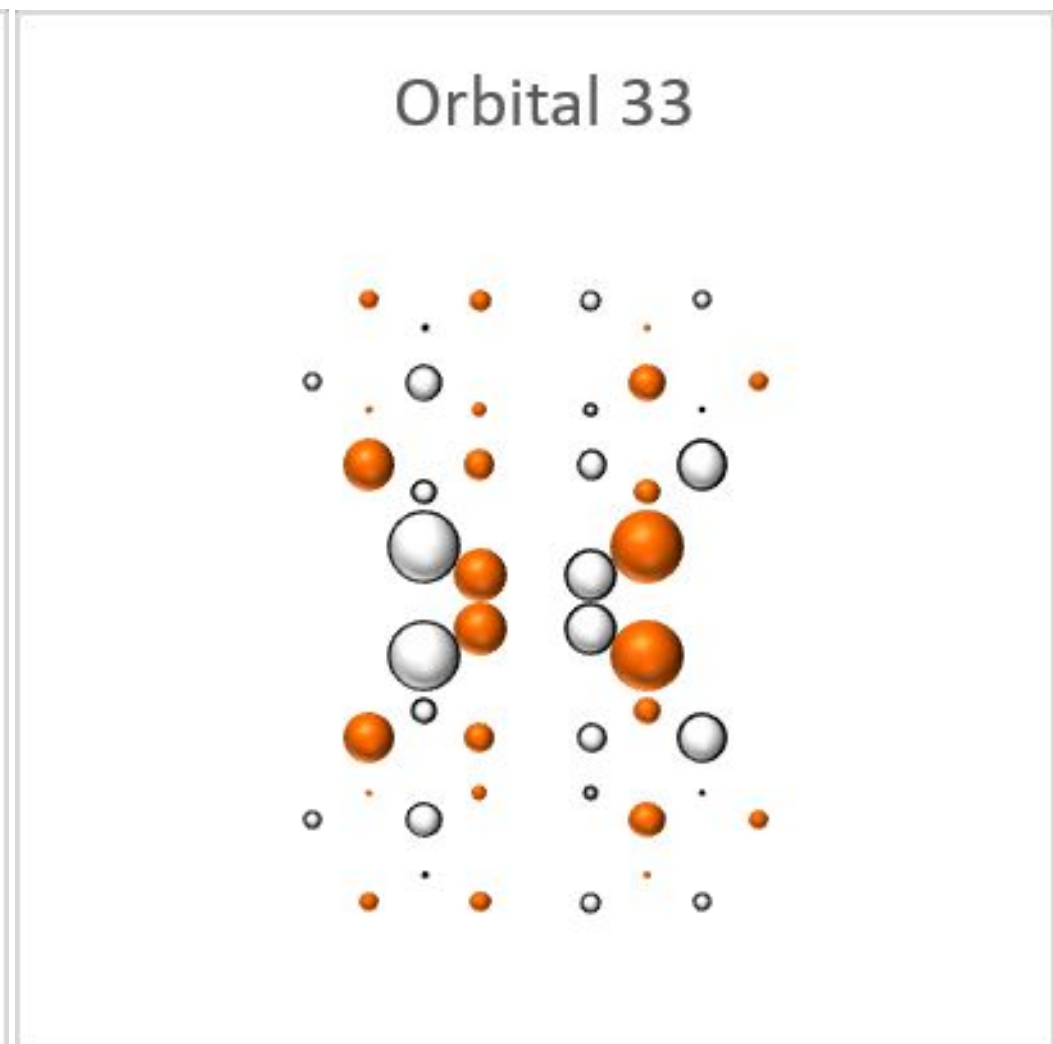
Orbital 33

5-5-1CG

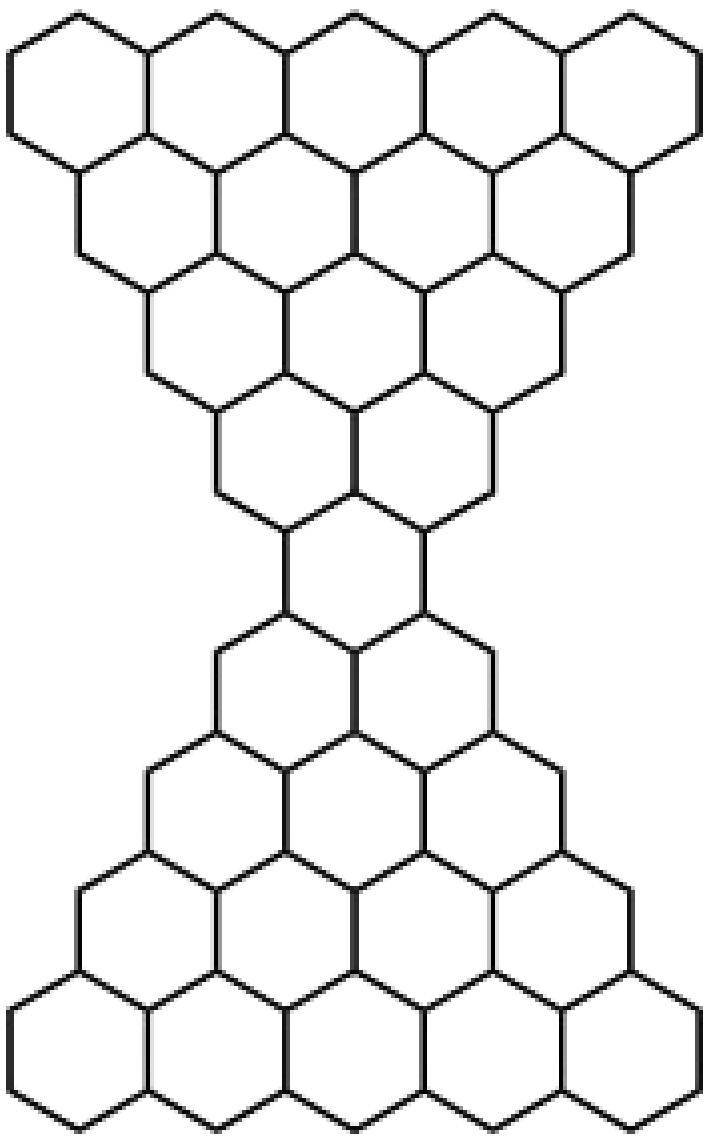

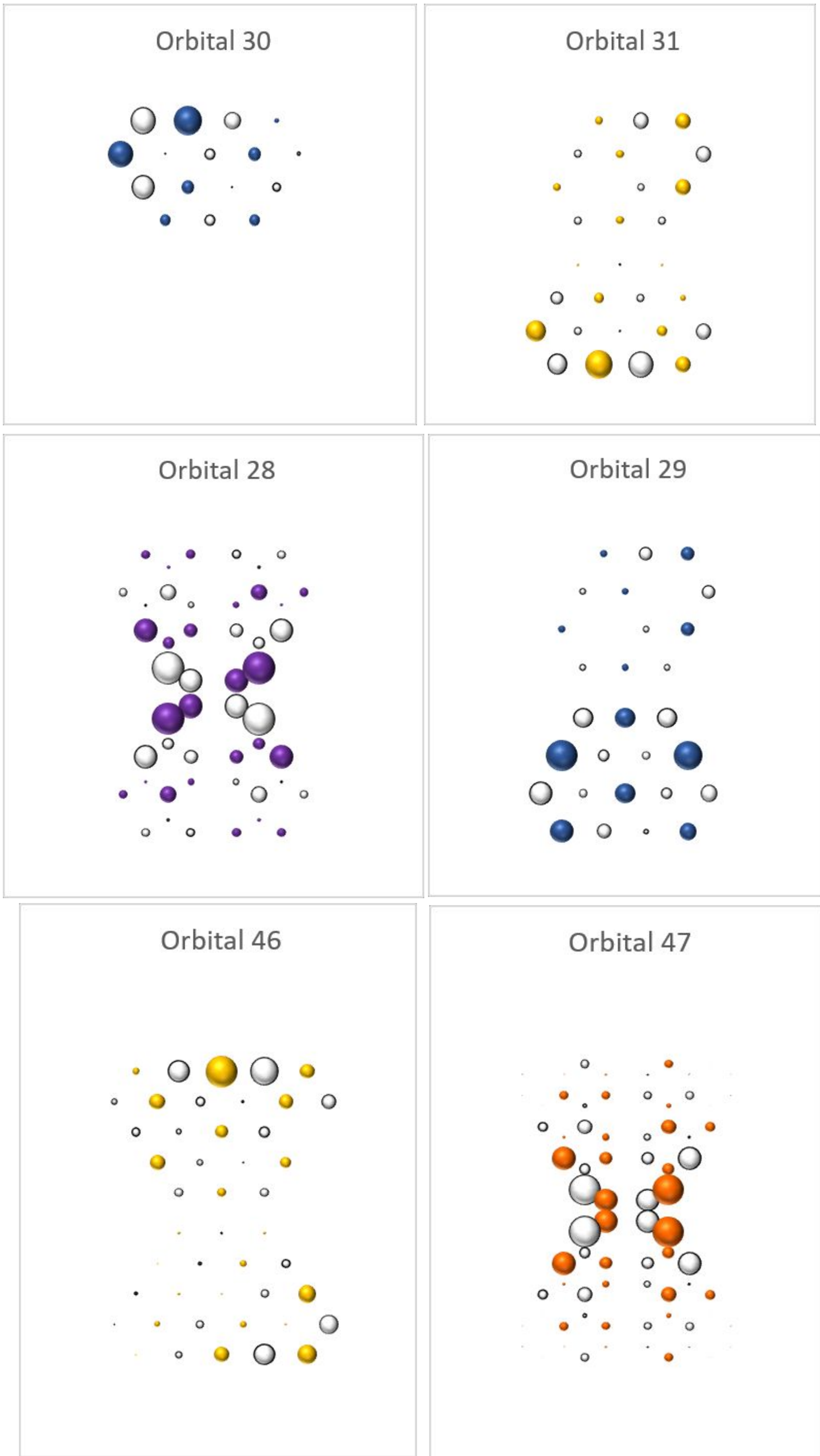
Orbital 30
Orbital 31
Orbital 28
Orbital 29
Orbital 46
Orbital 47

Orbital 43

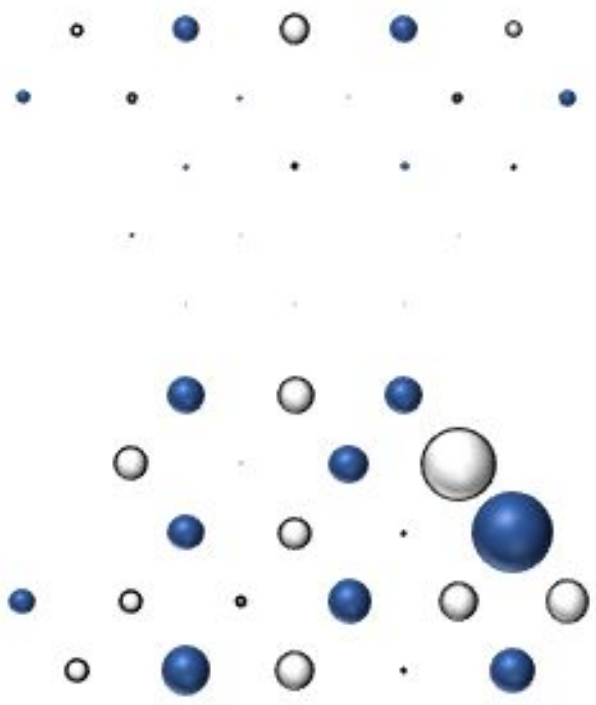

Orbital 44

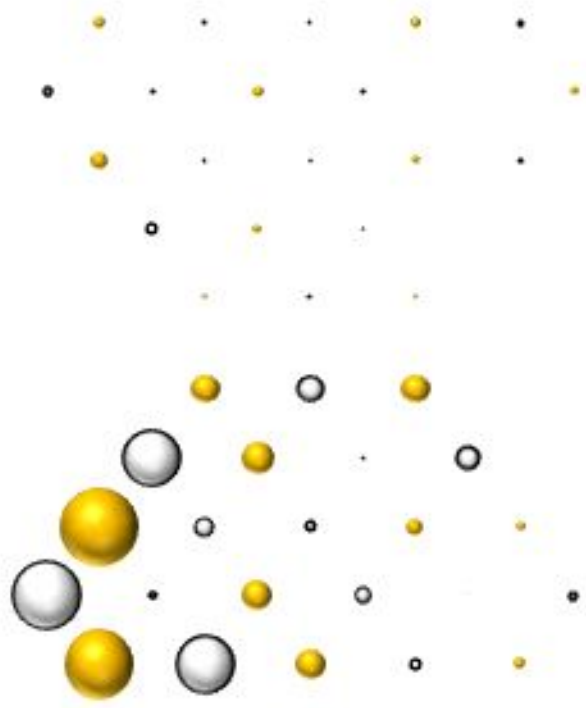

Orbital 45

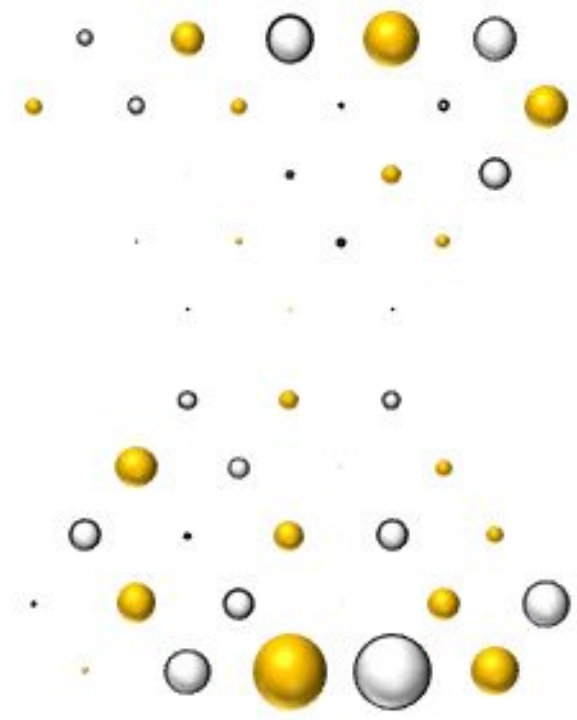

Orbital 40

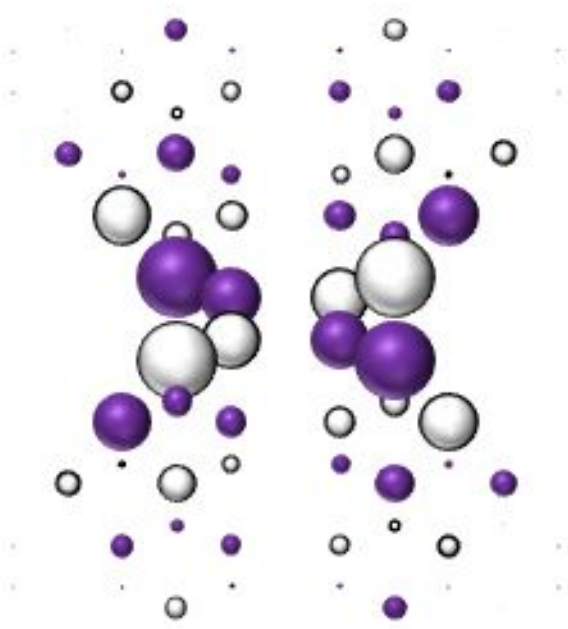

Orbital 41

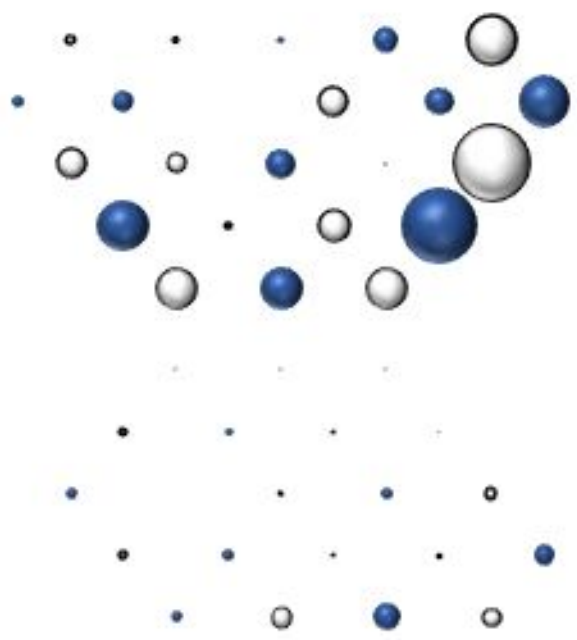

Orbital 42

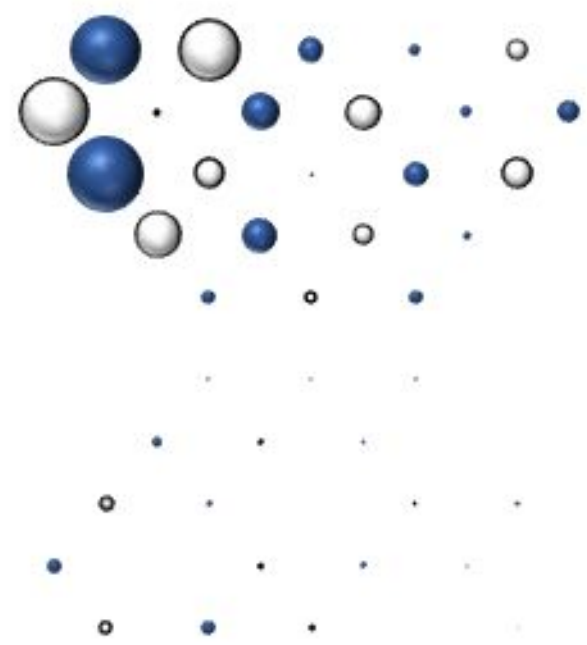

3-3-2CG

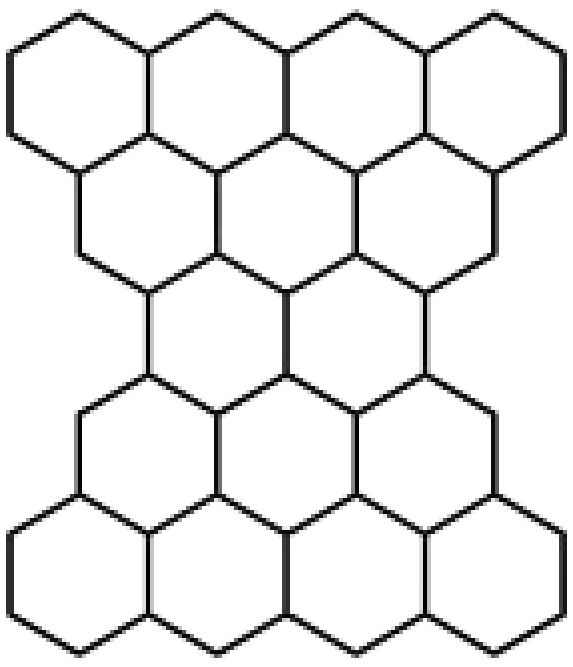

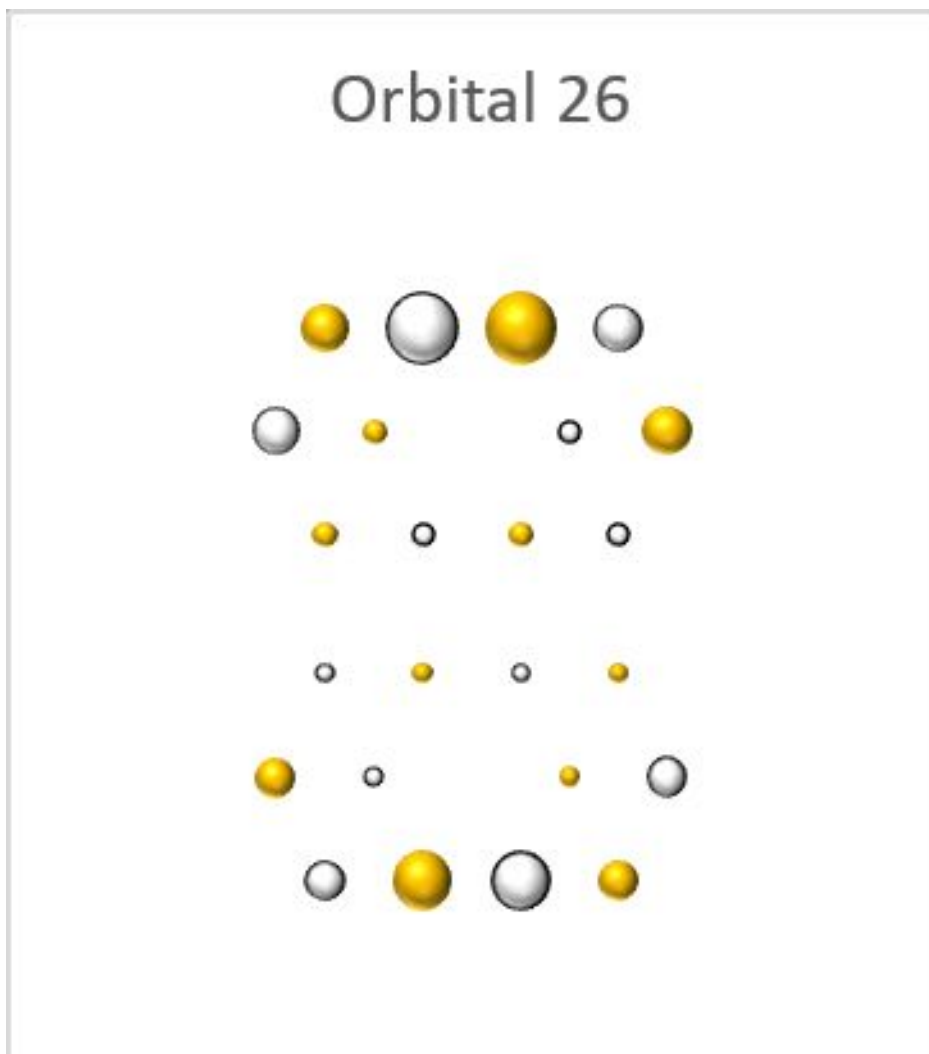
Orbital 26

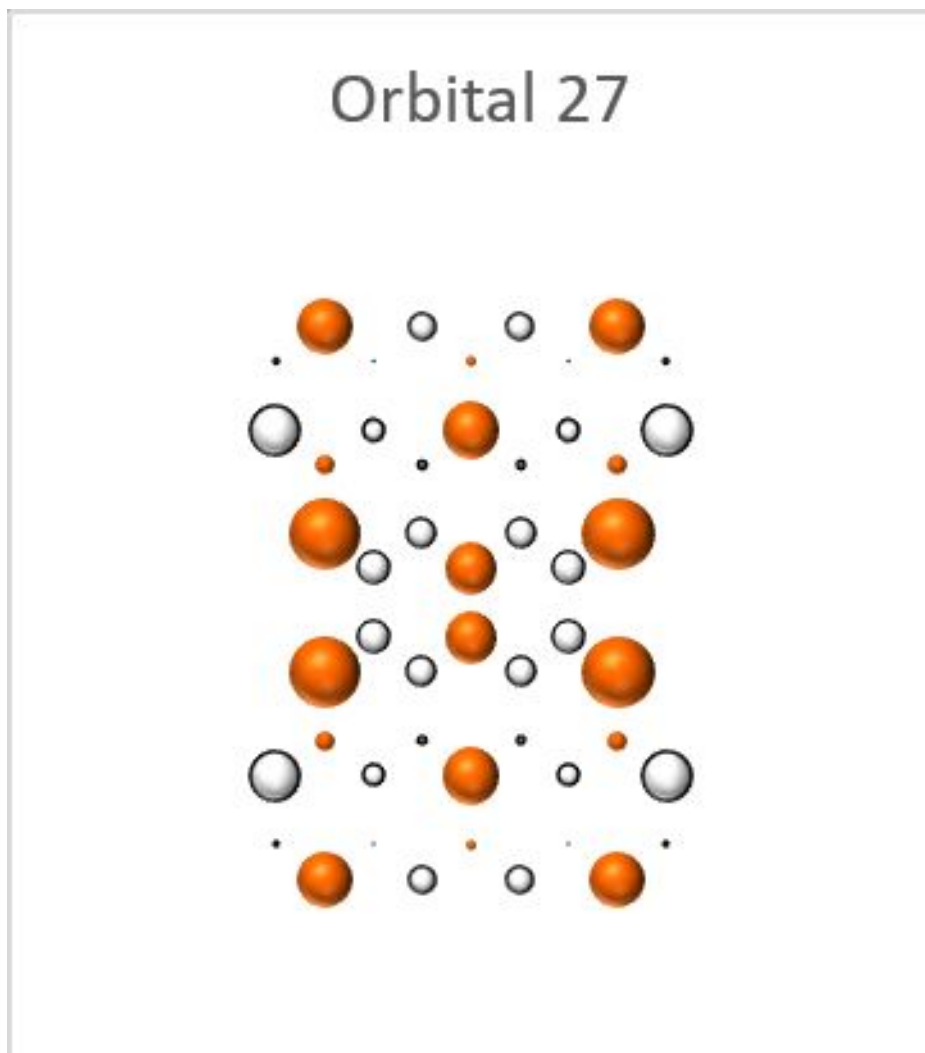
Orbital 27

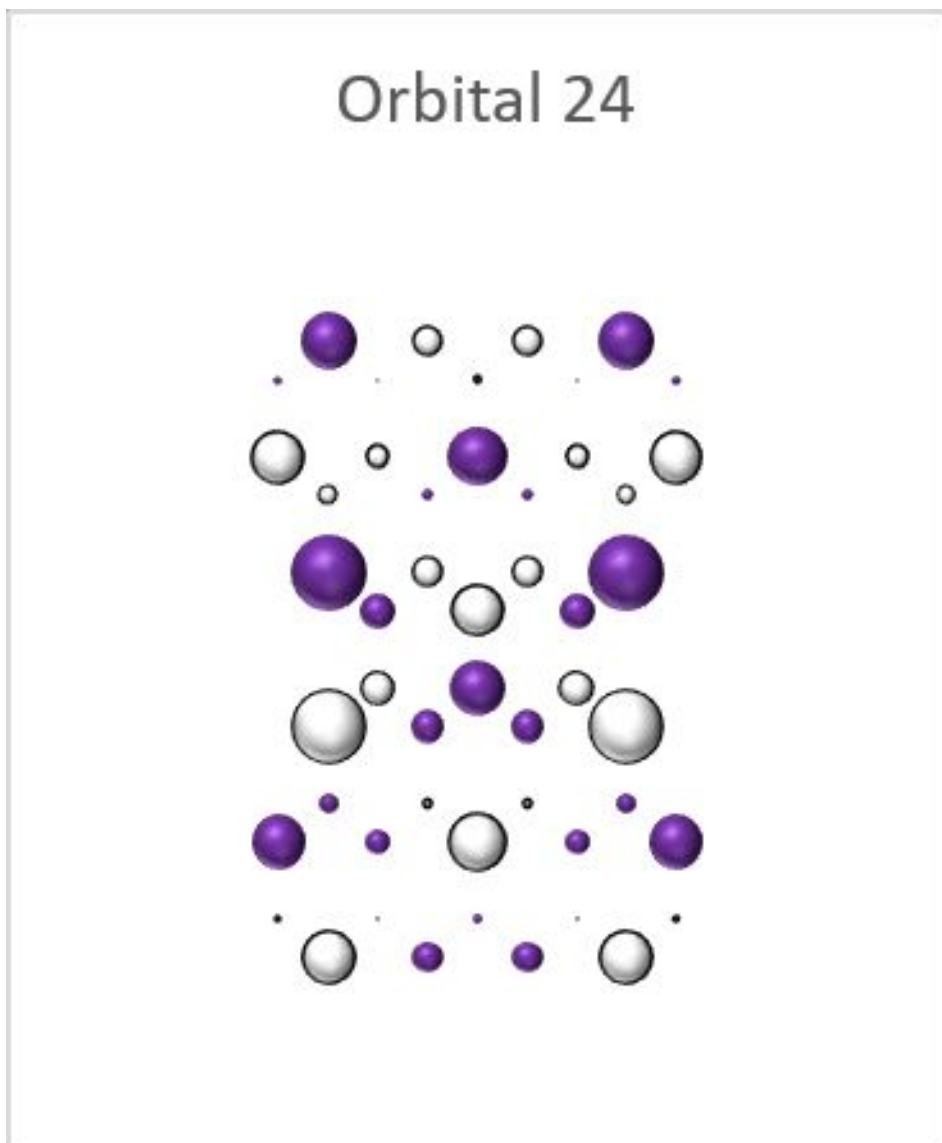
Orbital 24

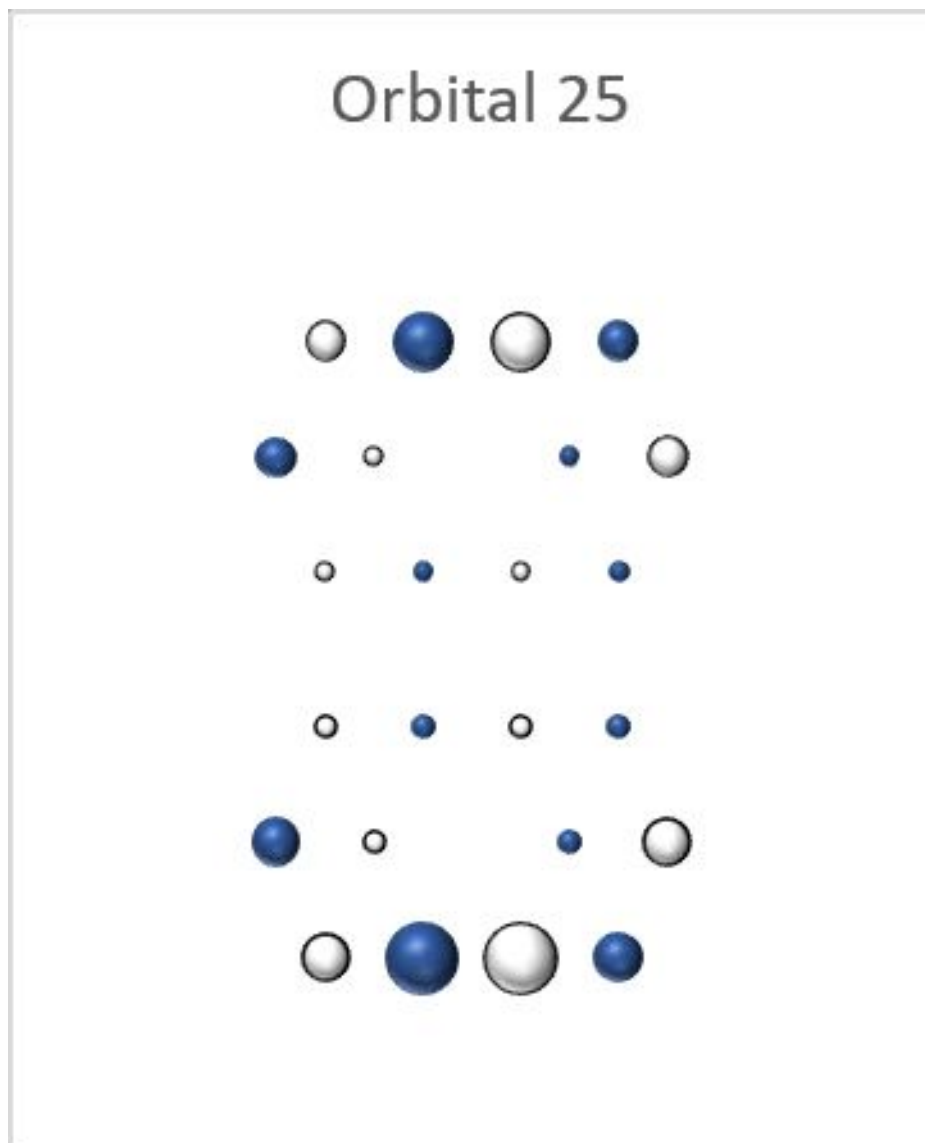
Orbital 25

4-4-2CG

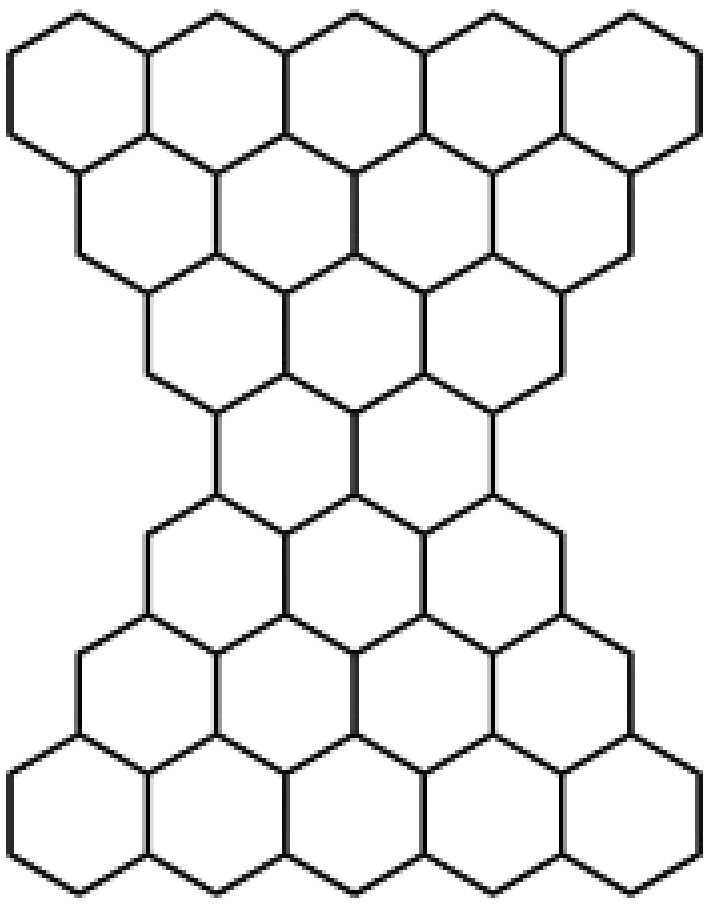

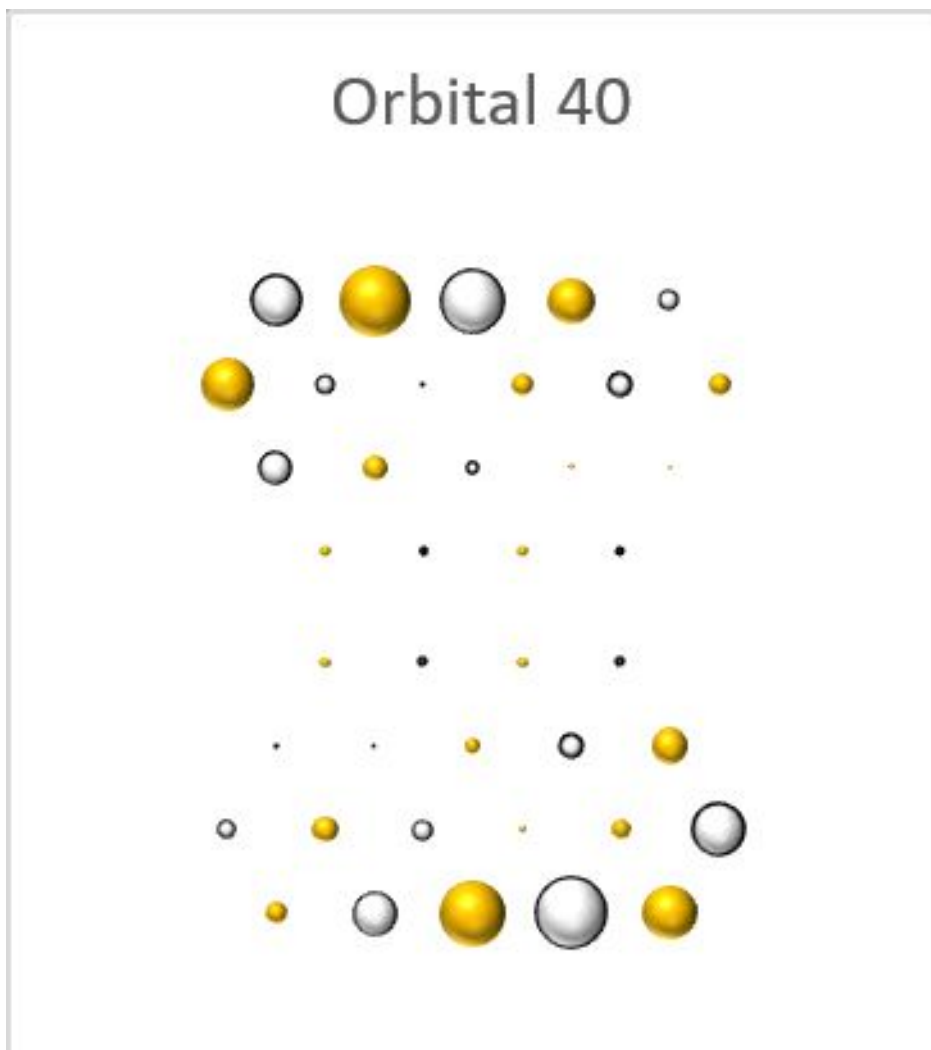
Orbital 40

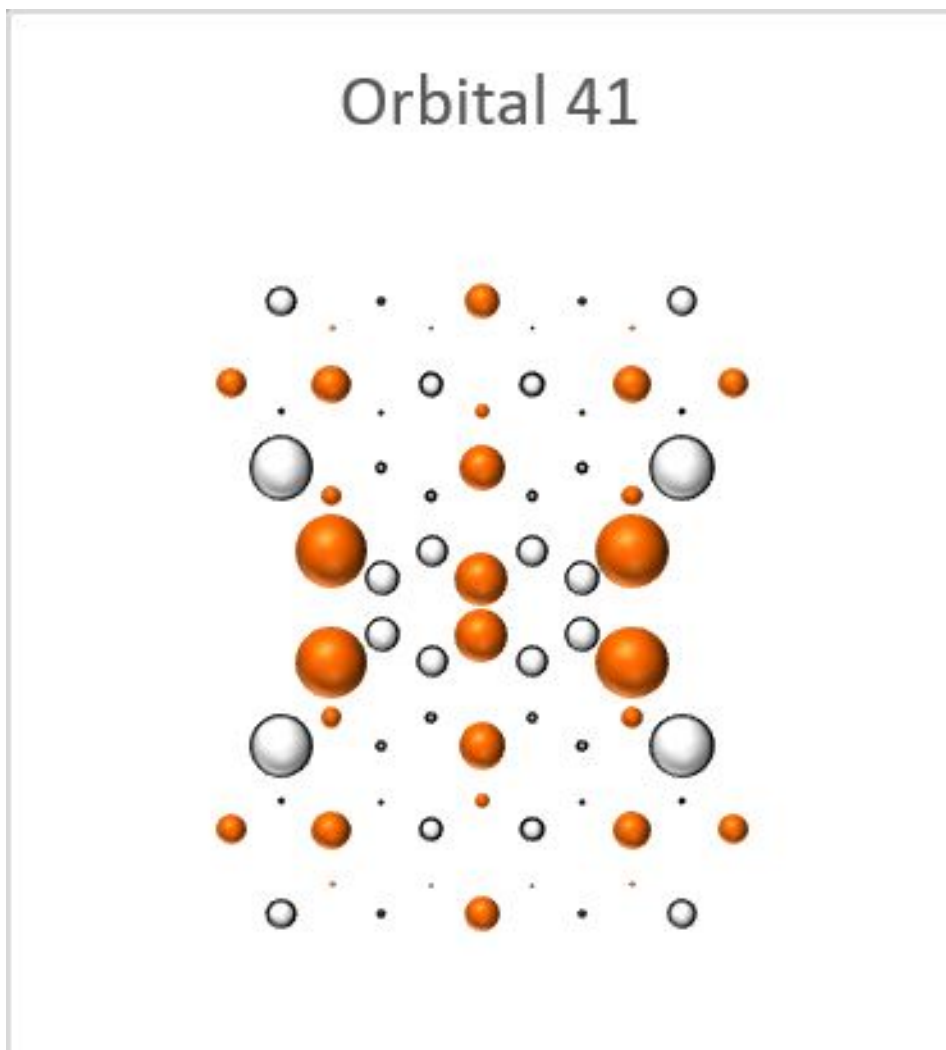
Orbital 41

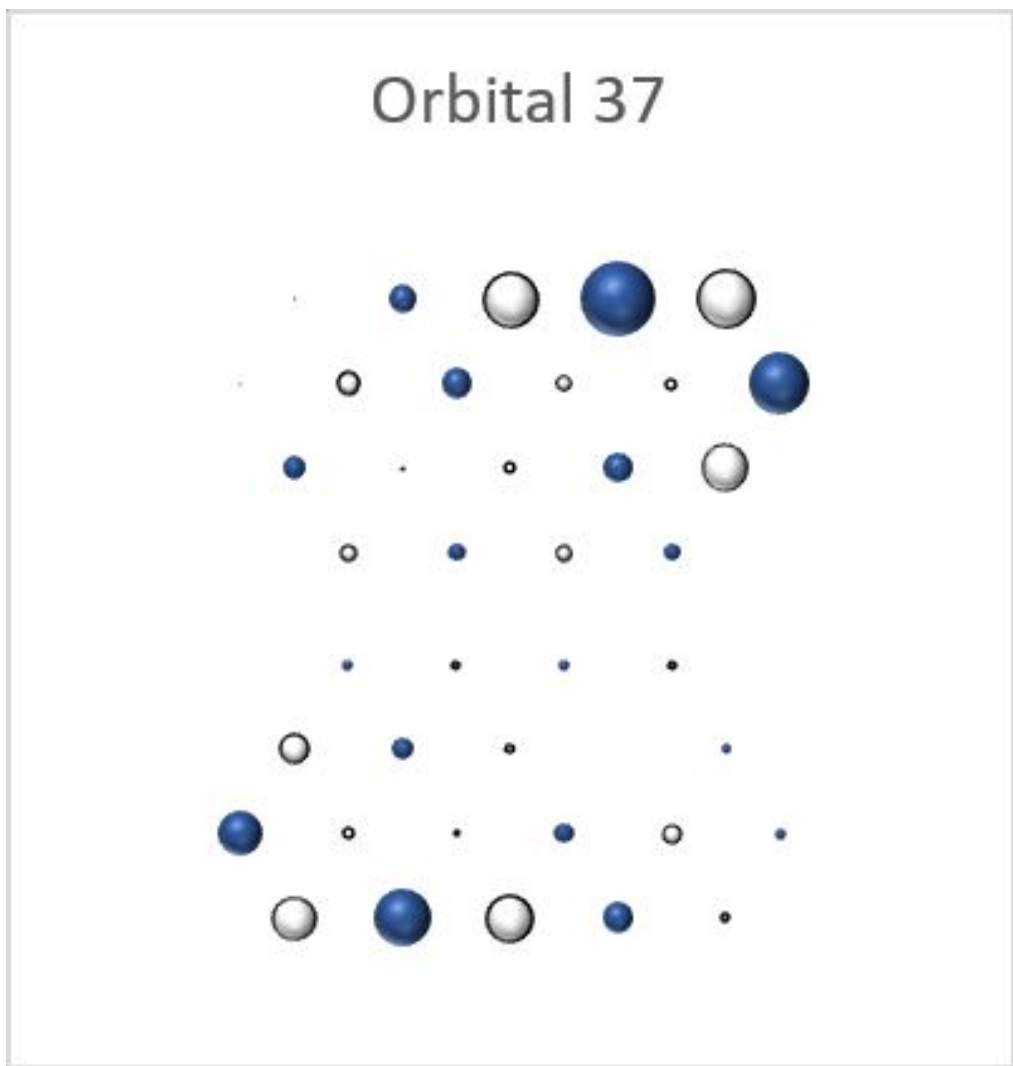
Orbital 37

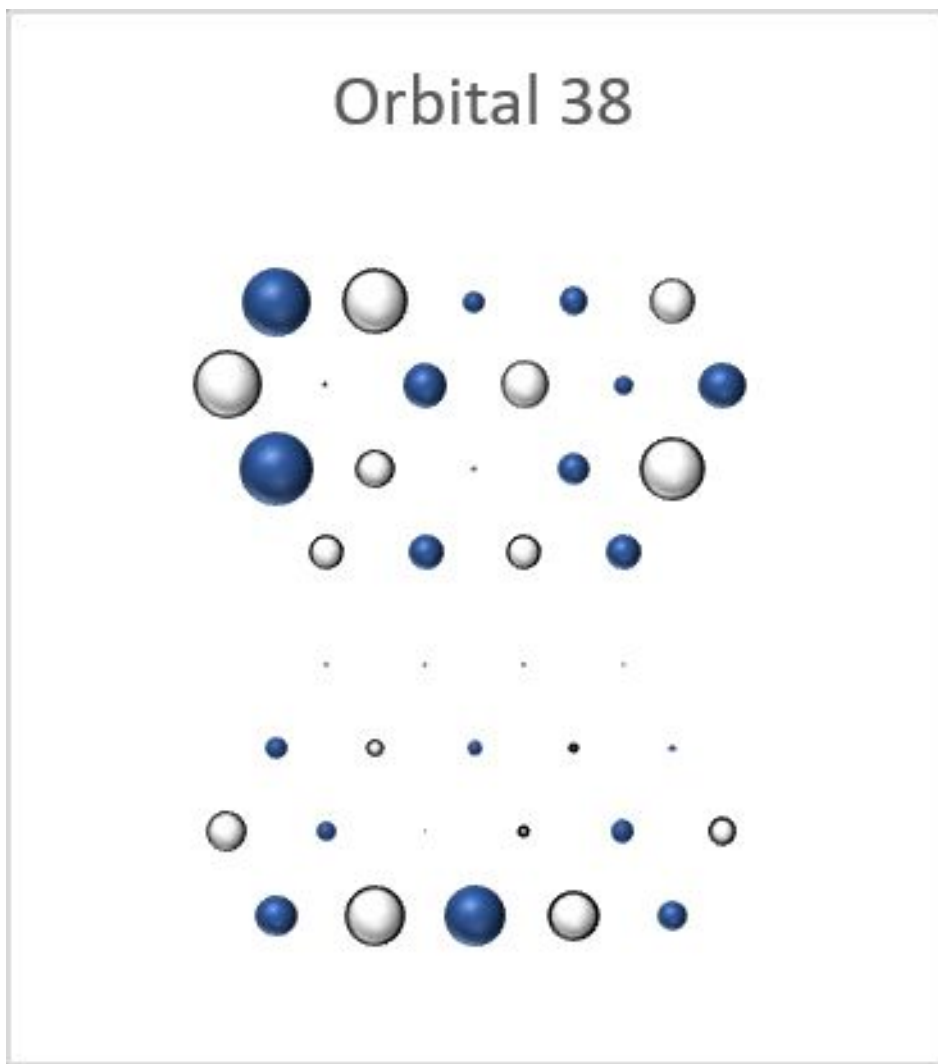
Orbital 38

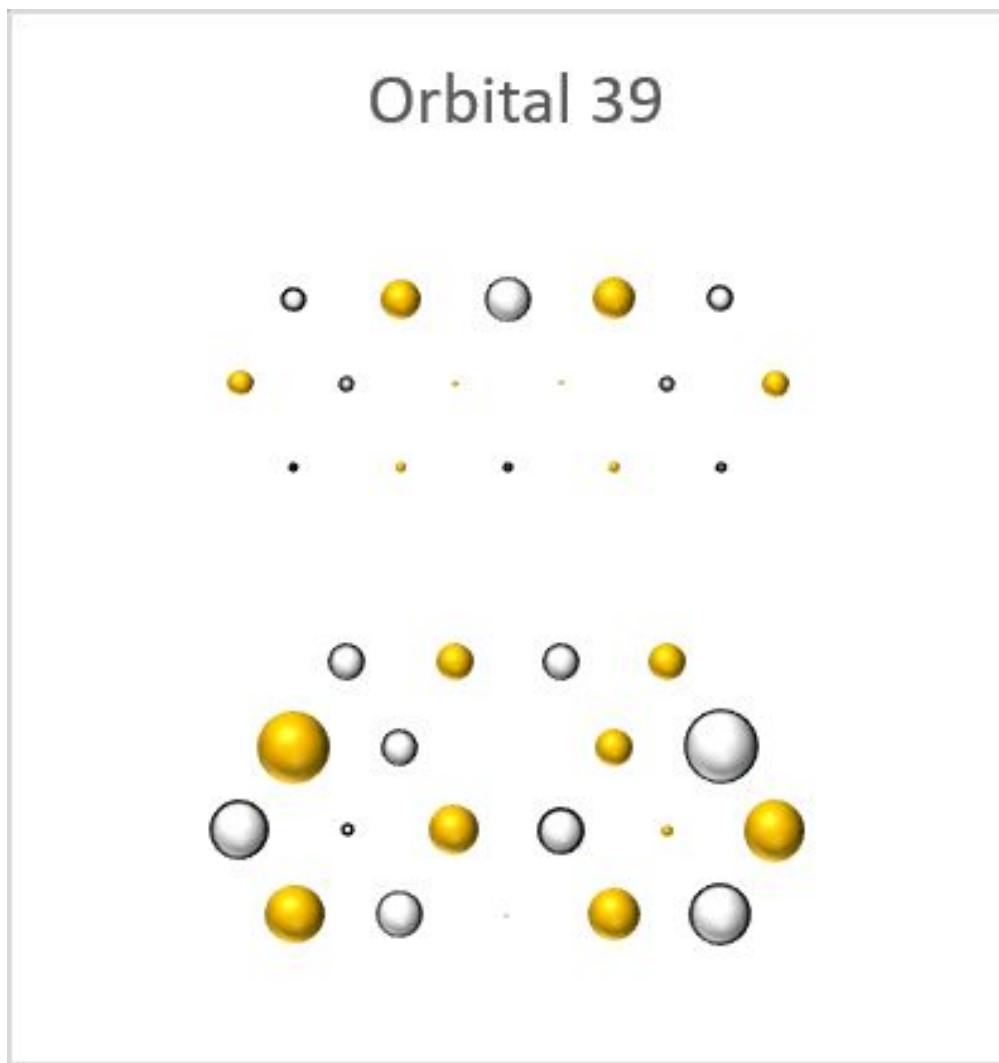
Orbital 39

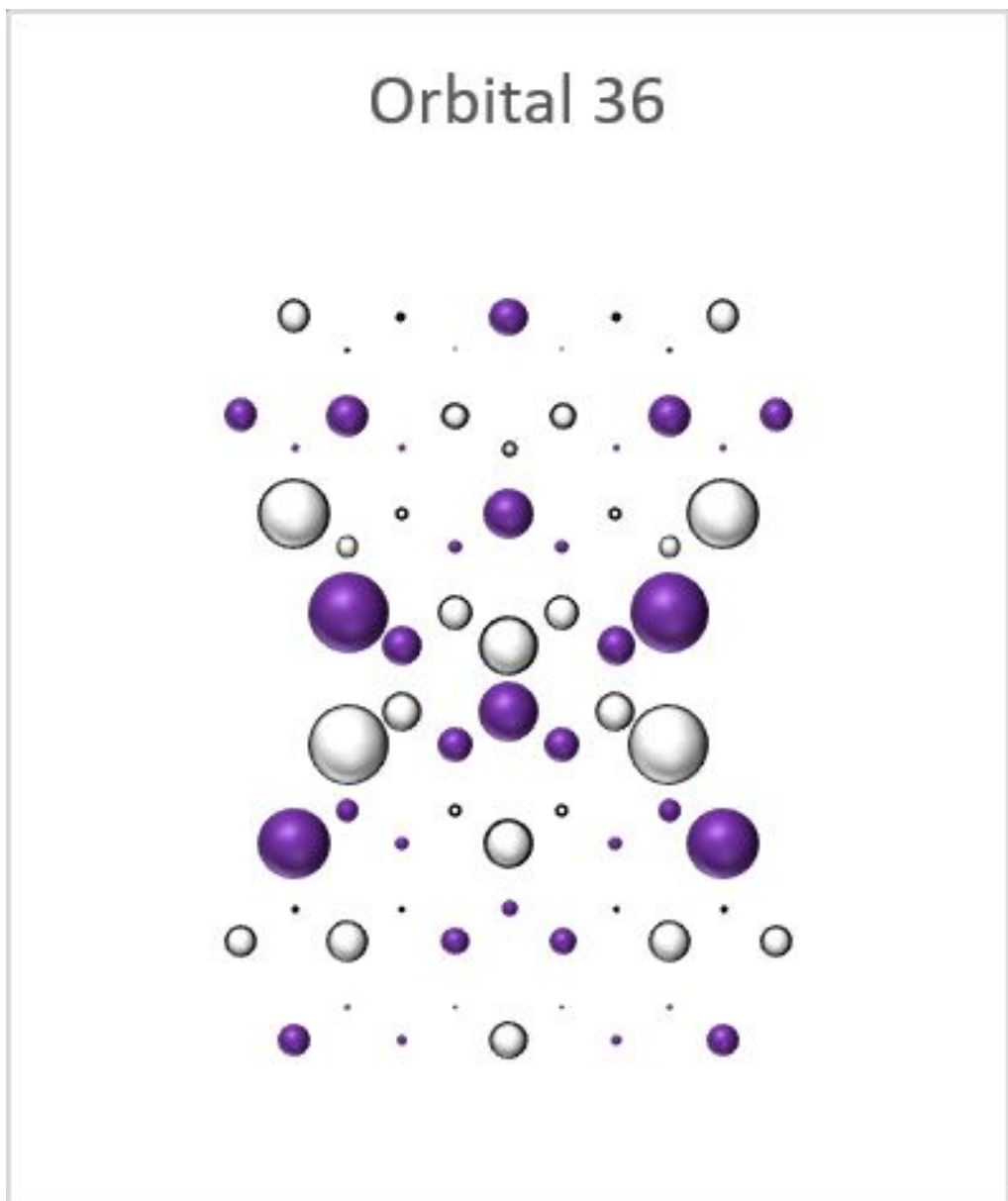
Orbital 36

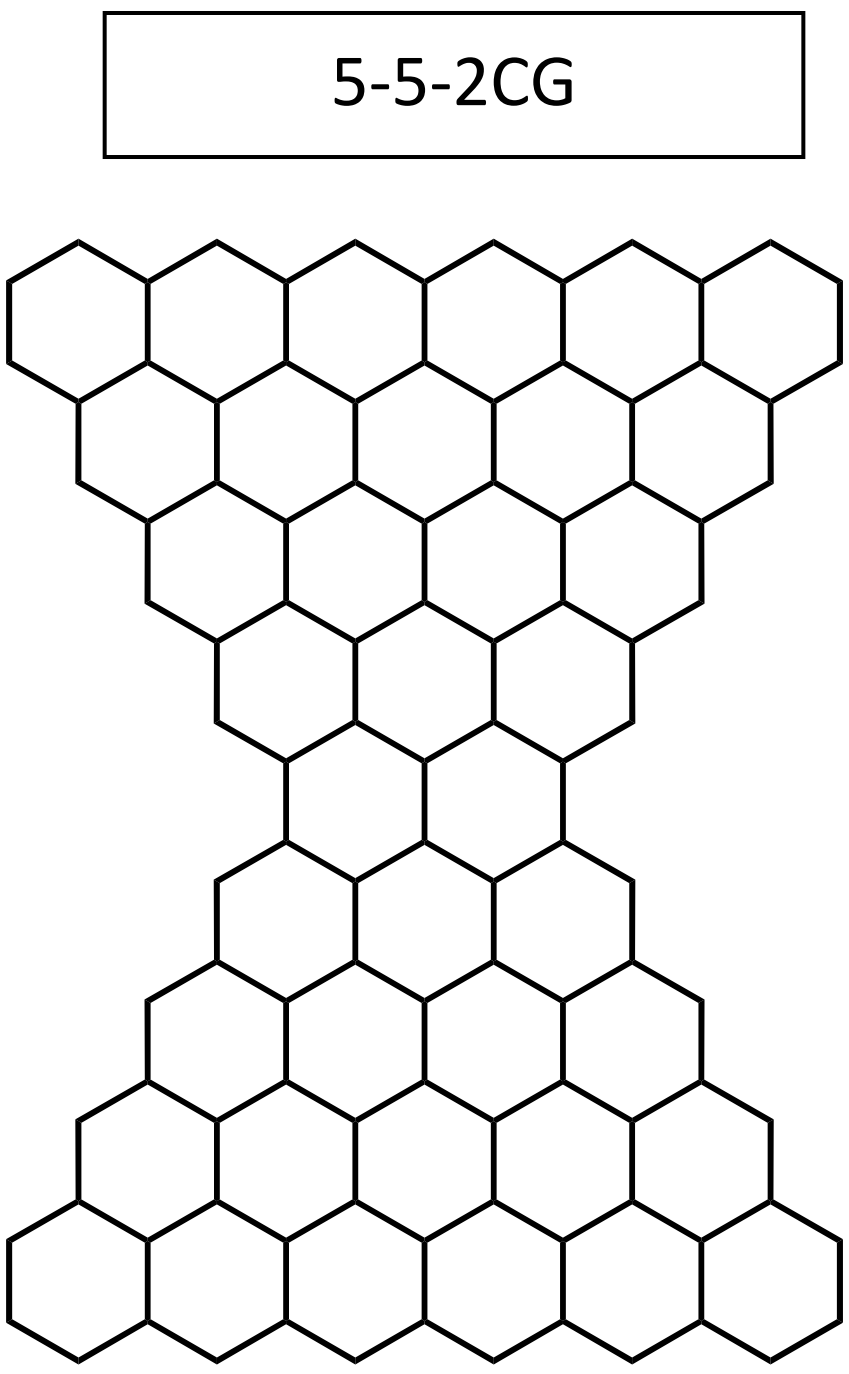
5-5-2CG

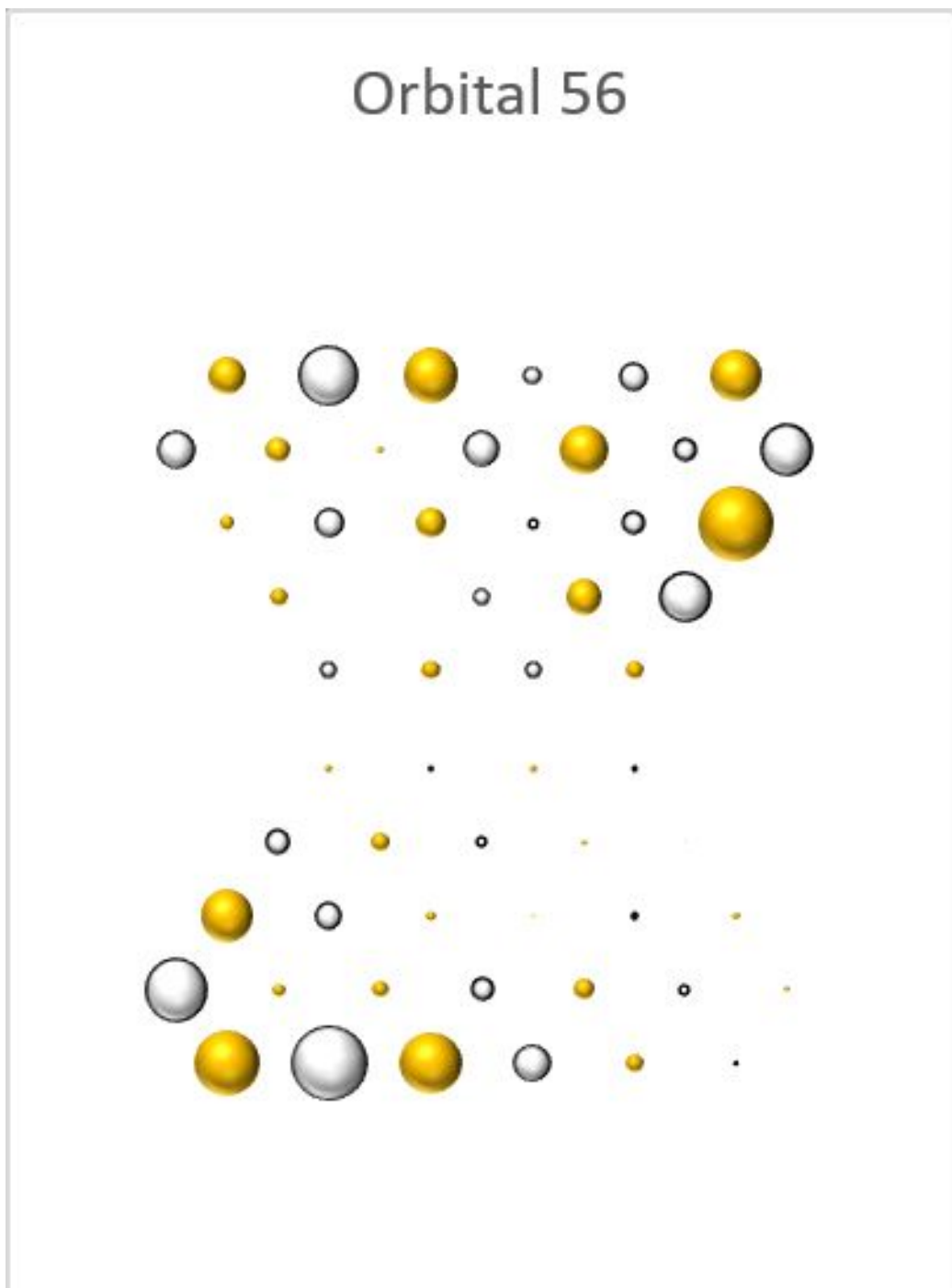
Orbital 56

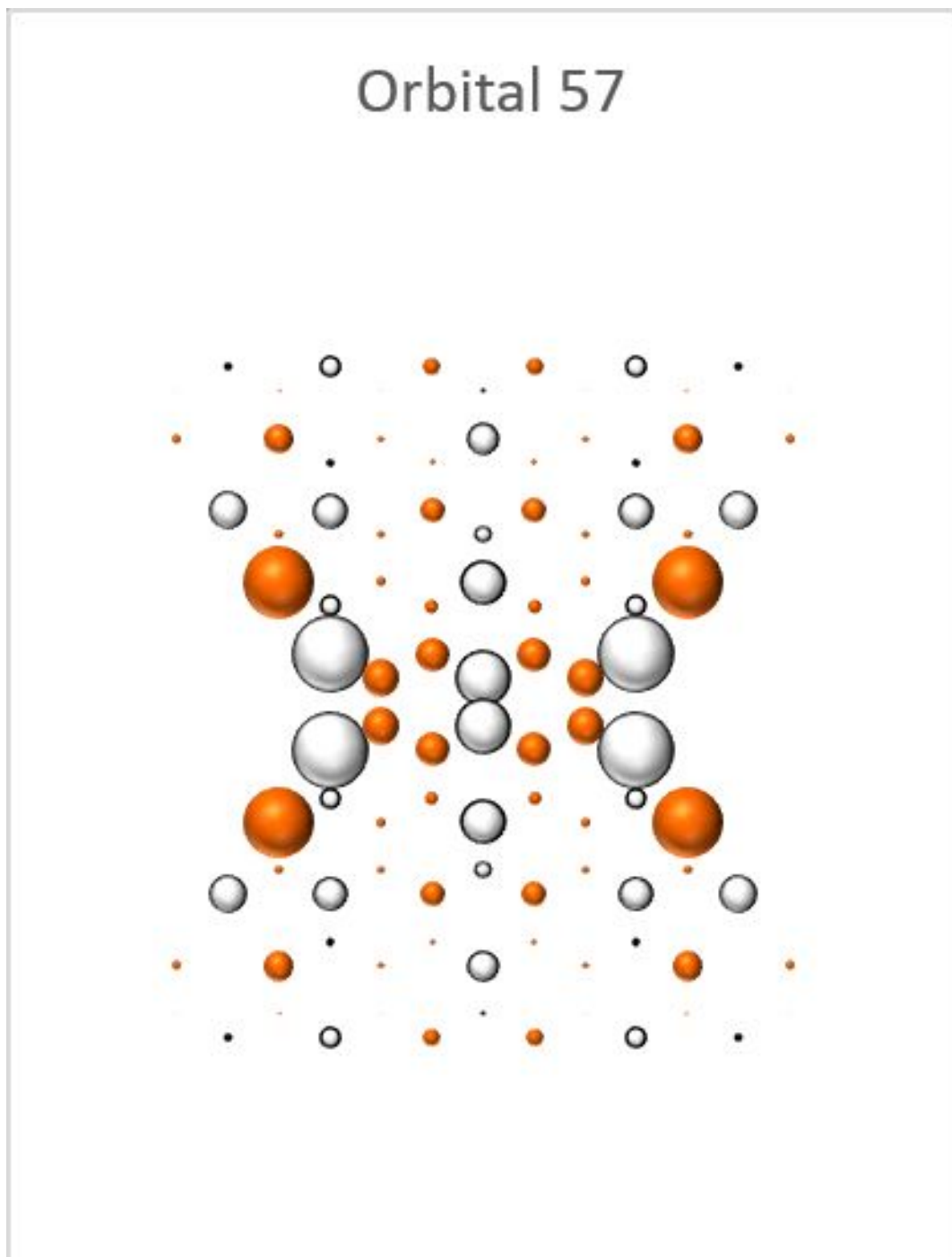
Orbital 57

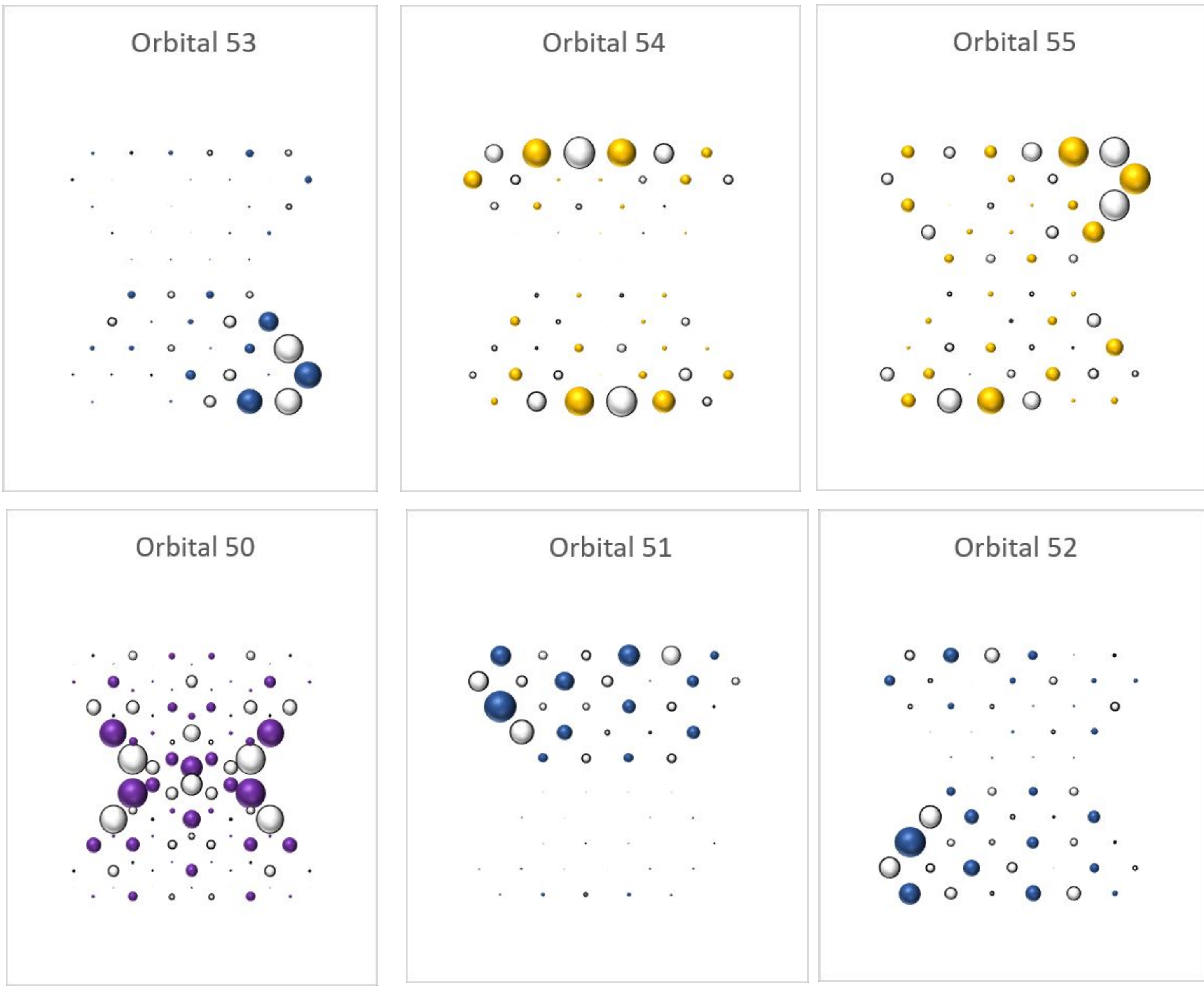

Orbital 53
Orbital 54
Orbital 55
Orbital 50
Orbital 51
Orbital 52

3-3-3CG

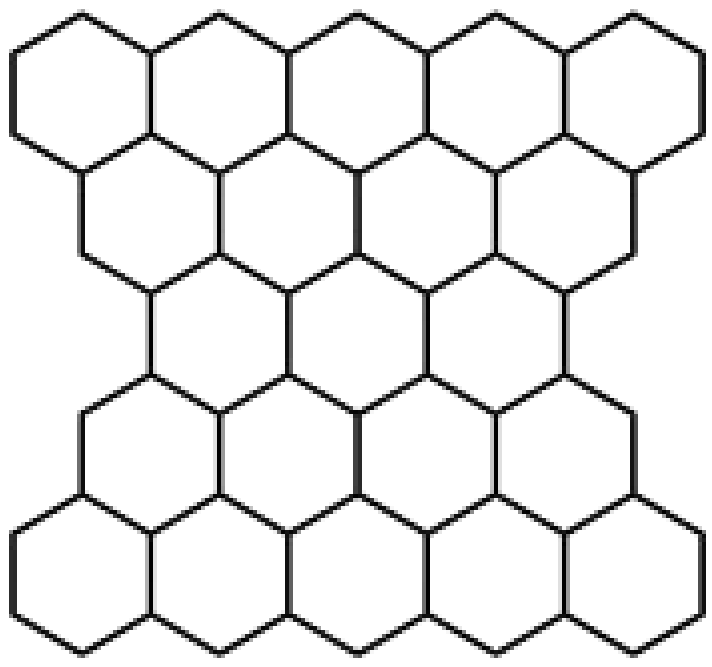

Orbital 32

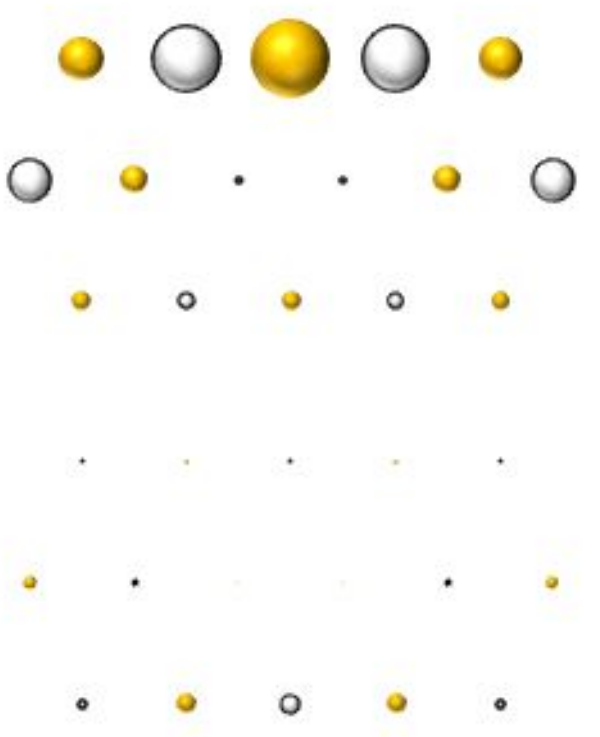

Orbital 33

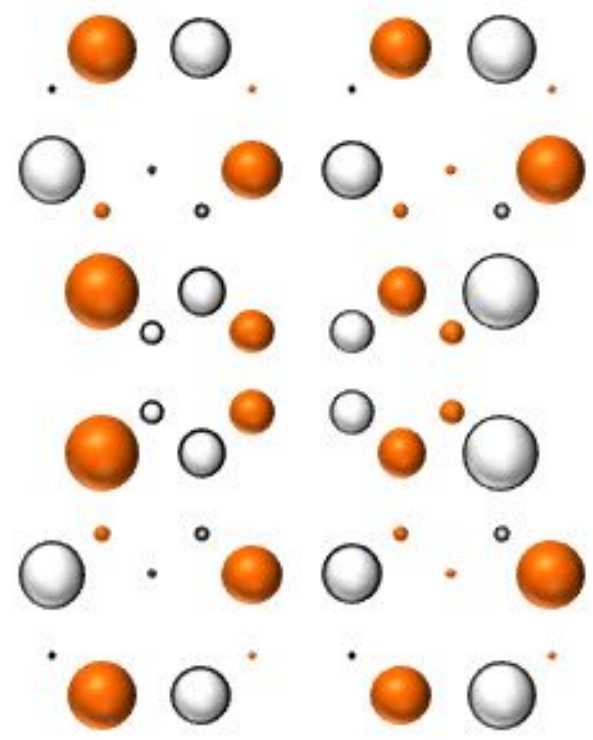

Orbital 30

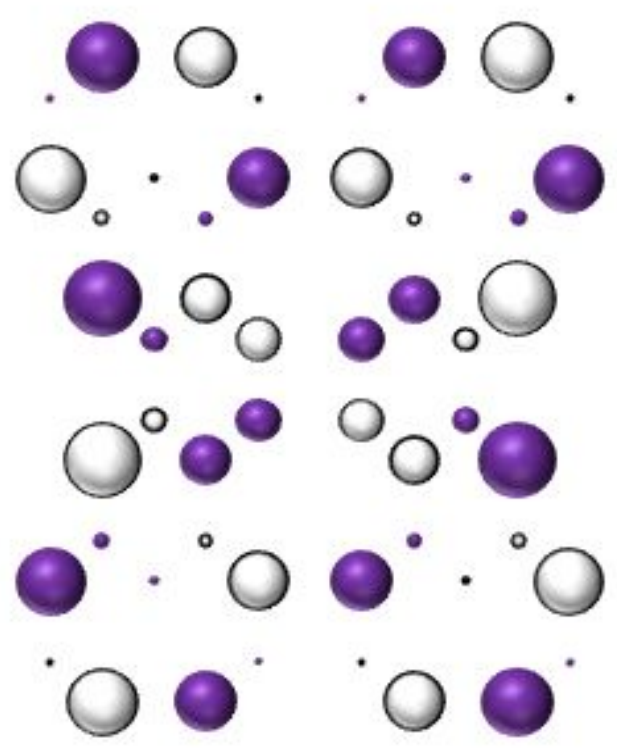

Orbital 31

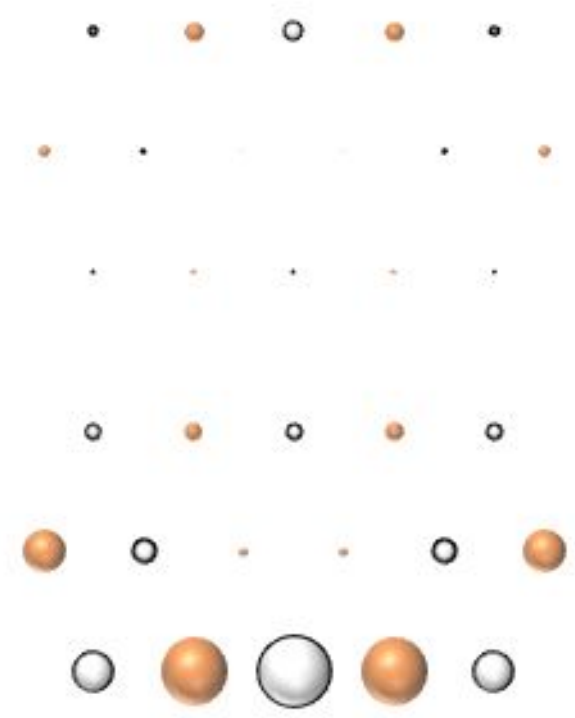

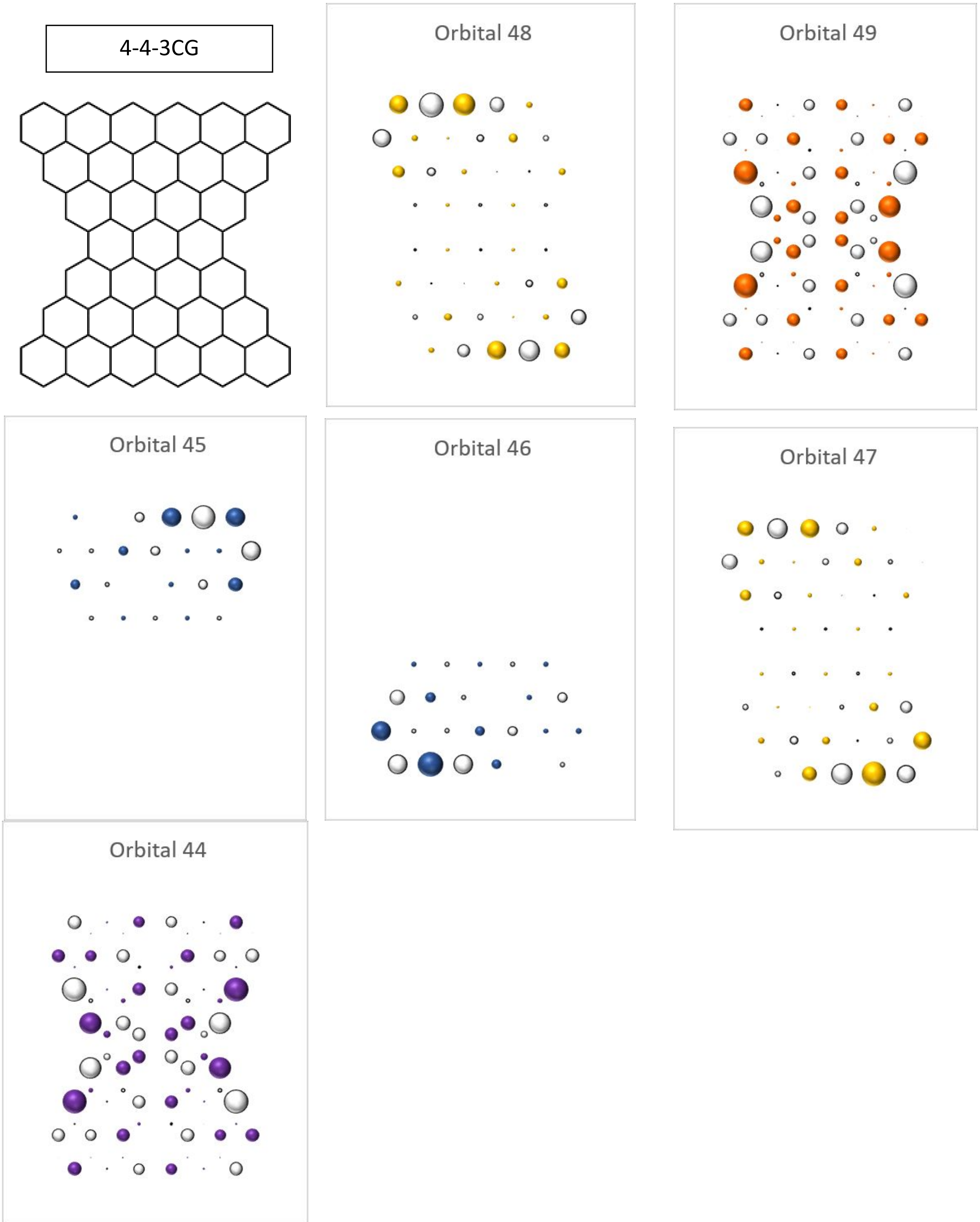
4-4-3CG
Orbital 48
Orbital 49
Orbital 45
Orbital 46
Orbital 47
Orbital 44

5-5-3CG

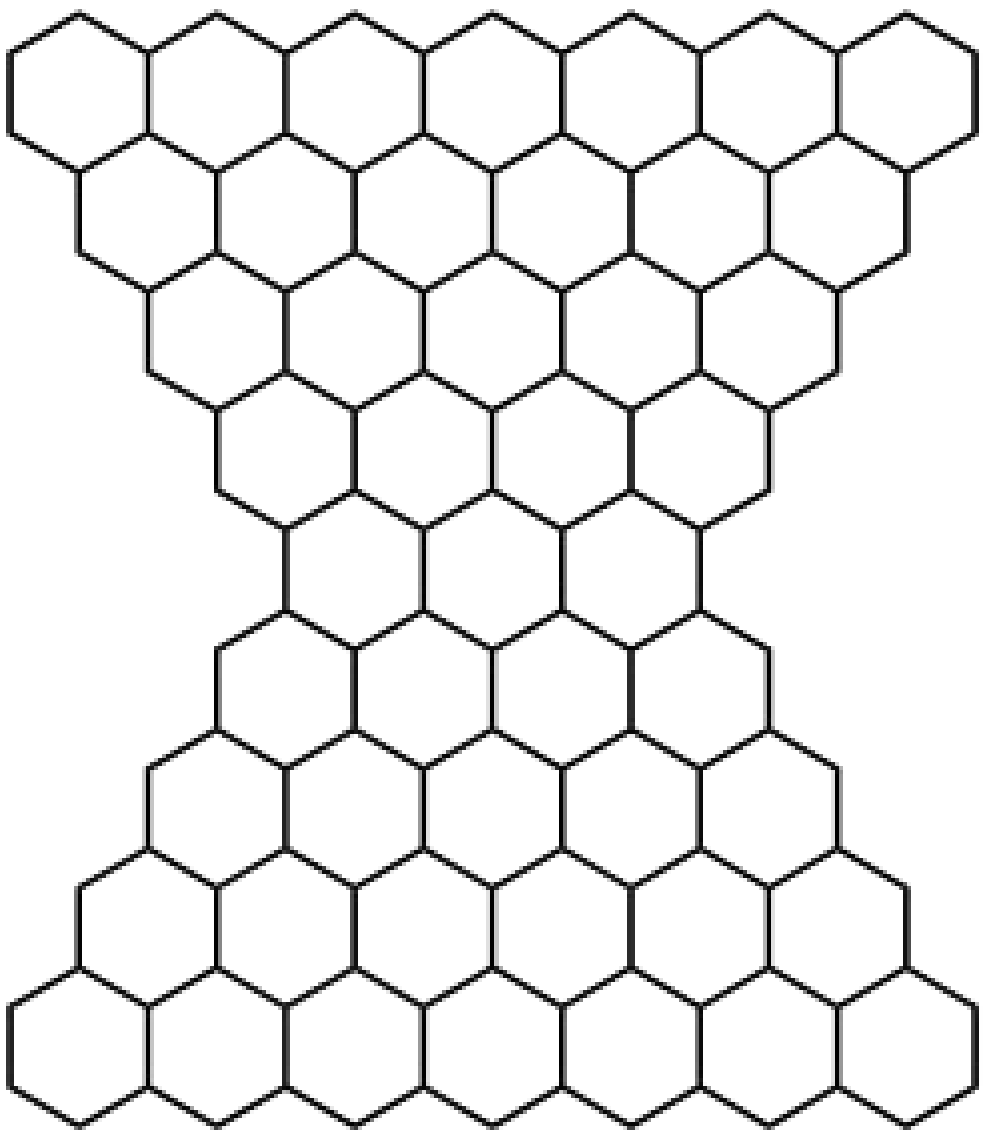

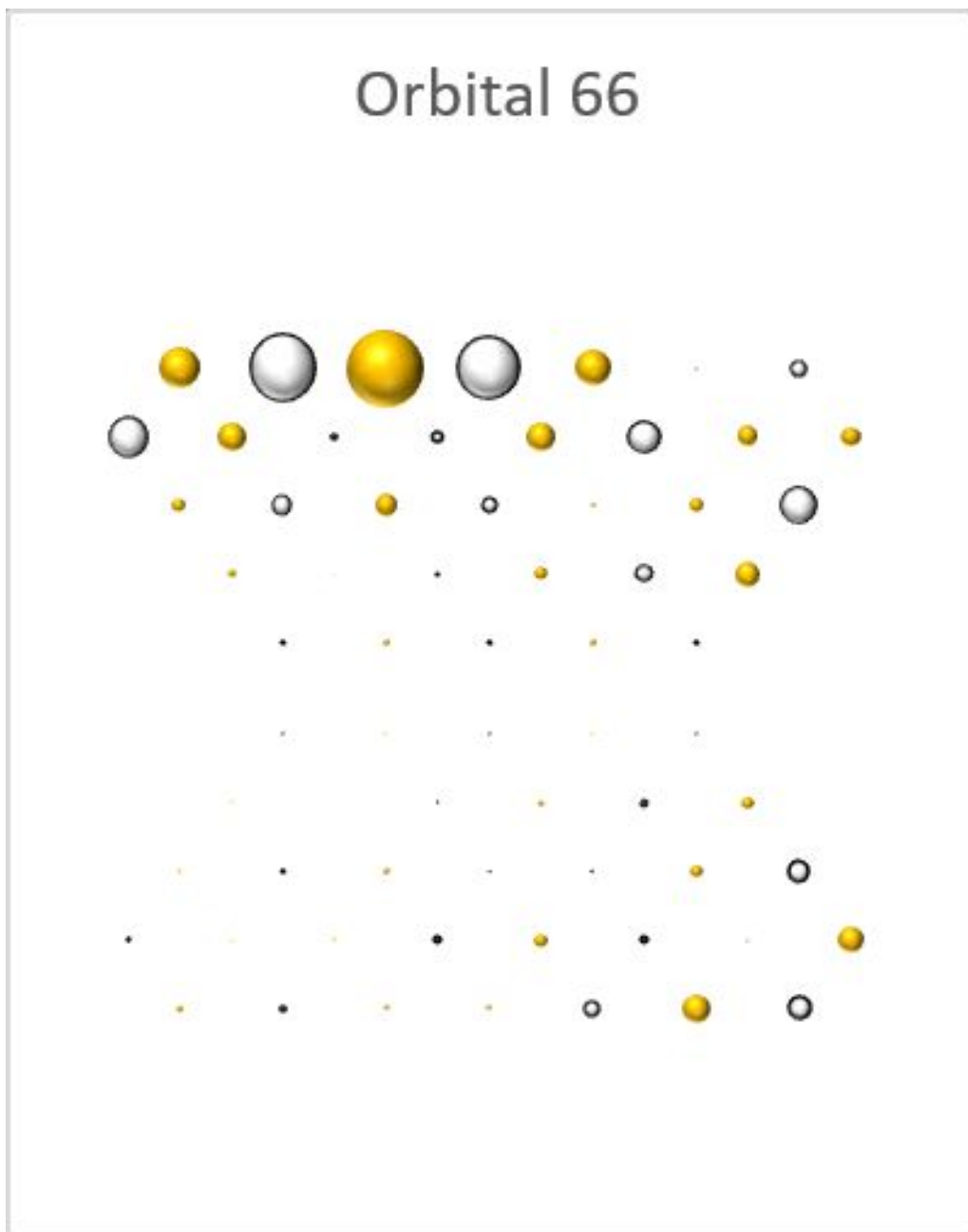

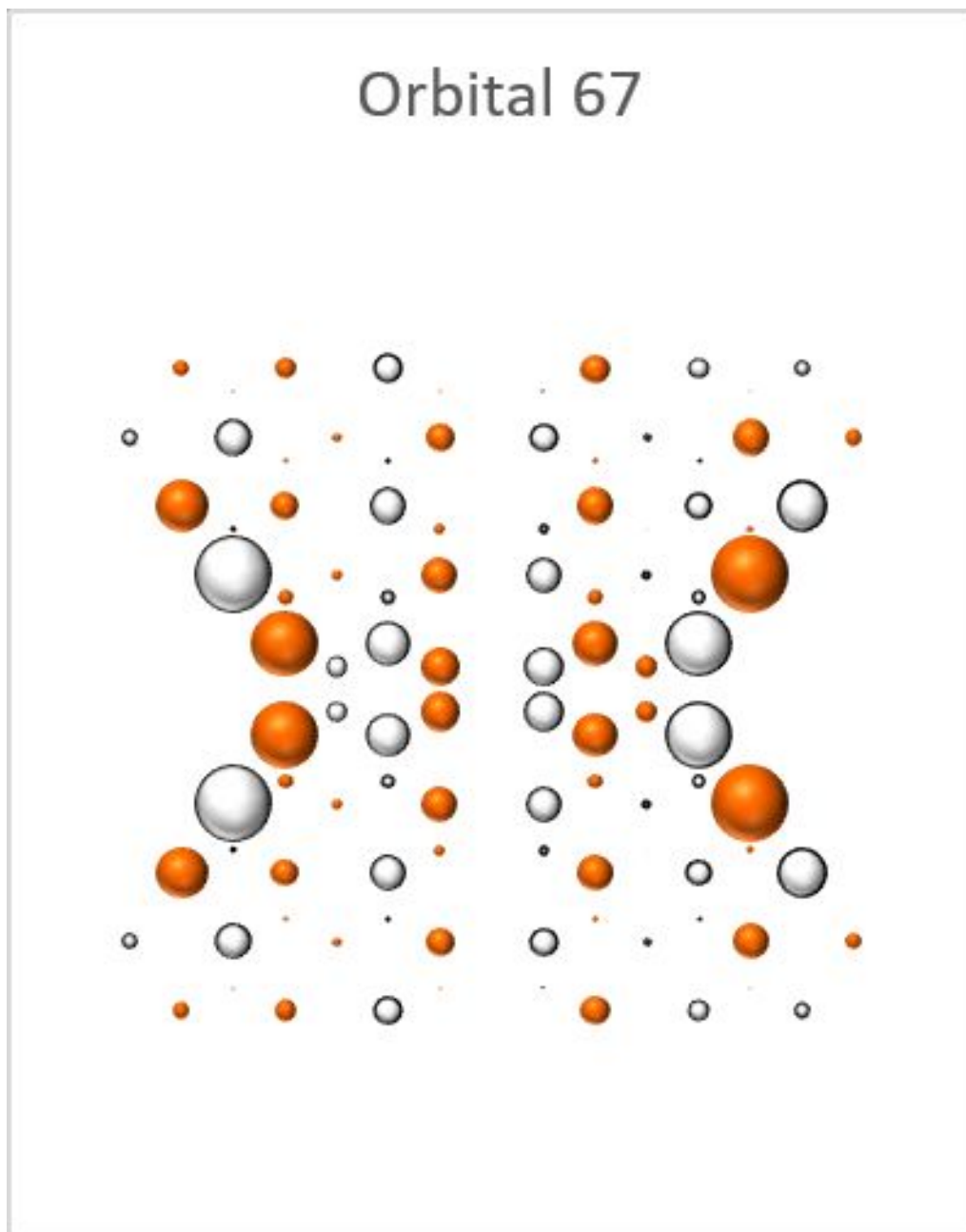

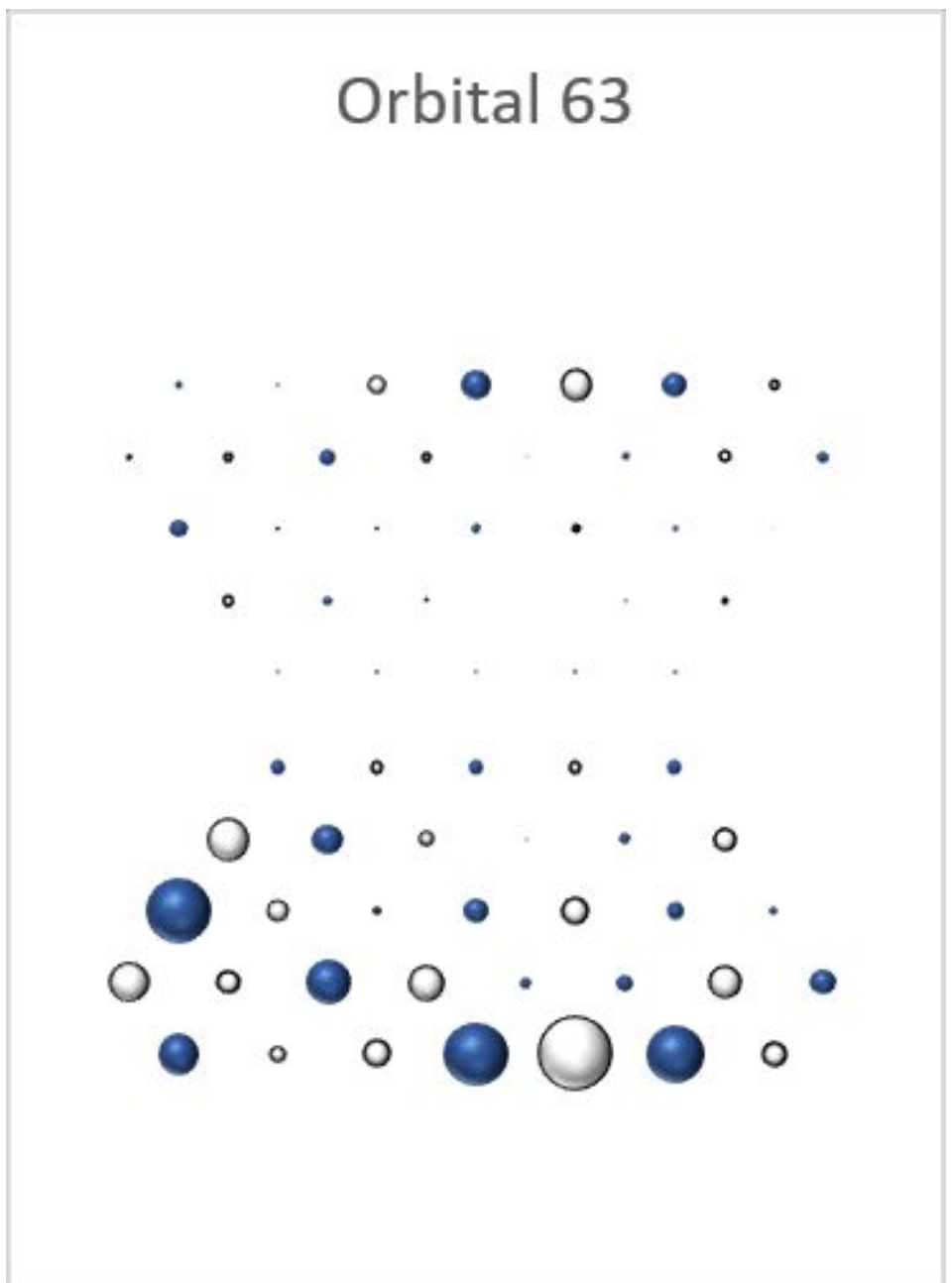

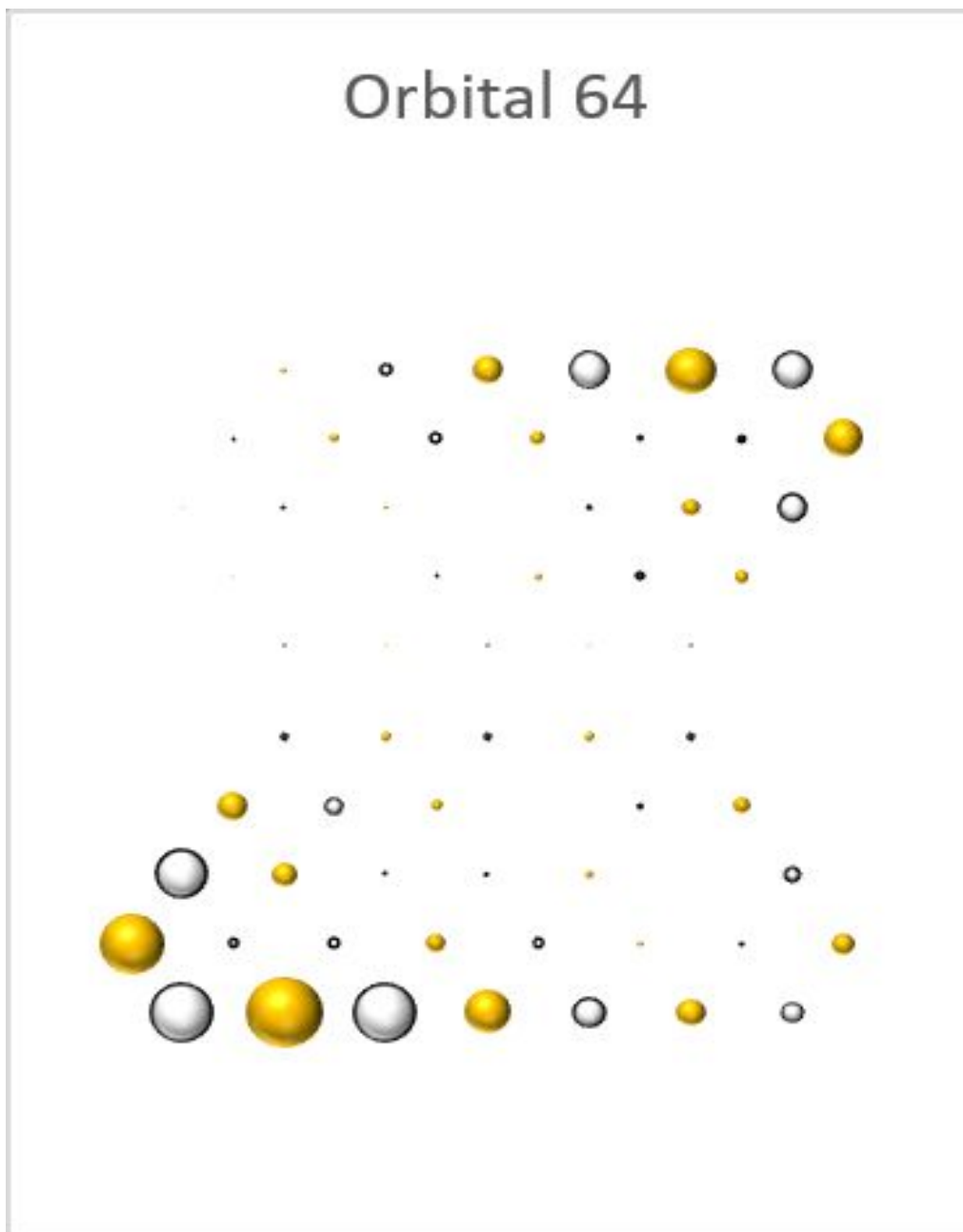

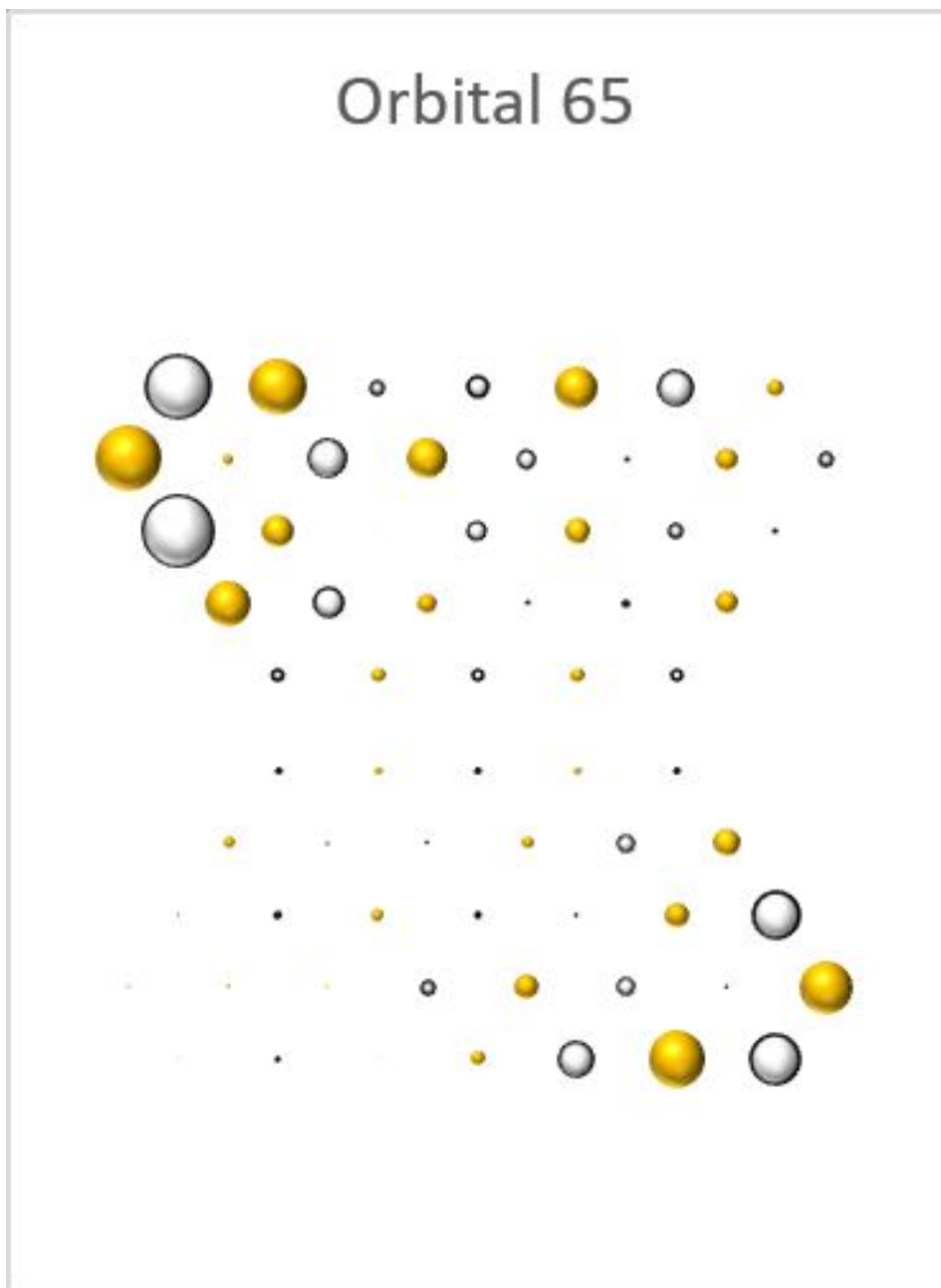

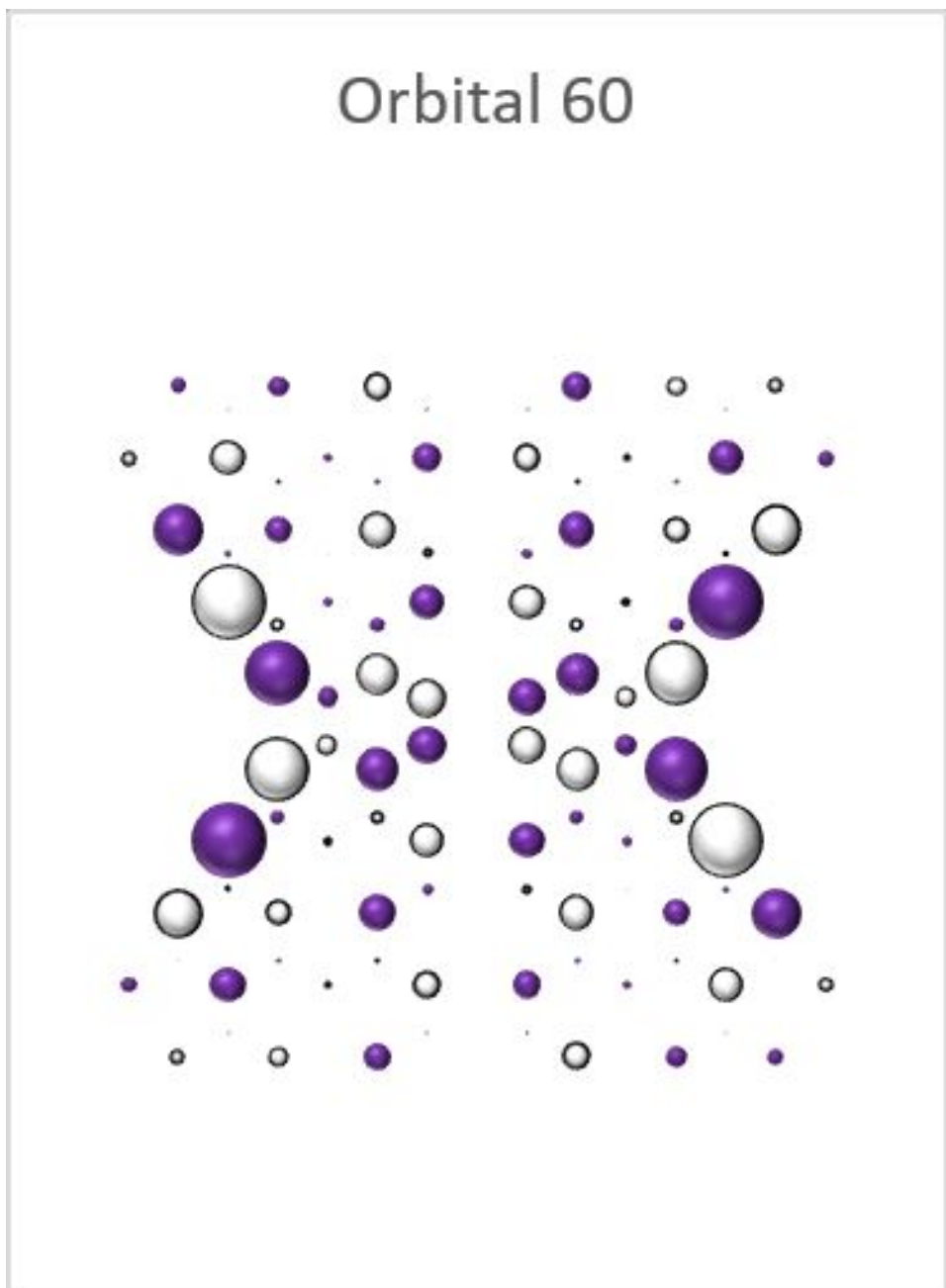

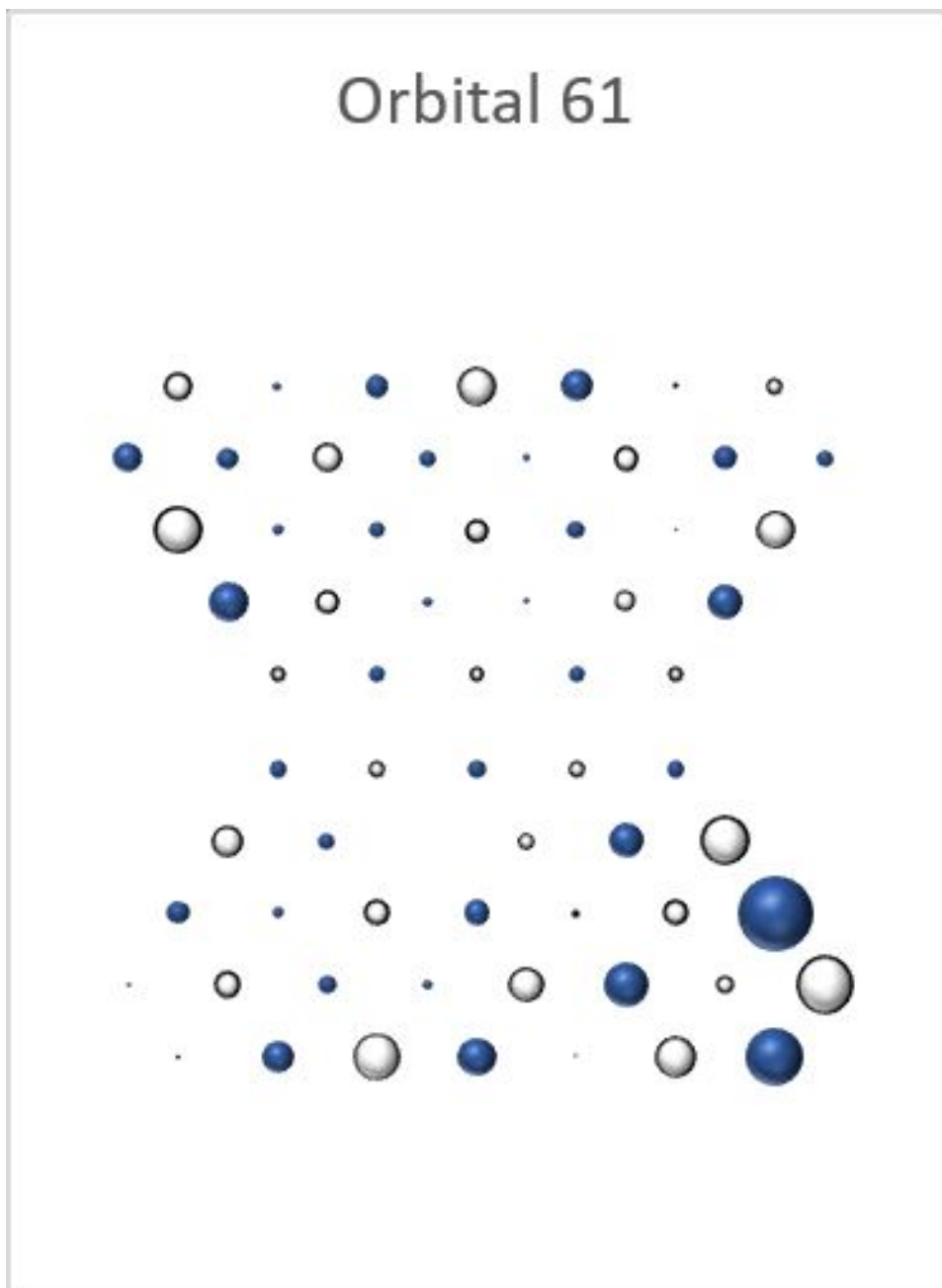

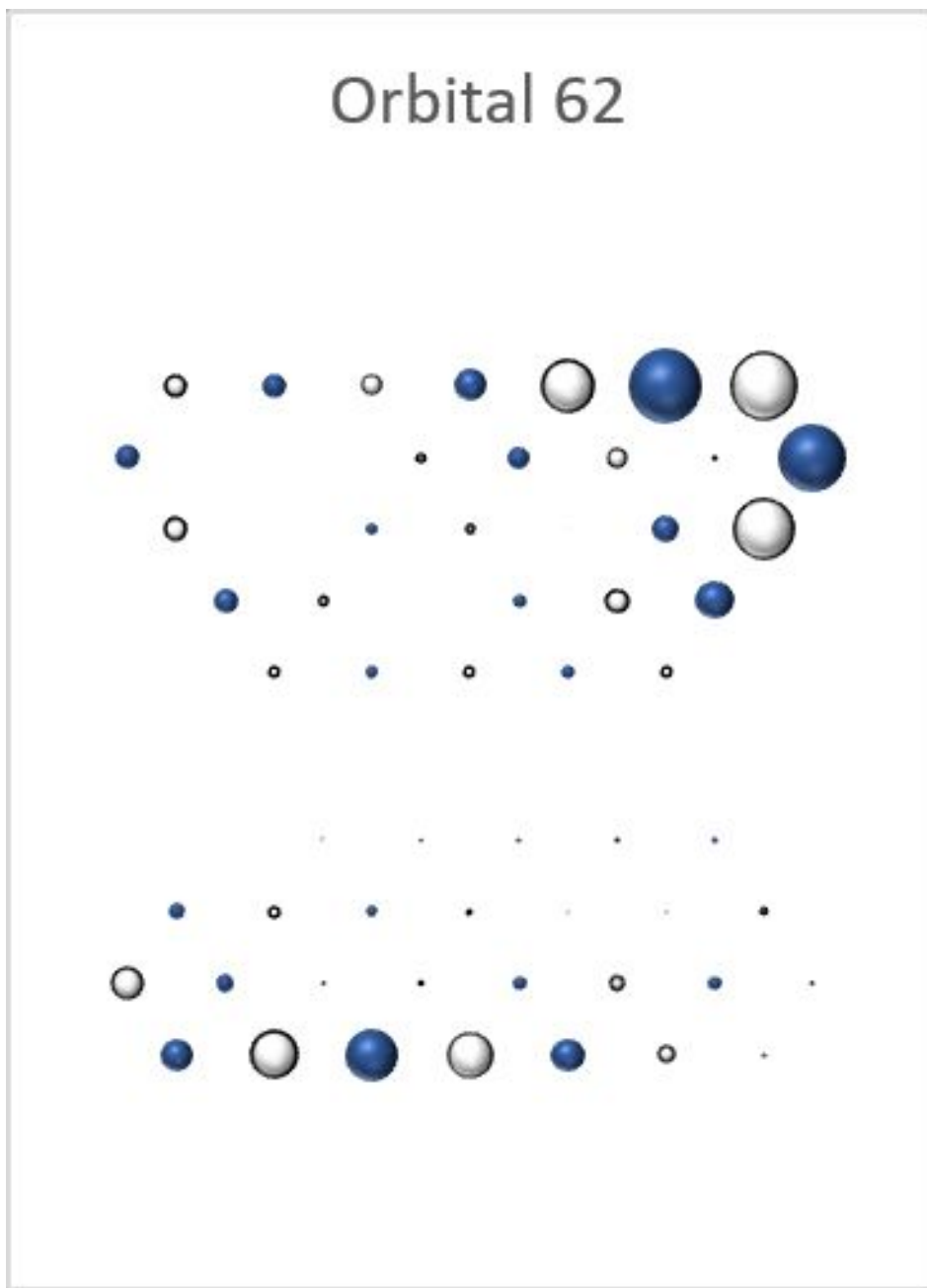

5-4-2CG

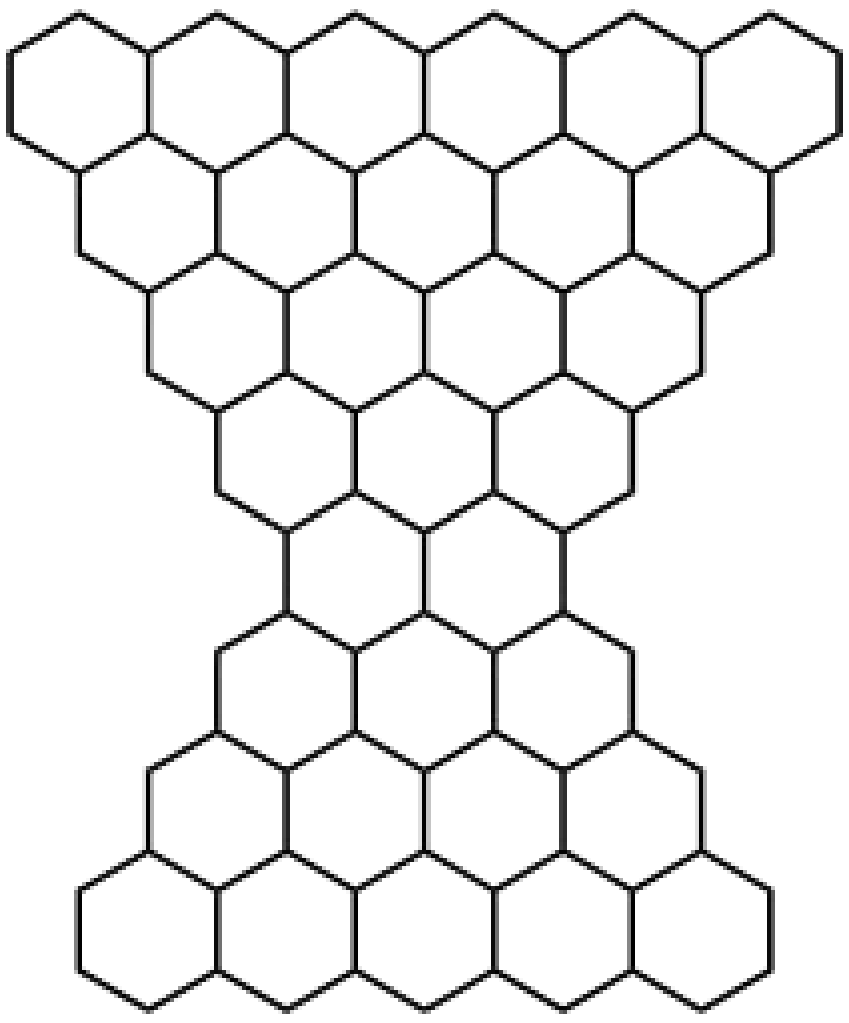

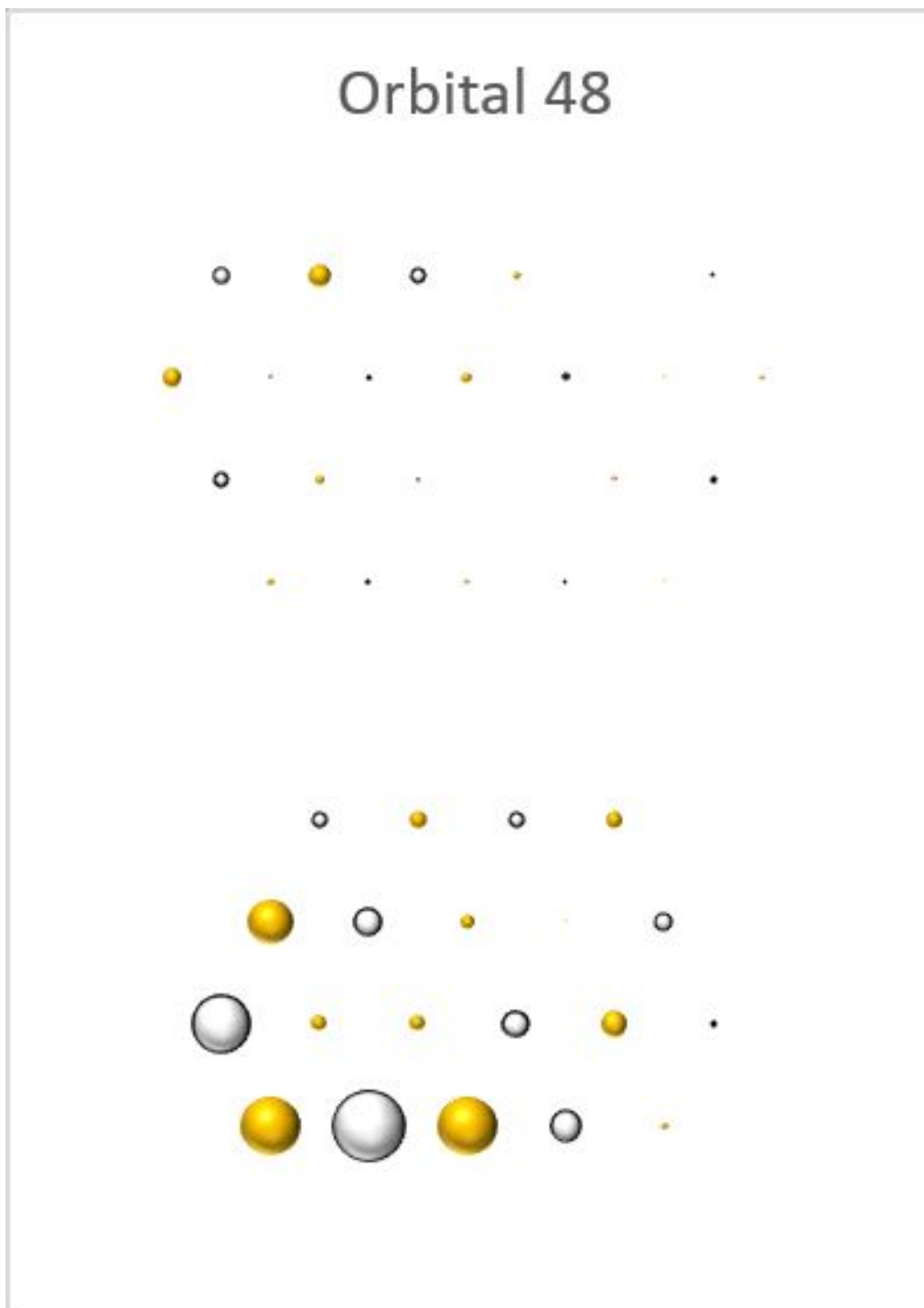

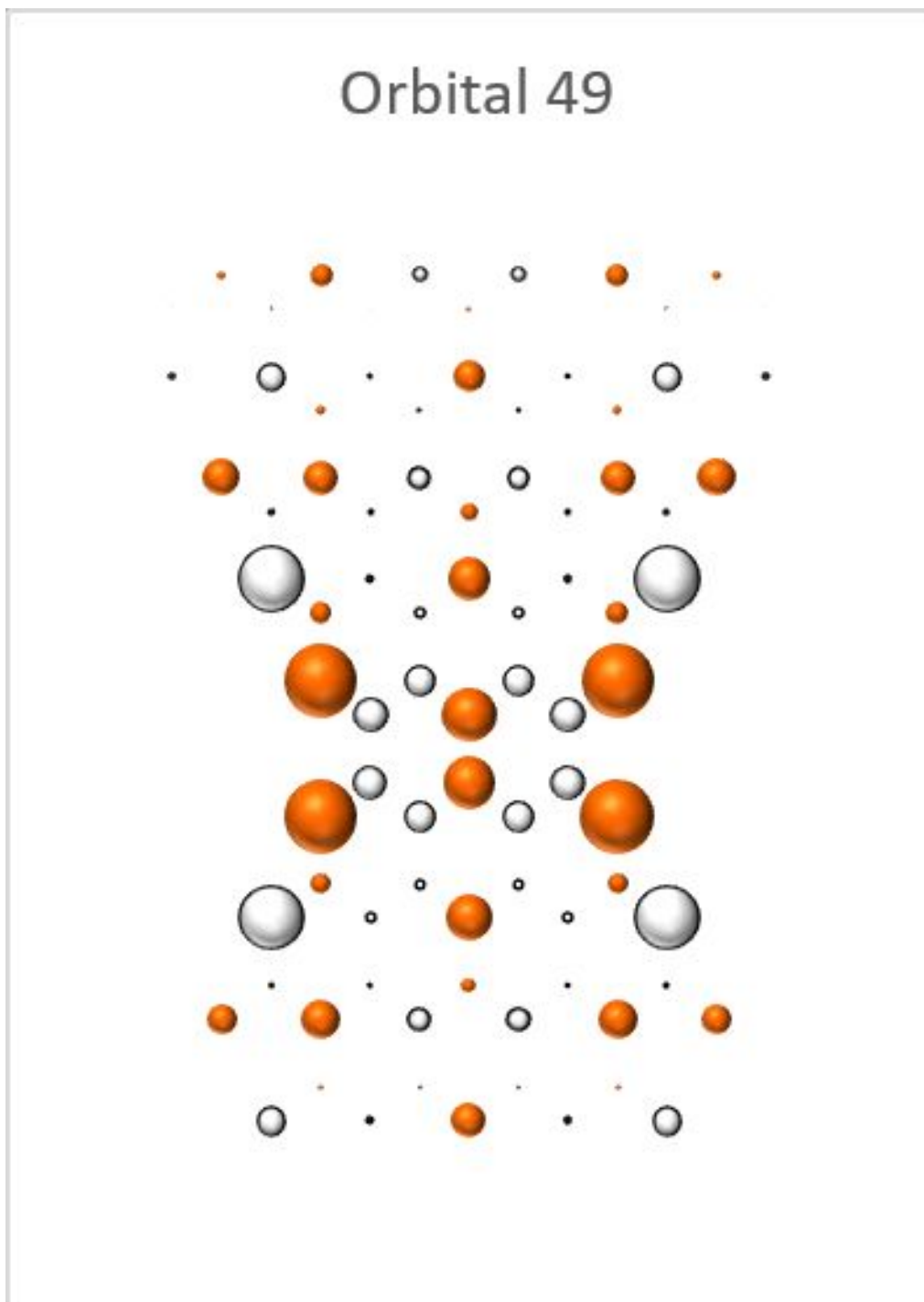

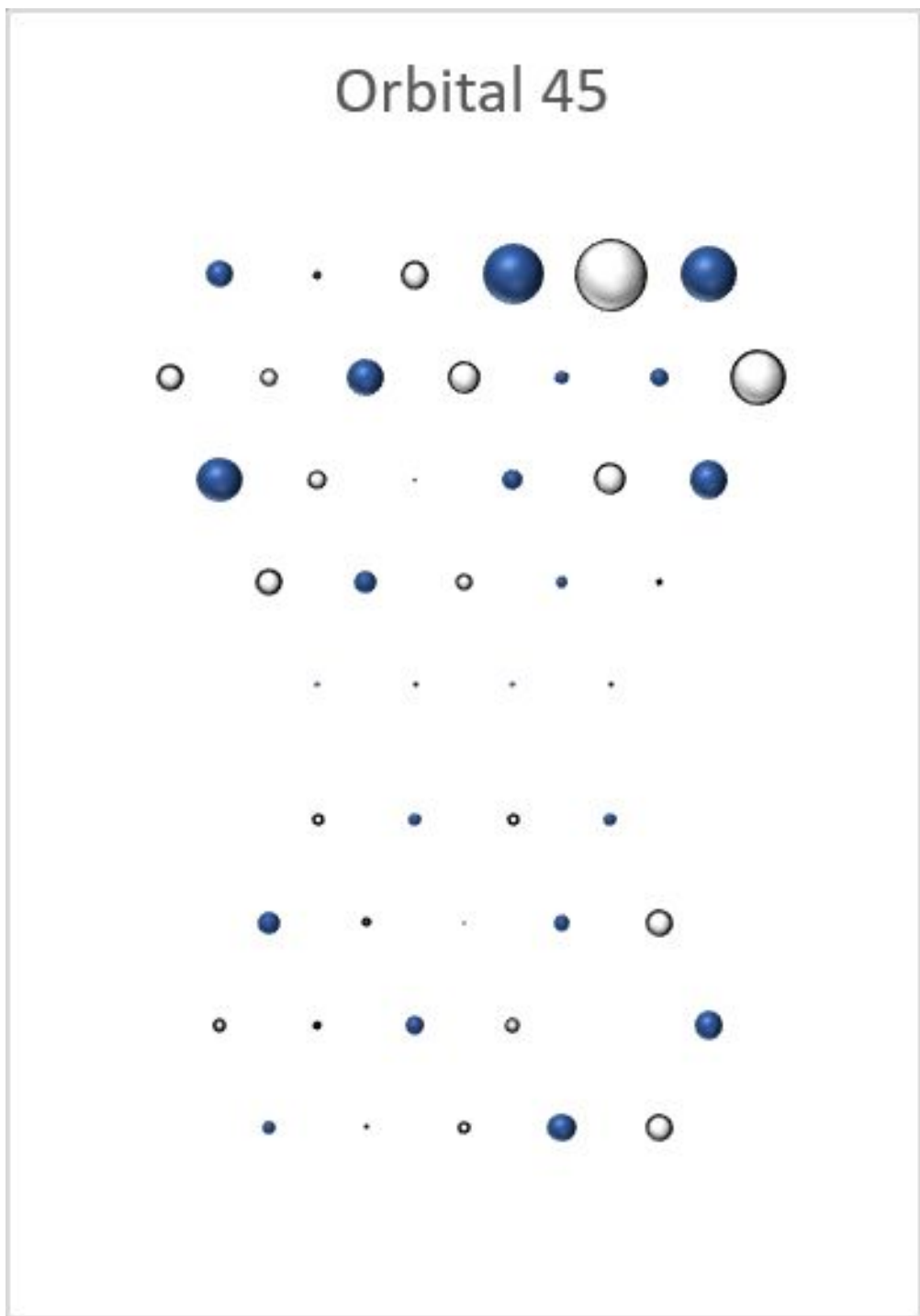
Orbital 45

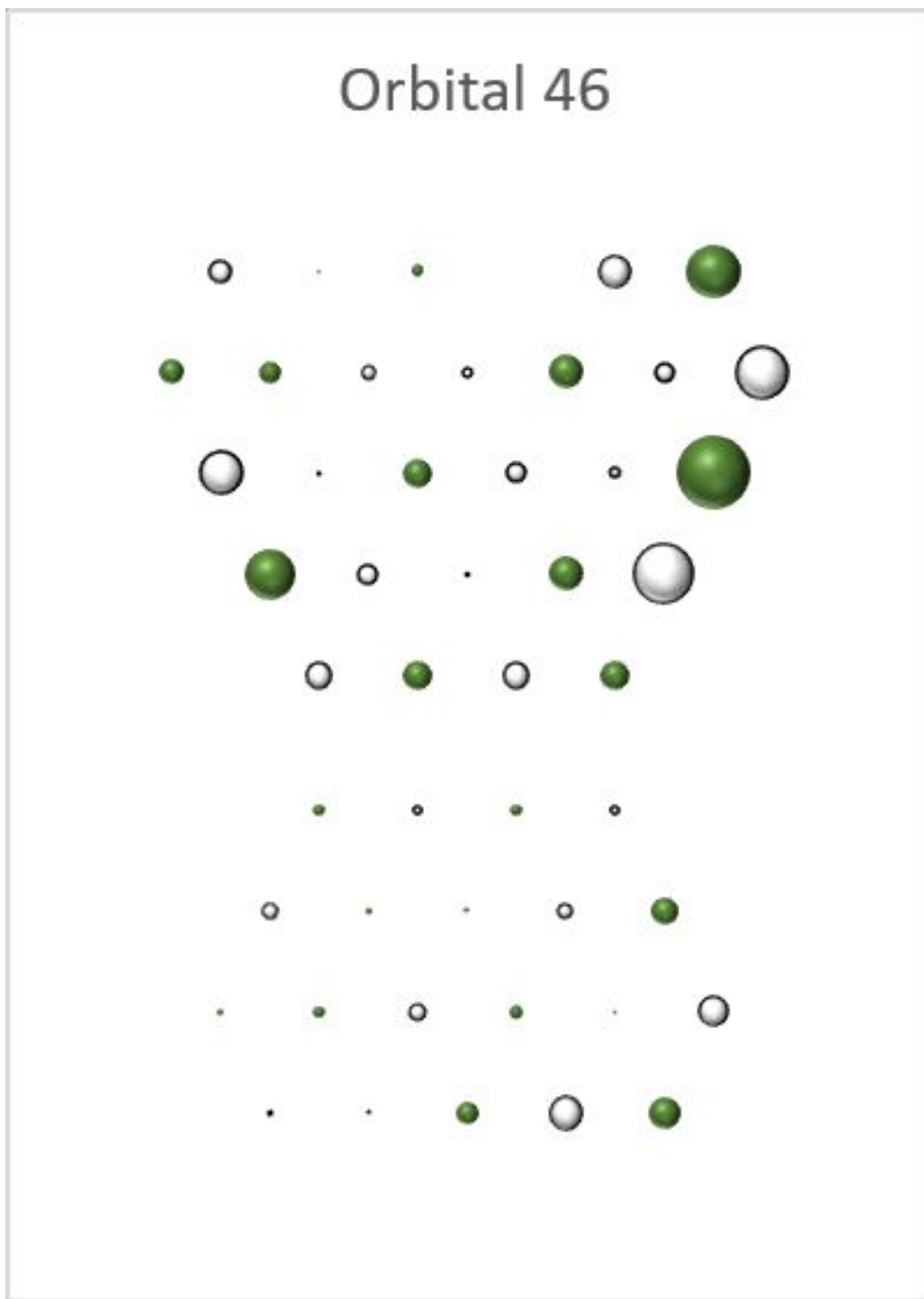
Orbital 46

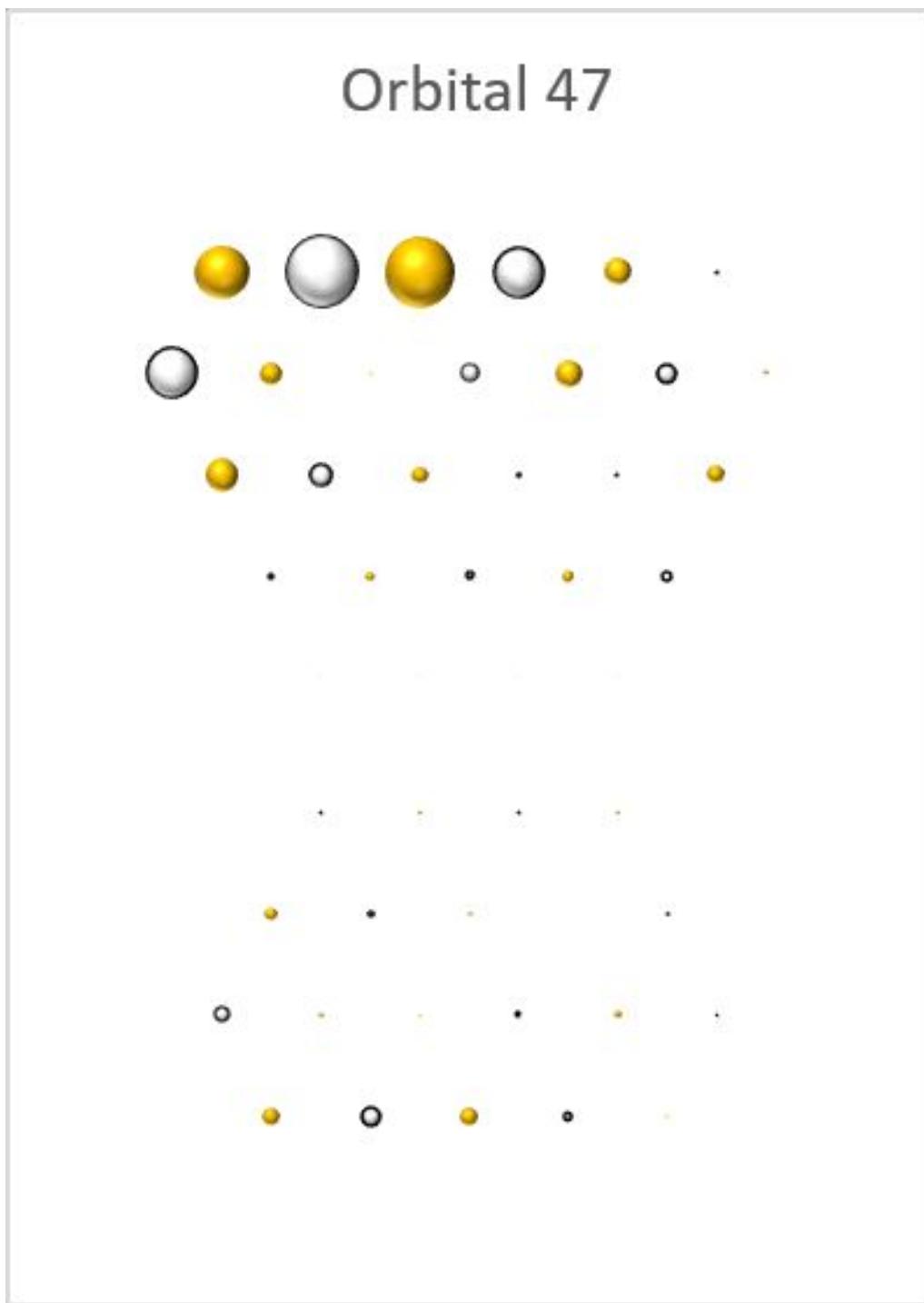
Orbital 47

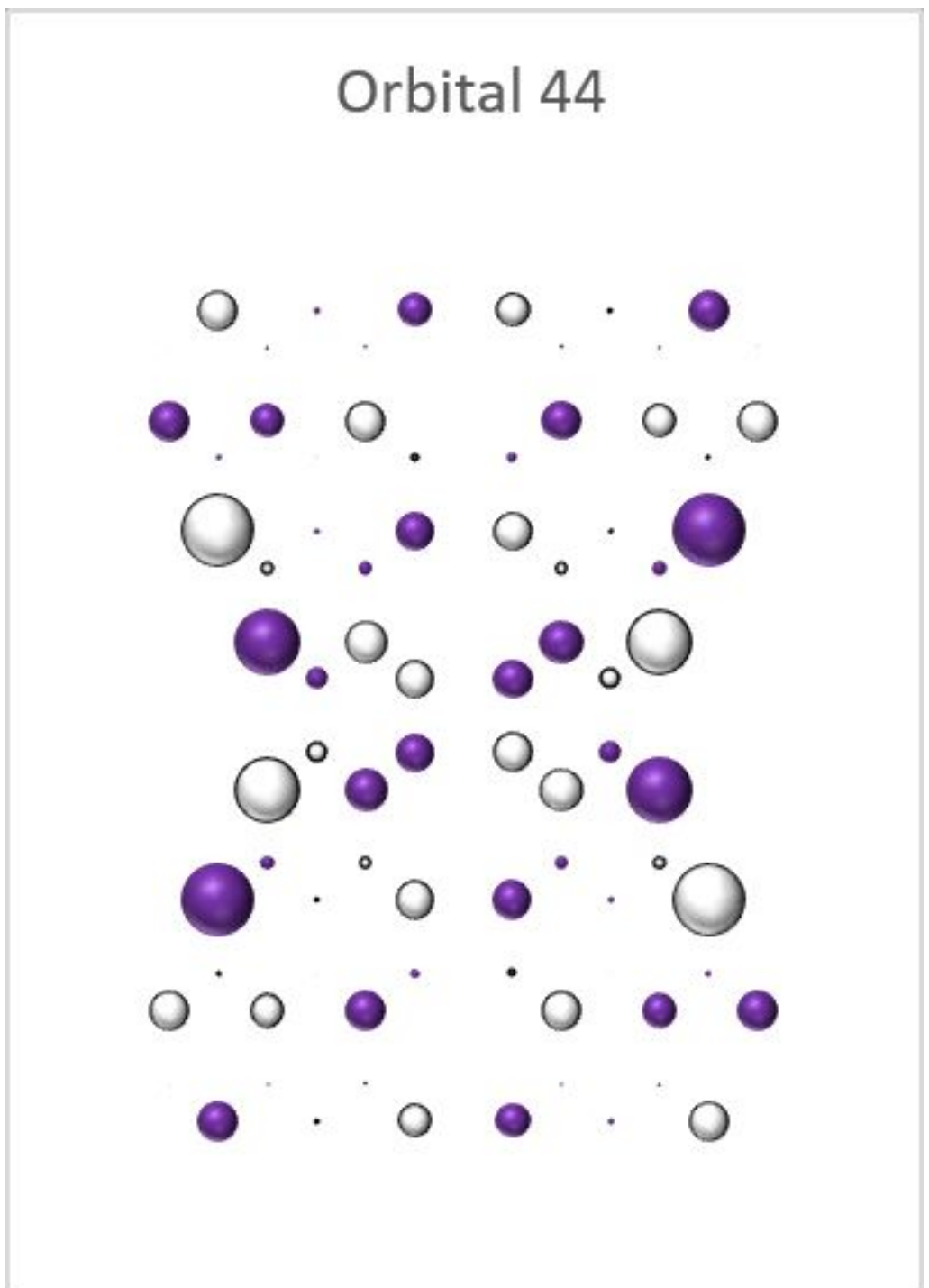
Orbital 44

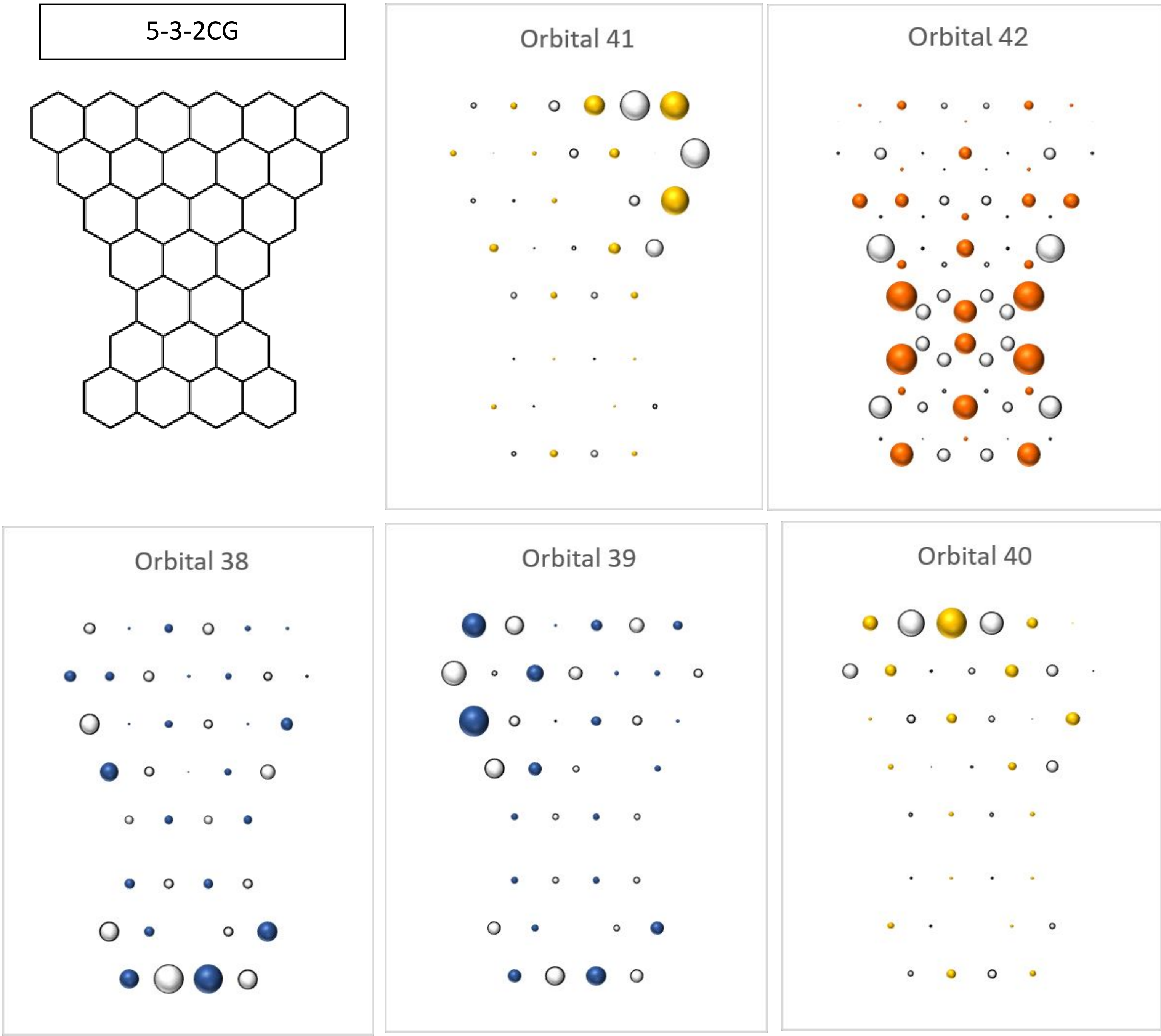
5-3-2CG
Orbital 41
Orbital 42
Orbital 38
Orbital 39
Orbital 40

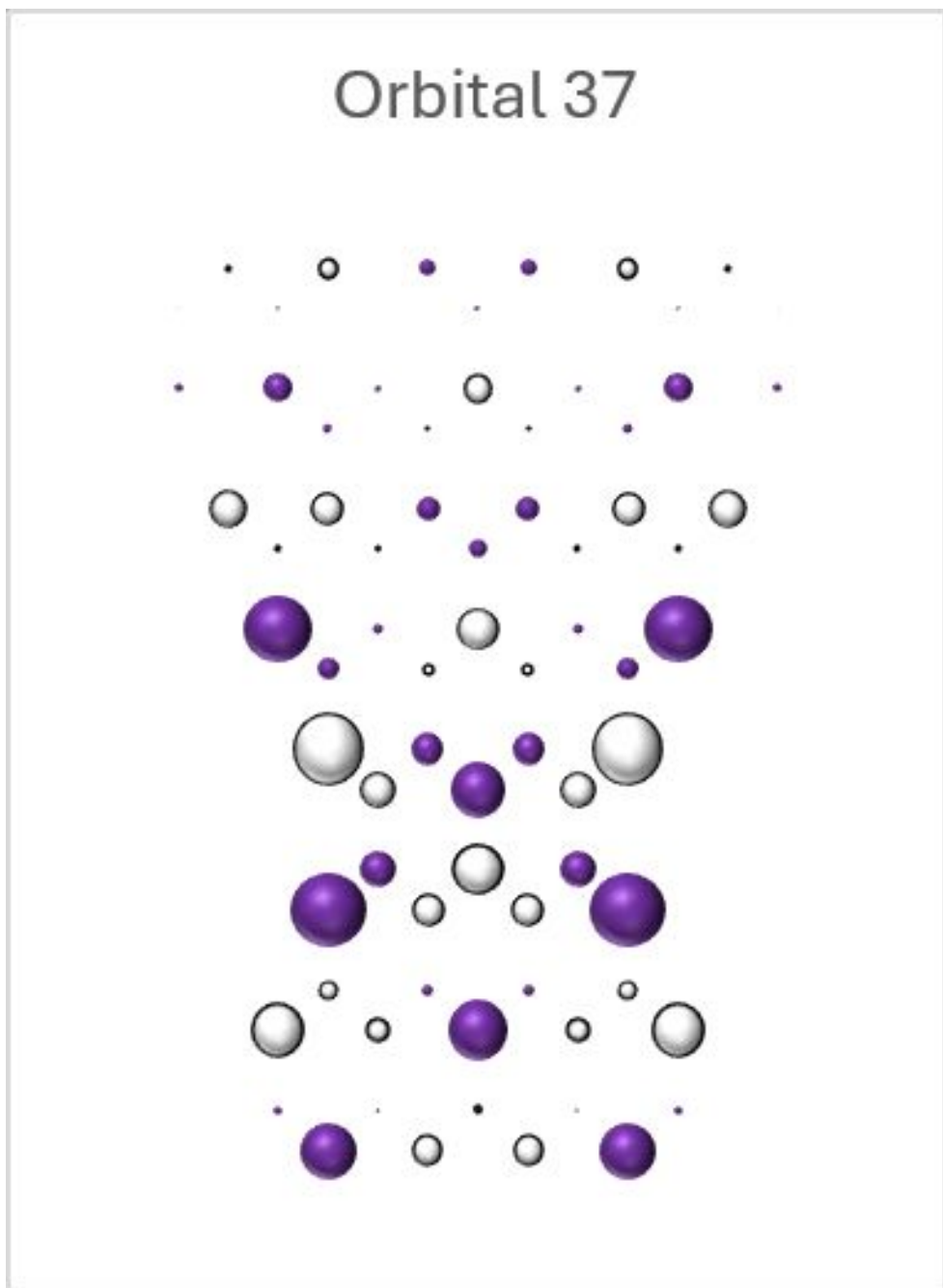

Pseudo-π vs *ab initio* calculations

Throughout this paper the pseudo-π level of approximation has been used to calculate the majority of the non-Hückel current maps. The following four diagrams — all pertaining to the neutral species — show *ipso-centric* current maps calculated using the program SYSMO.[19] The first map is at the pseudo-π level of approximation where only the cartesian coordinates of the carbon atoms are required. The second map shows the π-orbital contribution to the calculated ring current for a full ab-initio calculation employing 6-31G* basis set, and hence does include hydrogen atoms.

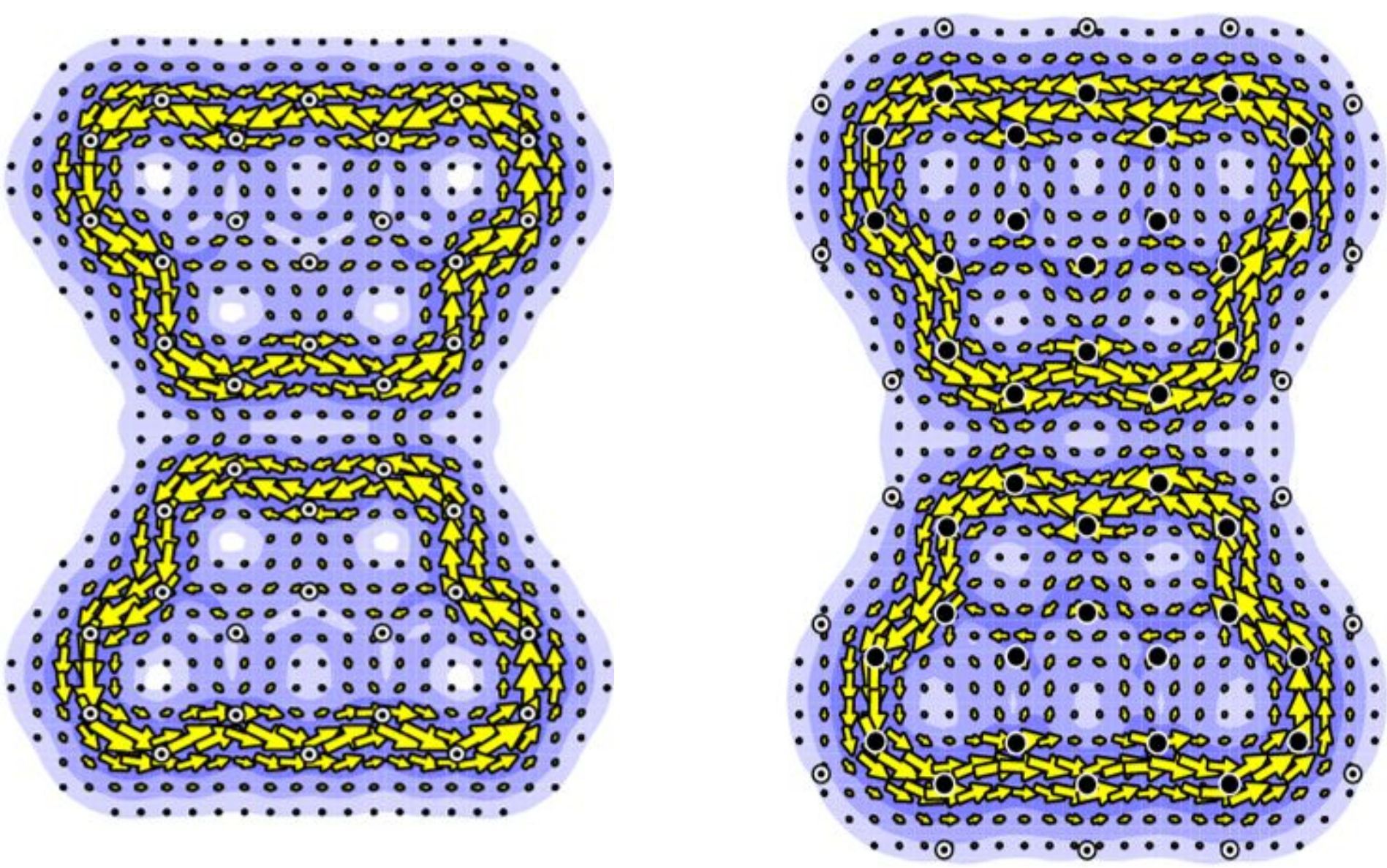

Pseudo-π

Figure S22.

Ab initio π framework contribution

Figure S23.

As can be seen from these maps, the calculated ring currents for the two levels of approximation (pseudo-π and ab initio) are almost indistinguishable. With the ab initio calculation it is possible to calculate the contribution from the σ-orbital framework and add this to the π-framework to give the total ab initio current map, as shown below:

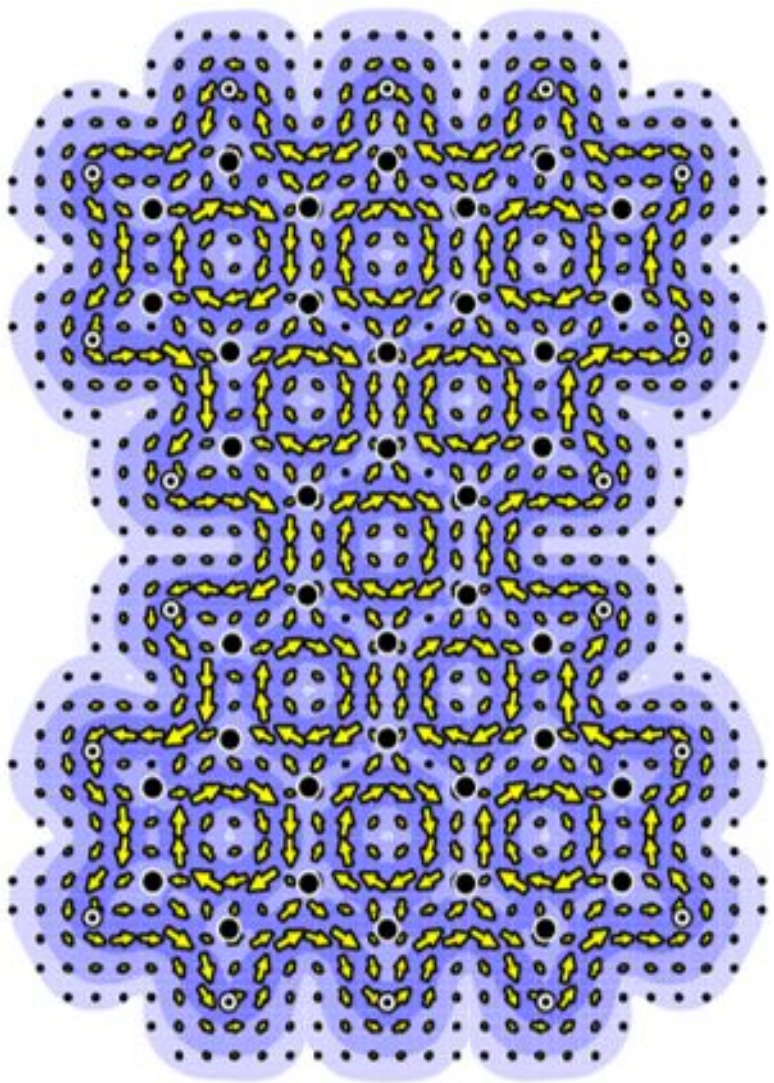

Ab initio σ framework contribution

Figure S24.

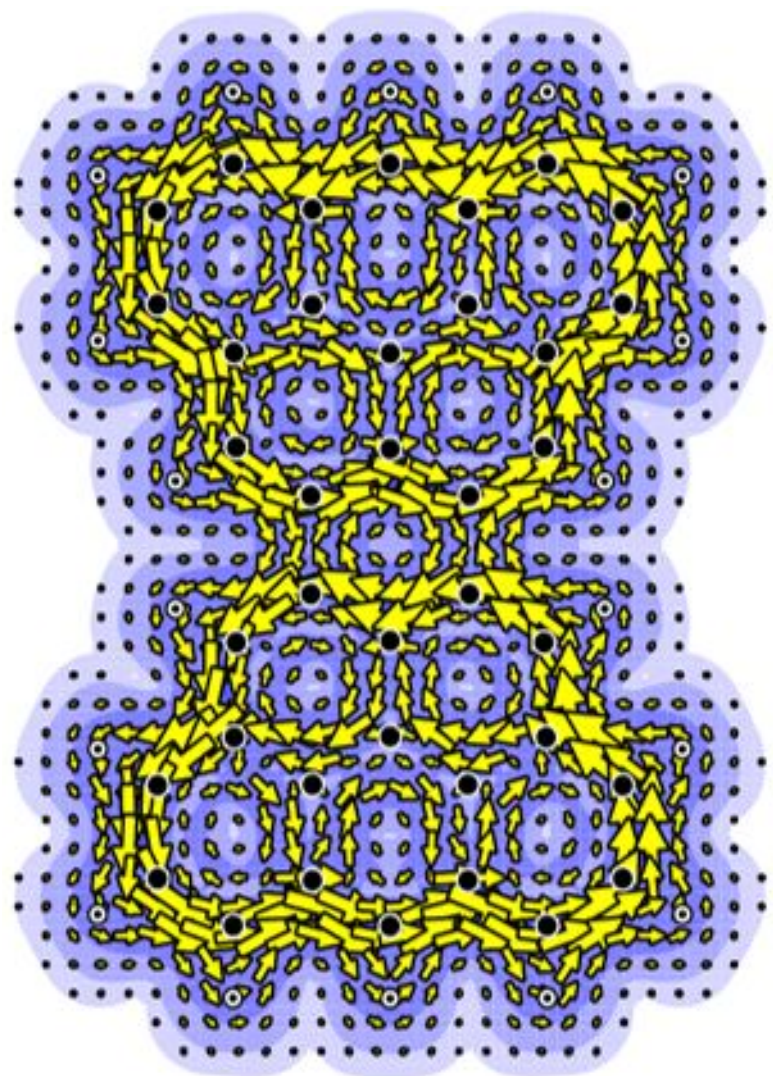

Total ab initio current map

Figure S25.

Whilst the total ab initio current map looks a little more complicated than the ab initio π-framework map, it can be seen that the σ-framework supports ring currents that are weaker and are localized around the hexagonal carbon framework. However, in essence, these diatropic currents flow in the same direction as those in the π-framework.

Cartesian Coordinates

Table S15. The following tables list the X and Y carbon-atom coordinates (in Ångstrom units) used for all calculations in this paper. All structures are flat, composed of hexagons with bond angles of 120°; hence there is no strain or deformation.

3-3-1 Coordinates	
X	Y
3.6582	3.6240
-3.6582	-3.6240
3.6582	-3.6240

4-4-1 Coordinates	
X	Y
-3.6360	4.8980
-3.6360	3.4990
-2.4241	2.7990

5-5-1 Coordinates	
X	Y
-3.6360	4.8980
-3.6360	3.4989
-2.4241	2.7989

-3.6582	3.6240
2.4494	2.8919
-2.4494	-2.8919
2.4494	-2.8919
-2.4494	2.8919
1.2207	3.5962
-1.2207	-3.5962
1.2207	-3.5962
-1.2207	3.5962
1.2249	5.0282
-1.2249	-5.0282
1.2249	-5.0282
-1.2249	5.0282
2.4651	5.7194
-2.4651	-5.7194
2.4651	-5.7194
-2.4651	5.7194
3.6655	5.0211
-3.6655	-5.0211
3.6655	-5.0211
-3.6655	5.0211
0.0000	2.8748
0.0000	-2.8748
0.0000	5.7174
0.0000	-5.7174
0.0000	1.4439
0.0000	-1.4439
1.2534	0.7402
-1.2534	-0.7402
1.2534	-0.7402
-1.2534	0.7402
2.4286	1.4720
-2.4286	-1.4720
2.4286	-1.4720
-2.4286	1.4720
4.6020	3.0856
-4.6020	-3.0856
4.6020	-3.0856
-4.6020	3.0856
2.4655	6.8054
-2.4655	-6.8054
2.4655	-6.8054
-2.4655	6.8054
4.6079	5.5572
-4.6079	-5.5572
4.6079	-5.5572
-4.6079	5.5572

-1.2122	3.4990
-1.2122	4.8980
-2.4241	5.5980
-0.0003	2.7990
1.2121	3.4990
1.2121	4.8980
-0.0003	5.5980
2.4240	2.7990
3.6360	3.4990
3.6360	4.8980
2.4240	5.5980
-2.4241	1.3995
-1.2122	0.6999
-0.0003	1.3995
1.2121	0.6999
2.4240	1.3995
-1.2122	-0.6996
-0.0003	-1.3996
1.2121	-0.6996
-0.0003	-2.7991
1.2121	-3.4986
2.4240	-2.7991
2.4240	-1.3996
-2.4241	-1.3996
-2.4241	-2.7991
-1.2122	-3.4986
-3.6360	-3.4986
-3.6360	-4.8981
-2.4241	-5.5981
-1.2122	-4.8981
-0.0003	-5.5981
1.2121	-4.8981
2.4240	-5.5981
3.6360	-4.8981
3.6360	-3.4986
-0.0003	6.9975
-1.2122	7.6975
-2.4241	6.9975
2.4240	6.9980
1.2121	7.6980
-2.4241	-6.9976
-1.2122	-7.6971
-0.0003	-6.9976
1.2121	-7.6981
2.4240	-6.9981
3.6360	-7.6971
4.8479	-6.9976
4.8479	-5.5981
-4.8479	-5.5981
-4.8479	-6.9976
-3.6360	-7.6976
4.8483	5.5980
4.8483	6.9975
3.6360	7.6975
-3.6360	7.6971
-4.8479	6.9975
-4.8479	5.5980

-1.2122	3.4989
-1.2122	4.8980
-2.4241	5.5979
-0.0003	2.7989
1.2121	3.4989
1.2121	4.8980
-0.0003	5.5979
2.4240	2.7989
3.6359	3.4989
3.6359	4.8980
2.4240	5.5979
-2.4241	1.3994
-1.2122	0.6999
-0.0003	1.3994
1.2121	0.6999
2.4240	1.3994
-1.2122	-0.6996
-0.0003	-1.3996
1.2121	-0.6996
-0.0003	-2.7991
1.2121	-3.4987
2.4240	-2.7991
2.4240	-1.3996
-2.4241	-1.3996
-2.4241	-2.7991
-1.2122	-3.4987
-3.6360	-3.4987
-3.6360	-4.8982
-2.4241	-5.5982
-1.2122	-4.8982
-0.0003	-5.5982
1.2121	-4.8982
2.4240	-5.5982
3.6359	-4.8982
3.6359	-3.4987
-0.0003	6.9975
-1.2122	7.6975
-2.4241	6.9975
2.4240	6.9979
1.2121	7.6979
-2.4241	-6.9977
-1.2122	-7.6972
-0.0003	-6.9977
1.2121	-7.6982
2.4240	-6.9982
3.6359	-7.6972
4.8479	-6.9977
4.8479	-5.5982
-4.8479	-5.5982
-4.8479	-6.9977
-3.6360	-7.6977
4.8483	5.5979
4.8483	6.9975
3.6359	7.6975
-3.6360	7.6970
-4.8479	6.9975
-4.8479	5.5979
-1.2122	9.0970
-2.4241	9.7965
-3.6360	9.0970
1.2121	9.0970
-0.0003	9.7970

3.6359	9.0974
2.4240	9.7974
6.0607	7.6975
6.0607	9.0974
4.8483	9.7974
-4.8479	9.7960
-6.0599	9.0965
-6.0599	7.6970
-3.6360	-9.0967
-2.4241	-9.7962
-1.2122	-9.0967
-0.0003	-9.7962
1.2121	-9.0967
2.4240	-9.7972
3.6359	-9.0977
4.8479	-9.7962
6.0598	-9.0967
6.0598	-7.6972
-6.0603	-7.6977
-6.0603	-9.0972
-4.8479	-9.7972

3-3-2 Coordinates	
**X**	**Y**
0.0000	0.7000
0.0000	-0.7000
1.2124	1.4000
-1.2124	-1.4000
1.2124	-1.4000
-1.2124	1.4000
0.0000	3.5000
0.0000	-3.5000
1.2124	2.8000
-1.2124	-2.8000
1.2124	-2.8000
-1.2124	2.8000
2.4249	3.5000
-2.4249	-3.5000
2.4249	-3.5000
-2.4249	3.5000
0.0000	4.9000
0.0000	-4.9000
1.2124	5.6000
-1.2124	-5.6000
1.2124	-5.6000
-1.2124	5.6000
2.4249	4.9000
-2.4249	-4.9000
2.4249	-4.9000
-2.4249	4.9000
2.4249	0.7000
-2.4249	-0.7000
2.4249	-0.7000
-2.4249	0.7000
3.6373	1.4000
-3.6373	-1.4000
3.6373	-1.4000
-3.6373	1.4000
3.6373	2.8000
-3.6373	-2.8000
3.6373	-2.8000

4-4-2 Coordinates	
**X**	**Y**
0.0000	0.7000
0.0000	-0.7000
1.2124	1.4000
-1.2124	-1.4000
1.2124	-1.4000
-1.2124	1.4000
0.0000	3.5000
0.0000	-3.5000
1.2124	2.8000
-1.2124	-2.8000
1.2124	-2.8000
-1.2124	2.8000
2.4249	3.5000
-2.4249	-3.5000
2.4249	-3.5000
-2.4249	3.5000
0.0000	4.9000
0.0000	-4.9000
1.2124	5.6000
-1.2124	-5.6000
1.2124	-5.6000
-1.2124	5.6000
2.4249	4.9000
-2.4249	-4.9000
2.4249	-4.9000
-2.4249	4.9000
2.4249	0.7000
-2.4249	-0.7000
2.4249	-0.7000
-2.4249	0.7000
3.6373	1.4000
-3.6373	-1.4000
3.6373	-1.4000
-3.6373	1.4000
3.6373	2.8000
-3.6373	-2.8000
3.6373	-2.8000

5-5-2 Coordinates	
**X**	**Y**
0.0000	0.7000
0.0000	-0.7000
0.0000	3.5000
0.0000	-3.5000
0.0000	4.9000
0.0000	-4.9000
0.0000	7.7000
0.0000	-7.7000
0.0000	9.1000
0.0000	-9.1000
1.2124	1.4000
-1.2124	-1.4000
1.2124	-1.4000
-1.2124	1.4000
1.2124	2.8000
-1.2124	-2.8000
1.2124	-2.8000
-1.2124	2.8000
1.2124	5.6000
-1.2124	-5.6000
1.2124	-5.6000
-1.2124	5.6000
1.2124	7.0000
-1.2124	-7.0000
1.2124	-7.0000
-1.2124	7.0000
1.2124	9.8000
-1.2124	-9.8000
1.2124	-9.8000
-1.2124	9.8000
2.4249	0.7000
-2.4249	-0.7000
2.4249	-0.7000
-2.4249	0.7000
2.4249	3.5000
-2.4249	-3.5000
2.4249	-3.5000

-3.6373	2.8000
3.6373	5.6000
-3.6373	-5.6000
3.6373	-5.6000
-3.6373	5.6000
4.8497	3.5000
-4.8497	-3.5000
4.8497	-3.5000
-4.8497	3.5000
4.8497	4.9000
-4.8497	-4.9000
4.8497	-4.9000
-4.8497	4.9000

-3.6373	2.8000
3.6373	5.6000
-3.6373	-5.6000
3.6373	-5.6000
-3.6373	5.6000
4.8497	3.5000
-4.8497	-3.5000
4.8497	-3.5000
-4.8497	3.5000
4.8497	4.9000
-4.8497	-4.9000
4.8497	-4.9000
-4.8497	4.9000
1.2124	7.0000
-1.2124	-7.0000
1.2124	-7.0000
-1.2124	7.0000
3.6373	7.0000
-3.6373	-7.0000
3.6373	-7.0000
-3.6373	7.0000
6.0622	7.0000
-6.0622	-7.0000
6.0622	-7.0000
-6.0622	7.0000
6.0622	5.6000
-6.0622	-5.6000
6.0622	-5.6000
-6.0622	5.6000
0.0000	7.7000
0.0000	-7.7000
2.4249	7.7000
-2.4249	-7.7000
2.4249	-7.7000
-2.4249	7.7000
4.8497	7.7000
-4.8497	-7.7000
4.8497	-7.7000
-4.8497	7.7000

-2.4249	3.5000
2.4249	4.9000
-2.4249	-4.9000
2.4249	-4.9000
-2.4249	4.9000
2.4249	7.7000
-2.4249	-7.7000
2.4249	-7.7000
-2.4249	7.7000
2.4249	9.1000
-2.4249	-9.1000
2.4249	-9.1000
-2.4249	9.1000
3.6373	1.4000
-3.6373	-1.4000
3.6373	-1.4000
-3.6373	1.4000
3.6373	2.8000
-3.6373	-2.8000
3.6373	-2.8000
-3.6373	2.8000
3.6373	5.6000
-3.6373	-5.6000
3.6373	-5.6000
-3.6373	5.6000
3.6373	7.0000
-3.6373	-7.0000
3.6373	-7.0000
-3.6373	7.0000
3.6373	9.8000
-3.6373	-9.8000
3.6373	-9.8000
-3.6373	9.8000
4.8497	3.5000
-4.8497	-3.5000
4.8497	-3.5000
-4.8497	3.5000
4.8497	4.9000
-4.8497	-4.9000
4.8497	-4.9000
-4.8497	4.9000
4.8497	7.7000
-4.8497	-7.7000
4.8497	-7.7000
-4.8497	7.7000
4.8497	9.1000
-4.8497	-9.1000
4.8497	-9.1000
-4.8497	9.1000
6.0622	5.6000
-6.0622	-5.6000
6.0622	-5.6000
-6.0622	5.6000
6.0622	7.0000
-6.0622	-7.0000
6.0622	-7.0000
-6.0622	7.0000
6.0622	9.8000
-6.0622	-9.8000
6.0622	-9.8000
-6.0622	9.8000
7.2746	7.7000

-7.2746	-7.7000
7.2746	-7.7000
-7.2746	7.7000
7.2746	9.1000
-7.2746	-9.1000
7.2746	-9.1000
-7.2746	9.1000

3-3-3 Coordinates		4-4-3 Coordinates		5-5-1 Coordinates	
**X**	**Y**	**X**	**Y**	**X**	**Y**
0.0000	1.4000	0.0000	1.4000	0.0000	1.4000
0.0000	-1.4000	0.0000	-1.4000	0.0000	-1.4000
1.2124	0.7000	1.2124	0.7000	0.0000	2.8000
-1.2124	-0.7000	-1.2124	-0.7000	0.0000	-2.8000
1.2124	-0.7000	1.2124	-0.7000	0.0000	5.6000
-1.2124	0.7000	-1.2124	0.7000	0.0000	-5.6000
2.4249	1.4000	2.4249	1.4000	0.0000	7.0000
-2.4249	-1.4000	-2.4249	-1.4000	0.0000	-7.0000
2.4249	-1.4000	2.4249	-1.4000	0.0000	9.8000
-2.4249	1.4000	-2.4249	1.4000	0.0000	-9.8000
0.0000	2.8000	0.0000	2.8000	1.2124	0.7000
0.0000	-2.8000	0.0000	-2.8000	-1.2124	-0.7000
1.2124	3.5000	1.2124	3.5000	1.2124	-0.7000
-1.2124	-3.5000	-1.2124	-3.5000	-1.2124	0.7000
1.2124	-3.5000	1.2124	-3.5000	1.2124	3.5000
-1.2124	3.5000	-1.2124	3.5000	-1.2124	-3.5000
2.4249	2.8000	2.4249	2.8000	1.2124	-3.5000
-2.4249	-2.8000	-2.4249	-2.8000	-1.2124	3.5000
2.4249	-2.8000	2.4249	-2.8000	1.2124	4.9000
-2.4249	2.8000	-2.4249	2.8000	-1.2124	-4.9000
3.6373	3.5000	3.6373	3.5000	1.2124	-4.9000
-3.6373	-3.5000	-3.6373	-3.5000	-1.2124	4.9000
3.6373	-3.5000	3.6373	-3.5000	1.2124	7.7000
-3.6373	3.5000	-3.6373	3.5000	-1.2124	-7.7000
0.0000	5.6000	0.0000	5.6000	1.2124	-7.7000
0.0000	-5.6000	0.0000	-5.6000	-1.2124	7.7000
1.2124	4.9000	1.2124	4.9000	1.2124	9.1000
-1.2124	-4.9000	-1.2124	-4.9000	-1.2124	-9.1000
1.2124	-4.9000	1.2124	-4.9000	1.2124	-9.1000
-1.2124	4.9000	-1.2124	4.9000	-1.2124	9.1000
2.4249	5.6000	2.4249	5.6000	2.4249	1.4000
-2.4249	-5.6000	-2.4249	-5.6000	-2.4249	-1.4000
2.4249	-5.6000	2.4249	-5.6000	2.4249	-1.4000
-2.4249	5.6000	-2.4249	5.6000	-2.4249	1.4000
3.6373	4.9000	3.6373	4.9000	2.4249	2.8000
-3.6373	-4.9000	-3.6373	-4.9000	-2.4249	-2.8000
3.6373	-4.9000	3.6373	-4.9000	2.4249	-2.8000
-3.6373	4.9000	-3.6373	4.9000	-2.4249	2.8000
3.6373	0.7000	3.6373	0.7000	2.4249	5.6000
-3.6373	-0.7000	-3.6373	-0.7000	-2.4249	-5.6000
3.6373	-0.7000	3.6373	-0.7000	2.4249	-5.6000
-3.6373	0.7000	-3.6373	0.7000	-2.4249	5.6000
4.8497	1.4000	4.8497	1.4000	2.4249	7.0000
-4.8497	-1.4000	-4.8497	-1.4000	-2.4249	-7.0000
4.8497	-1.4000	4.8497	-1.4000	2.4249	-7.0000
-4.8497	1.4000	-4.8497	1.4000	-2.4249	7.0000
4.8497	2.8000	4.8497	2.8000	2.4249	9.8000
-4.8497	-2.8000	-4.8497	-2.8000	-2.4249	-9.8000
4.8497	-2.8000	4.8497	-2.8000	2.4249	-9.8000
-4.8497	2.8000	-4.8497	2.8000	-2.4249	9.8000
4.8497	5.6000	4.8497	5.6000	3.6373	0.7000
-4.8497	-5.6000	-4.8497	-5.6000	-3.6373	-0.7000

4.8497	-5.6000
-4.8497	5.6000
6.0622	3.5000
-6.0622	-3.5000
6.0622	-3.5000
-6.0622	3.5000
6.0622	4.9000
-6.0622	-4.9000
6.0622	-4.9000
-6.0622	4.9000

4.8497	-5.6000
-4.8497	5.6000
6.0622	3.5000
-6.0622	-3.5000
6.0622	-3.5000
-6.0622	3.5000
6.0622	4.9000
-6.0622	-4.9000
6.0622	-4.9000
-6.0622	4.9000
0.0000	7.0000
0.0000	-7.0000
2.4249	7.0000
-2.4249	-7.0000
2.4249	-7.0000
-2.4249	7.0000
4.8497	7.0000
-4.8497	-7.0000
4.8497	-7.0000
-4.8497	7.0000
7.2746	5.6000
-7.2746	-5.6000
7.2746	-5.6000
-7.2746	5.6000
7.2746	7.0000
-7.2746	-7.0000
7.2746	-7.0000
-7.2746	7.0000
1.2124	7.7000
-1.2124	-7.7000
1.2124	-7.7000
-1.2124	7.7000
3.6373	7.7000
-3.6373	-7.7000
3.6373	-7.7000
-3.6373	7.7000
6.0622	7.7000
-6.0622	-7.7000
6.0622	-7.7000
-6.0622	7.7000

3.6373	-0.7000
-3.6373	0.7000
3.6373	3.5000
-3.6373	-3.5000
3.6373	-3.5000
-3.6373	3.5000
3.6373	4.9000
-3.6373	-4.9000
3.6373	-4.9000
-3.6373	4.9000
3.6373	7.7000
-3.6373	-7.7000
3.6373	-7.7000
-3.6373	7.7000
3.6373	9.1000
-3.6373	-9.1000
3.6373	-9.1000
-3.6373	9.1000
4.8497	1.4000
-4.8497	-1.4000
4.8497	-1.4000
-4.8497	1.4000
4.8497	2.8000
-4.8497	-2.8000
4.8497	-2.8000
-4.8497	2.8000
4.8497	5.6000
-4.8497	-5.6000
4.8497	-5.6000
-4.8497	5.6000
4.8497	7.0000
-4.8497	-7.0000
4.8497	-7.0000
-4.8497	7.0000
4.8497	9.8000
-4.8497	-9.8000
4.8497	-9.8000
-4.8497	9.8000
6.0622	3.5000
-6.0622	-3.5000
6.0622	-3.5000
-6.0622	3.5000
6.0622	4.9000
-6.0622	-4.9000
6.0622	-4.9000
-6.0622	4.9000
6.0622	7.7000
-6.0622	-7.7000
6.0622	-7.7000
-6.0622	7.7000
6.0622	9.1000
-6.0622	-9.1000
6.0622	-9.1000
-6.0622	9.1000
7.2746	5.6000
-7.2746	-5.6000
7.2746	-5.6000
-7.2746	5.6000
7.2746	7.0000
-7.2746	-7.0000
7.2746	-7.0000
-7.2746	7.0000

7.2746	9.8000
-7.2746	-9.8000
7.2746	-9.8000
-7.2746	9.8000
8.4871	7.7000
-8.4871	-7.7000
8.4871	-7.7000
-8.4871	7.7000
8.4871	9.1000
-8.4871	-9.1000
8.4871	-9.1000
-8.4871	9.1000

5-3-2 Coordinates	
**X**	**Y**
-6.0622	9.8000
-3.6373	9.8000
-1.2124	9.8000
1.2124	9.8000
3.6373	9.8000
6.0622	9.8000
-7.2746	9.1000
-4.8497	9.1000
-2.4249	9.1000
0.0000	9.1000
2.4249	9.1000
4.8497	9.1000
7.2746	9.1000
-7.2746	7.7000
-4.8497	7.7000
-2.4249	7.7000
0.0000	7.7000
2.4249	7.7000
4.8497	7.7000
7.2746	7.7000
-6.0622	7.0000
-3.6373	7.0000
-1.2124	7.0000
1.2124	7.0000
3.6373	7.0000
6.0622	7.0000
-6.0622	5.6000
-3.6373	5.6000
-1.2124	5.6000
1.2124	5.6000
3.6373	5.6000
6.0622	5.6000
-4.8497	4.9000
-2.4249	4.9000
0.0000	4.9000
2.4249	4.9000
4.8497	4.9000
-4.8497	3.5000
-2.4249	3.5000
0.0000	3.5000
2.4249	3.5000
4.8497	3.5000
-3.6373	2.8000
-1.2124	2.8000
1.2124	2.8000
3.6373	2.8000

5-4-2 Coordinates	
**X**	**Y**
7.2746	9.1000
7.2746	7.7000
6.0622	9.8000
6.0622	7.0000
6.0622	5.6000
6.0622	-5.6000
6.0622	-7.0000
4.8497	9.1000
4.8497	7.7000
4.8497	4.9000
4.8497	3.5000
4.8497	-3.5000
4.8497	-4.9000
4.8497	-7.7000
3.6373	9.8000
3.6373	5.6000
3.6373	2.8000
3.6373	1.4000
3.6373	-1.4000
3.6373	-2.8000
3.6373	-5.6000
3.6373	7.0000
3.6373	-7.0000
2.4249	9.1000
2.4249	7.7000
2.4249	4.9000
2.4249	3.5000
2.4249	0.7000
2.4249	-0.7000
2.4249	-3.5000
2.4249	-4.9000
2.4249	-7.7000
1.2124	9.8000
1.2124	7.0000
1.2124	5.6000
1.2124	2.8000
1.2124	1.4000
1.2124	-1.4000
1.2124	-2.8000
1.2124	-5.6000
1.2124	-7.0000
0.0000	4.9000
0.0000	3.5000
0.0000	0.7000
0.0000	-0.7000
0.0000	-3.5000

-3.6373	1.4000
-1.2124	1.4000
1.2124	1.4000
3.6373	1.4000
-2.4249	0.7000
0.0000	0.7000
2.4249	0.7000
-2.4249	-0.7000
0.0000	-0.7000
2.4249	-0.7000
-3.6373	-1.4000
-1.2124	-1.4000
1.2124	-1.4000
3.6373	-1.4000
-3.6373	-2.8000
-1.2124	-2.8000
1.2124	-2.8000
3.6373	-2.8000
-4.8497	-3.5000
-2.4249	-3.5000
0.0000	-3.5000
2.4249	-3.5000
4.8497	-3.5000
-4.8497	-4.9000
-2.4249	-4.9000
0.0000	-4.9000
2.4249	-4.9000
4.8497	-4.9000
-3.6373	-5.6000
-1.2124	-5.6000
1.2124	-5.6000
3.6373	-5.6000

0.0000	-4.9000
0.0000	9.1000
0.0000	7.7000
0.0000	-7.7000
-1.2124	7.0000
-1.2124	-7.0000
-1.2124	9.8000
-1.2124	5.6000
-1.2124	2.8000
-1.2124	1.4000
-1.2124	-1.4000
-1.2124	-2.8000
-1.2124	-5.6000
-2.4249	9.1000
-2.4249	7.7000
-2.4249	-7.7000
-2.4249	4.9000
-2.4249	3.5000
-2.4249	0.7000
-2.4249	-0.7000
-2.4249	-3.5000
-2.4249	-4.9000
-3.6373	7.0000
-3.6373	-7.0000
-3.6373	9.8000
-3.6373	5.6000
-3.6373	2.8000
-3.6373	1.4000
-3.6373	-1.4000
-3.6373	-2.8000
-3.6373	-5.6000
-4.8497	9.1000
-4.8497	7.7000
-4.8497	-7.7000
-4.8497	4.9000
-4.8497	3.5000
-4.8497	-3.5000
-4.8497	-4.9000
-6.0622	7.0000
-6.0622	5.6000
-6.0622	-5.6000
-6.0622	-7.0000
-6.0622	9.8000
-7.2746	9.1000
-7.2746	7.7000

End of the Supplementary Information
